# Patient and Hospital Factors Associated With Differences in Mortality Rates Among Black and White US Medicare Beneficiaries Hospitalized With COVID-19 Infection

**DOI:** 10.1001/jamanetworkopen.2021.12842

**Published:** 2021-06-17

**Authors:** David A. Asch, Md Nazmul Islam, Natalie E. Sheils, Yong Chen, Jalpa A. Doshi, John Buresh, Rachel M. Werner

**Affiliations:** 1 Division of General Internal Medicine, University of Pennsylvania, Philadelphia; 2 Leonard Davis Institute of Health Economics, University of Pennsylvania, Philadelphia; 3 UnitedHealth Group, Minnetonka, Minnesota; 4 Department of Biostatistics, Epidemiology, and Informatics, University of Pennsylvania, Philadelphia; 5 Cpl Michael J. Crescenz VA Medical Center, Philadelphia, Pennsylvania

## Abstract

**Importance:**

Black patients hospitalized with COVID-19 may have worse outcomes than White patients because of excess individual risk or because Black patients are disproportionately cared for in hospitals with worse outcomes for all.

**Objectives:**

To examine differences in COVID-19 hospital mortality rates between Black and White patients and to assess whether the mortality rates reflect differences in patient characteristics by race or by the hospitals to which Black and White patients are admitted.

**Design, Setting, and Participants:**

This cohort study assessed Medicare beneficiaries admitted with a diagnosis of COVID-19 to 1188 US hospitals from January 1, 2020, through September 21, 2020.

**Exposure:**

Hospital admission for a diagnosis of COVID-19.

**Main Outcomes and Measures:**

The primary composite outcome was inpatient death or discharge to hospice within 30 days of admission. We estimated the association of patient-level characteristics (including age, sex, zip code–level income, comorbidities, admission from a nursing facility, and days since January 1, 2020) with differences in mortality or discharge to hospice among Black and White patients. To examine the association with the hospital itself, we adjusted for the specific hospitals to which patients were admitted. We used simulation modeling to estimate the mortality among Black patients had they instead been admitted to the hospitals where White patients were admitted.

**Results:**

Of the 44 217 Medicare beneficiaries included in the study, 24 281 (55%) were women; mean (SD) age was 76.3 (10.5) years; 33 459 participants (76%) were White, and 10 758 (24%) were Black. Overall, 2634 (8%) White patients and 1100 (10%) Black patients died as inpatients, and 1670 (5%) White patients and 350 (3%) Black patients were discharged to hospice within 30 days of hospitalization, for a total mortality-equivalent rate of 12.86% for White patients and 13.48% for Black patients. Black patients had similar odds of dying or being discharged to hospice (odds ratio [OR], 1.06; 95% CI, 0.99-1.12) in an unadjusted comparison with White patients. After adjustment for clinical and sociodemographic patient characteristics, Black patients were more likely to die or be discharged to hospice (OR, 1.11; 95% CI, 1.03-1.19). This difference became indistinguishable when adjustment was made for the hospitals where care was delivered (odds ratio, 1.02; 95% CI, 0.94-1.10). In simulations, if Black patients in this sample were instead admitted to the same hospitals as White patients in the same distribution, their rate of mortality or discharge to hospice would decline from the observed rate of 13.48% to the simulated rate of 12.23% (95% CI for difference, 1.20%-1.30%).

**Conclusions and Relevance:**

This cohort study found that Black patients hospitalized with COVID-19 had higher rates of hospital mortality or discharge to hospice than White patients after adjustment for the personal characteristics of those patients. However, those differences were explained by differences in the hospitals to which Black and White patients were admitted.

## Key Points

**Question:**

Do Black patients hospitalized with COVID-19 have worse outcomes than White patients and, if so, what is the association between worse outcomes and comorbidities vs the hospitals to which they are admitted?

**Findings:**

In this cohort study of 44 217 adult Medicare beneficiaries admitted with COVID-19 to 1188 US hospitals, odds of 30-day inpatient mortality or discharge to hospice were 11% higher for Black patients than for White patients after adjustment for patient sociodemographic and clinical characteristics. That difference was largely eliminated when adjustment was made for the hospital where care was received.

**Meaning:**

This study’s findings suggest that the increased mortality among Black patients hospitalized with COVID-19 is associated with the hospitals at which Black patients disproportionately received care.

## Teaser

This cohort study examines differences in COVID-19 hospital mortality rates between Black and White patients and assesses whether they reflect differences in patient characteristics by race or by the hospitals to which Black and White patients are admitted.

## Introduction

Throughout the COVID-19 pandemic, Black people have been more likely than White people to become infected with coronavirus, to be hospitalized with COVID-19, and to die.^1^ In most early studies of hospitalized patients, differences in mortality by race have not been found after statistical adjustment for patient-level characteristics.^2,3,4,5^

However, even if statistical adjustment for patient characteristics explains racial differences in outcome, it does not excuse them if those factors are disproportionately represented in Black populations as a result of discrimination. If so, adjusting for such factors risks obscuring, rather than highlighting, the structural mechanisms that disadvantage Black patients. Racial differences in the outcomes of patients with COVID-19 might also arise if Black patients disproportionately receive care at hospitals delivering worse care for all.^6,7,8,9,10^

Using a large national data set of hospitalized Medicare beneficiaries with COVID-19, we examined differences in mortality rates between Black and White patients. Unlike prior studies based on data from a single health care system, our study included 44 217 patients admitted with COVID-19 to a diverse set of 1188 hospitals in 41 states and focused explicitly on isolating the association of mortality with patient-level factors and the admitting hospital.

## Methods

### Data Sources

For this cohort study, we used deidentified administrative hospitalization claims from a large national commercial health insurer in the United States, including primary or secondary diagnosis of COVID-19 (eTable 2 in the Supplement) and each patient’s disposition on each day of the hospitalization and at the time of hospital discharge (admitted, discharged, transferred, or expired) from January 1 through September 21, 2020. We obtained hospital-level characteristics from the 2020 US Centers for Medicare & Medicaid Services provider of service files.^11^ Race was based on data reported by the Centers for Medicare & Medicaid Services, and income data were the median reported income at a zip code level based on the 2017 census. This study was reviewed and deemed exempt by the institutional review board of UnitedHealth Group. This study followed the Strengthening the Reporting of Observational Studies in Epidemiology (STROBE) reporting guideline.

### Patients and Hospitals

We started by collecting data on all Medicare Advantage enrollees 18 years or older who were hospitalized with COVID-19 since January 1, 2020 (eFigure 12 in the Supplement). We excluded patients with fewer than 6 months of insurance enrollment in 2019 (to permit comorbidity measurement using historical claims), those with incomplete information for race or zip code, those admitted with COVID-19 after August 22, 2020 (to provide sufficient follow-up to measure 30-day event rates), those admitted to a hospital not listed as an acute care hospital in the Centers for Medicare & Medicaid Services provider of service files^11^ that also had at least 1 Black and 1 White patient admitted from our sample, and those who were readmitted to or transferred to another hospital within 30 days of initial admission (to prevent misattributing hospital-level outcomes among patients admitted to multiple hospitals). Our final sample included all Black and White patients admitted with a diagnosis of COVID-19 to an acute care hospital that admitted at least 1 Black patient and 1 White patient with COVID-19 (Table 1).

**Table 1.** Sample Characteristics

Characteristic	No. (%)		
	Total sample (N = 44 217)	Black patients (n = 10 758)	White patients (n = 33 459)
**Patient-level characteristics**			
Age, mean (SD), y	76.3 (10.5)	73.5 (10.9)	77.3 (10.2)
Age range, y			
18-55	1458 (3)	623 (7)	835 (2)
>55-65	4173 (9)	1426(13)	2747 (8)
>65-75	14 761 (33)	4107 (38)	10 654 (29)
>75-85	14 900 (34)	3191 (30)	11 709 (32)
>85	8925 (20)	1411 (13)	7514 (22)
Sex			
Male	19 936 (45)	4398 (41)	15 538 (46)
Female	24 281 (55)	6360 (59)	17 921 (54)
Elixhauser score, mean (SD)	9.8 (11.3)	10.5 (11.8)	9.5 (11.1)
Elixhauser comorbidities			
AIDS	193 (0)	110 (1)	83 (0)
Alcohol use disorder	1410 (3)	370 (3)	1040 (3)
Iron deficiency anemia	16 747 (38)	4884 (45)	11 863 (35)
Rheumatoid arthritis	4124 (9)	966 (9)	3158 (9)
Blood loss anemia	2013 (5)	501 (5)	1512 (5)
Congestive heart failure	13 423 (30)	3696 (34)	9727 (29)
Chronic obstructive pulmonary disease	16 589 (38)	3804 (35)	12 785 (38)
Coagulopathy	3553 (8)	857 (8)	2696 (8)
Depression	10 671 (24)	1965 (18)	8706 (26)
Diabetes			
Without chronic complication	18 860 (43)	6017 (56)	12 843 (38)
With chronic complication	16 347 (37)	5318 (49)	11 029 (33)
Substance use disorder	1717 (4)	438 (4)	1279 (4)
Hypertension	38 408 (87)	9963 (93)	28 445 (85)
Hypothyroidism	10 971 (25)	1782 (17)	9189 (27)
Lymphoma	885 (2)	215 (2)	670 (2)
Fluid and electrolyte disorder	13 480 (30)	3557 (33)	9923 (30)
Metastatic cancer	1619 (4)	356 (3)	1263 (4)
Neurologic disorder	12 781 (29)	3024 (28)	9757 (29)
Obesity	12 455 (28)	3714 (35)	8741 (26)
Paralysis	2895 (7)	1056 (10)	1839 (5)
Peripheral vascular disease	13 956 (32)	3267 (30)	10 689 (32)
Psychosis	3496 (8)	774 (7)	2722 (8)
Chronic kidney disease	13 835 (31)	4231 (39)	9604 (29)
Solid tumor without metastasis	6762 (15)	1515 (14)	5247 (16)
Valvular disorder	10 434 (24)	2272 (21)	8162 (24)
Weight loss	4593 (10)	1225 (11)	3368 (10)
Income by zip code, mean (SD), $			
<50 000	14 781 (33)	4941 (46)	9840 (29)
50 000-69 999	20 877 (47)	4565 (42)	16 312 (49)
≥70 000	8559 (19)	1252 (12)	7307 (22)
Transferred from a nursing facility	7739 (18)	1969 (18)	5770 (17)
Days since January 1, 2020, to admission, mean (SD)	160.9 (44.5)	157.7 (45.6)	161.9 (44.0)
Died	3734 (8)	1100 (10)	2634 (8)
Transferred to hospice	2029 (5)	350 (3)	1670 (5)
**Hospital-level characteristics**			
Hospital size, No. of beds			
0-149	7530 (17)	1521 (14)	6009 (18)
150-299	9014 (20)	2183 (20)	6831 (20)
300-449	9248 (21)	2111 (20)	7137 (21)
≥450	18 425 (42)	4943 (46)	13 482 (40)
Hospital setting			
Urban	22 430 (51)	5581 (52)	16 849 (50)
Nonurban	21 787 (49)	5177 (48)	16 610 (50)
Hospital region			
Northeast	11 603 (26)	2294 (21)	9309 (28)
South	16 987 (38)	5760 (54)	11 227 (34)
Midwest	13 697 (31)	2558 (24)	11 139 (33)
West	1930 (4)	146 (1)	1784 (5)
Profit status			
Nonprofit	32 151 (73)	7208 (67)	24 943 (75)
For profit	5552 (13)	1473 (14)	4079 (12)
Other	6514 (15)	2077 (19)	4437 (13)

### Outcome Measure

Our main outcome measure was the composite of either inpatient mortality or discharge to hospice within 30 days of initial admission for COVID-19. We considered this composite measure a more complete representation of the outcome of interest than mortality alone because it reflects an outcome closer to 30-day any-site mortality, given known racial differences in hospice use.^12^

### Statistical Analysis

We compared unadjusted composite outcomes of 30-day inpatient mortality or hospice discharge between Black and White patients (model A, Table 2). To examine how much of the difference in those outcomes could be explained by differences in the patient-level sociodemographic characteristics and the admitting hospital, we estimated 7 nested logistic regression models (Table 2) after adjustment in sequence for the following covariates: age and sex (model B); zip code–level income (model C); comorbidities and nursing facility admission source (model D); number of days between January 1, 2020, and the date of admission to account for likely improvements in patient outcome as hospitals gained experience (model E); census region to account for geographic variation in care and COVID-19 surges over time (model F); and covariates in model E plus hospital-level fixed effects (model G). We also modeled racial differences in the outcome after adjustment for state fixed effects in place of hospital fixed effects and only for hospital-level fixed effects and no patient characteristics.

**Table 2.** Unadjusted and Adjusted Probabilities and Odds Ratios for COVID-19 Inpatient Mortality or Discharge to Hospice Among Black Patients Compared With White Patients

Model	30-d Inpatient mortality or discharge to hospice (%)		Odds of 30-d inpatient mortality or discharge to hospice for Black patients compared with White patients (95% CI)	*P* value
	Black patients	White patients		
A. Unadjusted 30-d inpatient mortality or discharge to hospice	13.48	12.86	1.06 (0.99-1.12)	.10
B. Adjusted 30-d inpatient mortality or discharge to hospice controlling for age and sex	14.78	12.82	1.23 (1.16-1.32)	<.001
C. Same as model B plus patient-level income	14.61	12.63	1.22 (1.14-1.30)	<.001
D. Same as model C plus comorbidities and whether the patient was admitted from a nursing home^a^	13.16	11.53	1.17 (1.09-1.25)	<.001
E. Same as model D plus days between admission and January 1, 2020	12.32	11.27	1.11 (1.03-1.19)	.005
F. Same as model E plus region of hospital	12.24	11.27	1.10 (1.02-1.18)	.01
G. Same as model E plus hospital fixed effects	11.69	11.52	1.02 (0.94-1.10)	.71

^a^ Comorbidity adjustment includes 29 conditions from the Elixhauser comorbidity index^13^ and whether the patient was admitted from a nursing home.

In addition, we conducted a simulation to investigate how the observed 30-day inpatient mortality or hospice discharge rate for Black patients would change had they been admitted to the hospitals in our sample based on the same distribution as the White patients in our study, while retaining their sociodemographic and clinical characteristics. To do so, we assigned each Black patient to one of the 1188 US hospitals, using a multinomial distribution with probabilities estimated from the proportion of White patients distributed over these hospitals, and estimated individual risk of death or discharge to hospice if the patient were admitted to that assigned hospital. We used the mean of the estimated patient-level risks to estimate the overall risk for the population based on this new hospital assignment. We repeated the procedure 1000 times to obtain estimates of uncertainty (section A.4 of eAppendix 1 in the Supplement).

All statistical tests were 2-sided, with statistical significance set at *P* < .05. All analyses were conducted using R, version 3.6.3 (R Foundation for Statistical Computing).^14^ Statistical codes along with an illustration based on simulated data are included in eAppendix 2 in the Supplement.

## Results

**Figure 1.**
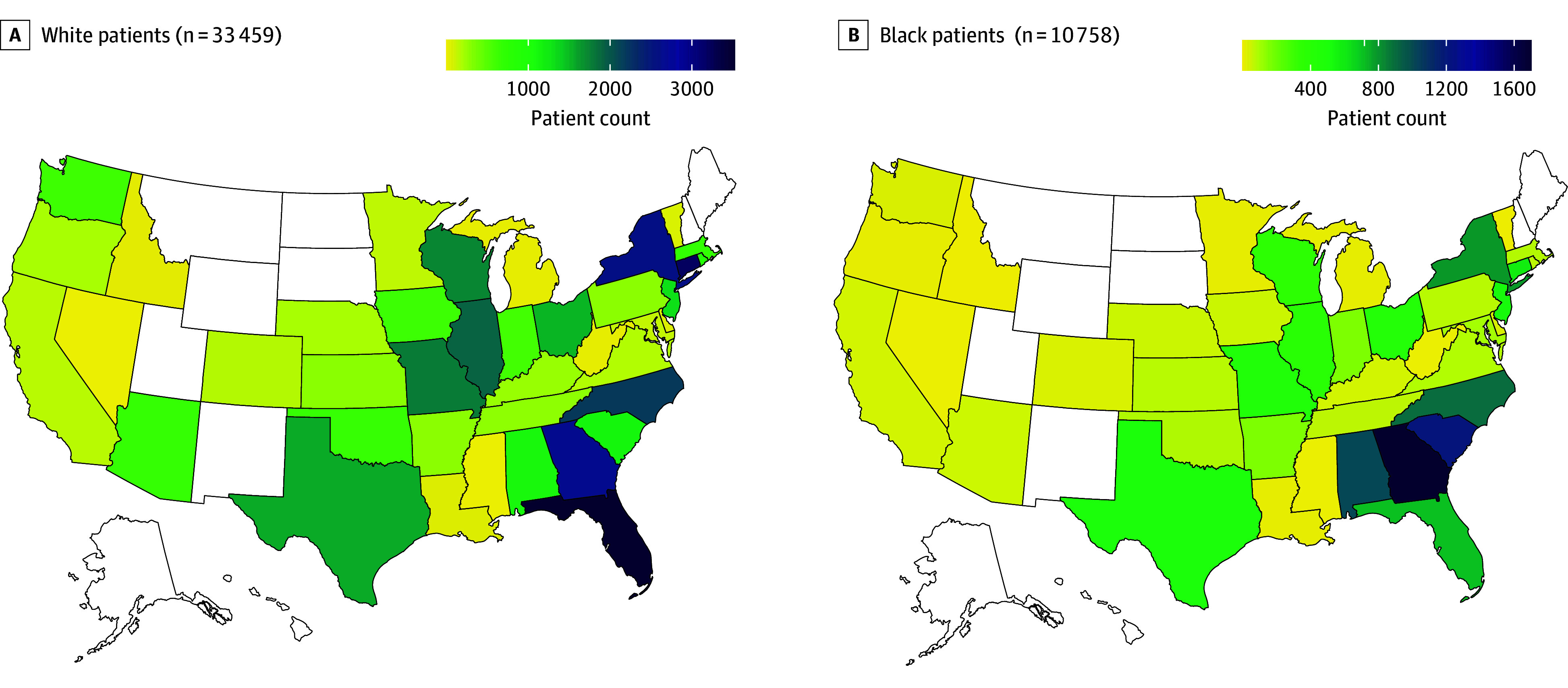
Geographic Distribution and Number of Black and White Patients Hospitalized With COVID-19 in the Sample

**Figure 2.**
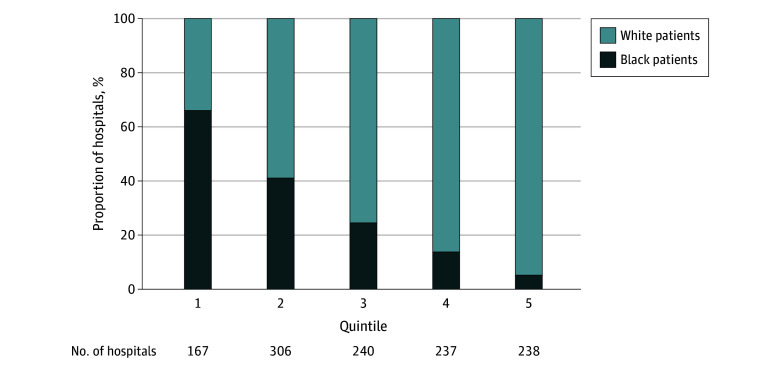
Distribution of Black and White Patients Across 1118 Hospitals The 1118 hospitals are divided into quintiles by the relative proportion of Black and White patients from the sample admitted to them; that is, the x-axis represents the differences in proportion of Black patients in our sample within a hospital. Each column shows the split of Black and White patients in the sample who were admitted to hospitals in that quintile. Note that quintiles are found on the hospital level and as such may contain different numbers of patients. Quintile 1 (hospital patient population comprises >50.0% Black patients) includes 5455 patients; quintile 2 (patient population comprises 30.0%-50.0% Black patients), 7483 patients; quintile 3 (patient population comprises 19.0%-30.0% Black patients), 8287 patients; quintile 4 (patient population comprises 9.2%-19.0% Black patients), 9887 patients; and quintile 5 (patient population comprises <9.2% Black patients), 13 105 patients.

Included in the analysis were 44 217 patients (33 459 [76%] White patients and 10 758 [24%] Black patients) admitted to 1188 hospitals in 41 states and the District of Columbia (Table 1 and Figure 1). The cohort included 19 936 (45%) men and 24 281 (55%) women and had a mean (SD) age of 76.3 (10.5) years. Black patients were more likely than White patients to be younger and female (characteristics associated with better outcomes^15^) and had more comorbidities (associated with worse outcomes^15^). The proportion of Black patients in study hospitals ranged from 33% in quintile 1 (hospitals with the highest proportion of Black patients) to 6% in quintile 5 (hospitals with the lowest proportion of Black patients), indicating that Black and White patients were distributed differently across hospitals (Figure 2).

Overall, 2634 (8%) White patients and 1100 (10%) Black patients died as inpatients, and 1670 (5%) White patients and 350 (3%) Black patients were discharged to hospice within 30 days of hospitalization, for a total inpatient mortality or discharge-to-hospice rate of 12.86% for White patients and 13.48% for Black patients. Without adjustment, Black patients had similar odds of 30-day inpatient mortality or discharge to hospice compared with White patients (odds ratio [OR], 1.06; 95% CI, 0.99-1.12; *P* = .10) (model A, Table 2; eFigure 1 in the Supplement).

Compared with the unadjusted model (model A in Table 2), adjustments for age and sex increased estimates of the mortality-equivalent disparity (model B, Table 2; eFigure 2 in the Supplement) because the characteristics of older age and male sex known to increase risk were less frequently represented in the Black patients in this sample.

Sequential adjustment (Table 2 and eFigures 1-11 and eTable 1 in the Supplement) for income and comorbidities and then nursing home source, in models C and D, progressively reduced observed differences between Black and White mortality equivalents (eFigures 3 and 4 in the Supplement) because these characteristics associated with poor COVID-19 outcomes were disproportionately represented among Black patients in the sample. Similarly, adjusting for date of admission (model E, Table 2; eFigure 5 in the Supplement) reduced observed differences because Black patients in our sample were disproportionately admitted during earlier periods, when case fatality ratios in hospitals were higher.^15^ Adjusting for region effects (model F, Table 2; eFigure 6 in the Supplement) further reduced the differences in the composite outcome by race.

After adjustment for age, sex, clinical comorbidities, income by zip code, and days between admission date and January 1, 2020, Black patients had greater odds of 30-day inpatient mortality or discharge to hospice compared with White patients (OR, 1.11; 95% CI, 1.03-1.19; *P* = .005). That difference equated to an adjusted risk of mortality of 12.32% for Black patients compared with 11.27% for White patients.

After further adjustment for hospital-level fixed effects (model G, Table 2; eFigure 7 in the Supplement), which accounted for differences in the admitting hospital, the odds of mortality or the equivalent was not statistically different for Black patients compared with White patients (OR, 1.02; 95% CI, 0.94-1.10; *P* = .71).

In sensitivity analyses, we first adjusted for state fixed effects in place of hospital fixed effects and found that the odds of mortality or the equivalent is not statistically different for Black patients compared with White patients (OR, 1.06; 95% CI, 0.99-1.14; *P* = .10) (eFigure 8 in the Supplement), suggesting that hospital factors associated with differences in outcomes across races were associated with states. When adjustments were made for hospital fixed effects, but not individual characteristics of the patients (eFigure 9 in the Supplement), the OR was 0.94 and was not statistically significant (95% CI, 0.87-1.01; *P* = .12), which suggests that the increased mortality or discharge-to-hospice rate among Black patients admitted with COVID-19 was largely attributable to the hospitals at which these patients received care.

**Figure 3.**
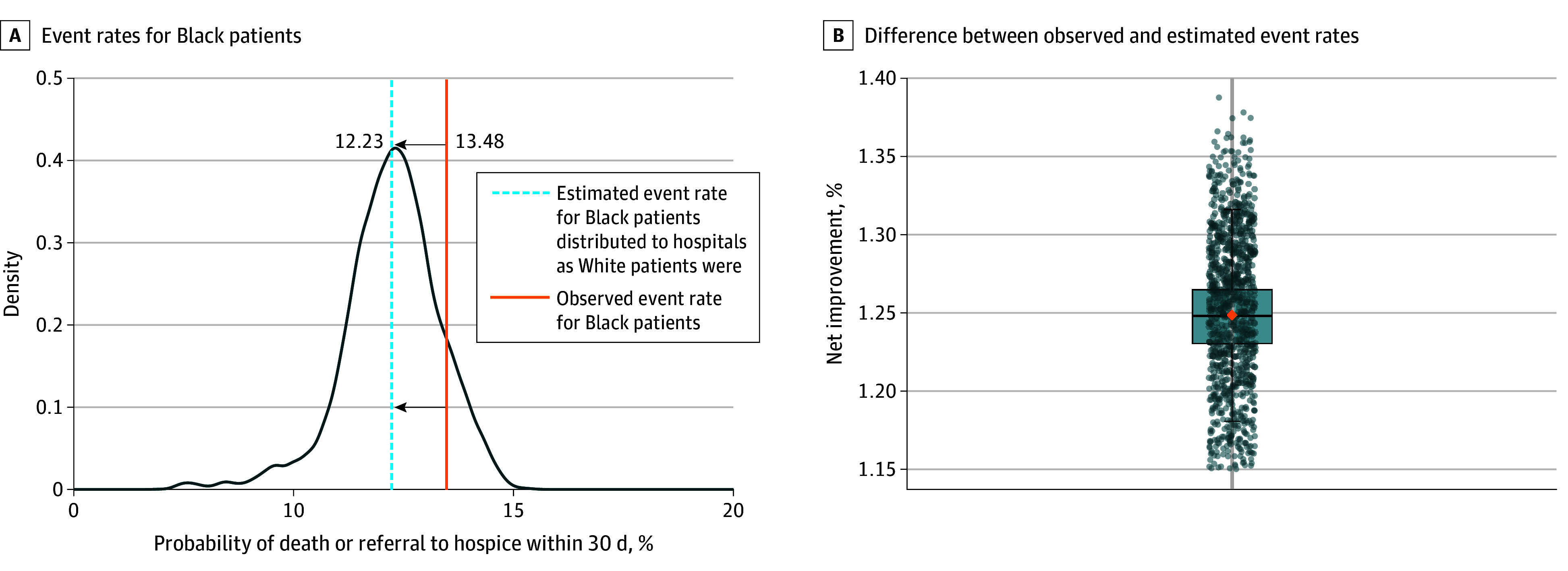
Simulated Improvement in Population Mortality for 10 758 Black Patients Had They Been Admitted to the Same Hospitals as White Patients The distribution reflects a simulation of 1000 replications of the estimated mean event rate had the Black patients been distributed as the White patients were. A, The solid vertical line corresponds to an observed rate of mortality or discharge to hospice of 13.48% for Black patients. The dashed blue line corresponds to an estimated rate of mortality or discharge to hospice of 12.23%. B, Difference between observed and estimated event rates for each of 1000 simulations. The orange diamond corresponds to the mean value of 1.25%. The horizontal line in the middle of the box indicates the median, and the box indicates the first and third quartiles.

In the simulation, had the Black patients in our sample been admitted to the same hospitals and in the same distribution as the White patients, their overall population risk of 30-day inpatient mortality or discharge to hospice would have declined from the observed 13.48% to the simulated 12.23% (absolute risk reduction, 1.25%; 95% CI, 1.20%-1.30%) (Figure 3).

## Discussion

In what is, to our knowledge, the largest and most geographically diverse US study to date, we found that Black patients admitted with COVID-19 had a higher mortality rate than White patients and that this difference was attributable to the different hospitals to which Black and White patients were admitted. Differences in mortality between Black and White patients hospitalized with COVID-19 have been examined in many studies, and mortality was not found to be statistically different between the groups when adjustments were made for the individual clinical and sociodemographic characteristics of patients or the site of care. However, our study, which included 44 217 patients admitted to 1188 hospitals in 41 states, differs from this prior work in 3 important ways.

First, we found that even after adjustment for individual patient characteristics, such as age, sex, income, comorbidities, nursing home or community admission source, and time since the onset of the pandemic, Black patients were more likely than White patients to die in the hospital or be discharged to hospice within 30 days of admission. Previous studies showed that racial mortality differences vanished with individual-level adjustment, perhaps because these studies were often much smaller and were conducted in single health care systems with, as a result, more homogeneous patient and hospital samples.

Some of the observed differences in rates of mortality or discharge to hospice between Black and White patients were attributable to the overrepresentation or underrepresentation in Black populations of social, demographic, and clinical risk factors known to be associated with these outcomes. However, focusing on outcomes adjusted for factors that may themselves be products of racism can obscure the very factors we want to call out.

Second, this study is distinguished by its sequential analytic adjustment from raw observed racial differences in the mortality rate. This sequential adjustment helps illustrate the social and demographic factors that contribute to the association of race and clinical outcomes from COVID-19.

Black patients hospitalized with COVID-19 had 11% higher odds of mortality than White patients—even after adjustment for those individual-level characteristics. The third and main contribution of this study is that those differences were largely explainable by the hospitals to which Black and White patients were admitted. Because COVID-19 mortality differences were also associated with hospitals within states, and Black patients were distributed differently than White patients across states, it is possible that racial mortality differences were created at the state level rather than the hospital level. This important distinction deserves further research. If the operative level is the hospital, a potential approach would be to encourage admissions to high-quality hospitals and also target quality improvement resources to those hospitals with low quality. If the operative level is the state, solutions require broader approaches incorporating resources and quality improvement initiatives to all hospitals within a state.

The compelling literature^6,7,8,9,10^ from other clinical contexts that racial disparities in quality of care are often attributable to hospital-level segregation makes it more plausible that the racial mortality differences we saw for COVID-19 also reflect differences in hospitals rather than differences in the states in which they were nested. This interpretation suggests that the additional mortality faced by Black patients might be eliminated if Black patients received care in the same hospitals and in the same distribution as White patients. This is a novel finding, and it adds to the evidence of structural factors that disproportionately burden the health of Black people in the US.

Why were Black patients admitted to different hospitals than White patients? Racial residential segregation surely contributed to this process because patients tend to go to nearby hospitals,^16,17,18^ and hospitals located in disadvantaged neighborhoods may have worse finances and provide care of lower quality as a result of differences in payer mix or community resources.^19^ Black and White patients may also differ in how they are exposed to or respond to referral patterns that may ultimately direct them to one hospital or another.^20^

### Strengths and Limitations

A strength of this study is that it represents a geographically and sociodemographically diverse group of 44 217 patients and 1188 hospitals in the US, allowing confidence in the estimation of individual-level patient factors associated with mortality. The size and diversity allowed for comparison of many alternative models that helped expose how racial differences in mortality were confounded by tangled social, demographic, and clinical factors.

This study has limitations. First, the analysis was restricted to Medicare Advantage beneficiaries from a single US insurer, a group that is largely older than 65 years and unevenly distributed across the US geographically and demographically. Nevertheless, this study reflects, to our knowledge, the largest and most comprehensive sample of US hospitals to date. Second, we were unable to measure out-of-hospital mortality rates for individuals with COVID-19. However, it seems plausible that most COVID-19 deaths among hospitalized patients occurred in the hospital, which was observable in our data. We used the composite outcome of death or discharge to hospice within 30 days to more comprehensively reflect mortality at any site. Third, we did not measure morbidity and disability outcomes among survivors, which may be meaningful.

## Conclusions

The findings of this cohort study suggest that differences in the mortality outcomes of Black and White patients were partly explained by adjustment for social, demographic, and clinical factors also associated with race. However, many of these factors are associated with past and ongoing unfairness, and, even after adjustment for those factors, racial differences in the mortality of patients hospitalized with COVID-19 remained. Those differences are almost entirely explained by the hospitals to which Black and White patients were admitted. Addressing hospital segregation and the uneven resourcing and quality of hospitals that provide care to a disproportionate number of Black patients may help address racial differences in the mortality rate.

## Notes

Supplement. **eAppendix 1.** Supplemental Methods **eFigure 1.** Forest Plot of Estimated Odds Ratios of 30-Day Mortality or Transfer to Hospice Adjusted for Race **eFigure 2.** Forest Plot of Estimated Odds Ratios of 30-day Mortality or Transfer to Hospice Adjusted for Race and Demographic Variables **eFigure 3.** Forest Plot of Estimated Odds Ratios of 30-Day Mortality or Transfer to Hospice Adjusted for Race, Demographic Variables, and Member-Level Income Measured by Zip Code **eFigure 4.** Forest Plot of Estimated Odds Ratios of 30-Day Mortality or Transfer to Hospice Adjusted for Race, Demographic Variables, Member-Level Income (by Zip Code), and Clinical Comorbidities Along With Nursing Home Admission Status **eFigure 5.** Forest Plot of Estimated Odds Ratios of 30-Day Mortality or Transfer to Hospice Adjusted for Race, Demographic Variables, Member-Level Income (by Zip Code), Clinical Comorbidities Along With Nursing Home Admission Status, and Time Passed Between January 1, 2020, and Patient’s Hospital Admission Date **eFigure 6.** Forest Plot of Estimated Odds Ratios of 30-Day Mortality or Transfer to Hospice Adjusted For Race, Demographic Variables, Member-Level Income (by Zip Code), Clinical Comorbidities Along With Nursing Home Admission Status, Time Differences, and Census Region in Which Hospitals Are Located **eFigure 7.** Forest Plot of Estimated Odds Ratios of 30-Day Mortality or Transfer to Hospice Adjusted for Race, Demographic Variables, Member-Level Income (by Zip Code), Clinical Comorbidities Along With Nursing Home Admission Status, Time Differences, and 1,188 Hospitals **eFigure 8.** Forest Plot of Estimated Odds Ratios of 30-Day Mortality or Transfer to Hospice Adjusted for Race, Demographic Variables, Member-Level Income (by Zip Code), Clinical Comorbidities Along With Nursing Home Admission Status, Time Differences, and States at Which Hospitals Are Located **eFigure 9.** Forest Plot of Estimated Odds Ratios of 30-Day Mortality or Transfer to Hospice Adjusted Only for Fixed Effects of 1,188 Hospitals **eFigure 10.** Differences of Adjusted Odds Ratios Associated With Race Effect Based on 500 Bootstrap Samples Between Nested Models **eFigure 11.** Distribution of Predicted Probabilities Had Each Patient, Hypothetically, Been Black and White Based on Model-C (Top-Left), Model-D (Top-Right), Model-E (Middle-Left), Model-F (Middle-Right), and Model-G (Bottom) **eFigure 12.** Derivation of the Analytical Dataset **eTable 1.** Goodness of Fit Measures for Nested Models **eTable 2.** *ICD-10* Codes Used in the Analyses **eAppendix 2.** An Illustration of Estimating Mortality Differences in Black and White Patients Based on a Simulated Dataset
eReferences.
