# Supplementary material for: Patient and Hospital Factors Associated With Differences in Mortality Rates Among Black and White US Medicare Beneficiaries Hospitalized With COVID-19 Infection
Source: JAMA Netw Open. 2021 Jun 17;4(6):e2112842. doi: 10.1001/jamanetworkopen.2021.12842 (PMC11849740; doi:10.1001/jamanetworkopen.2021.12842)
Supplement: Supplement. — eAppendix 1. Supplemental Methods eFigure 1. Forest Plot of Estimated Odds Ratios of 30-Day Mortality or Transfer to Hospice Adjusted for Race eFigure 2. Forest Plot of Estimated Odds Ratios of 30-day Mortality or Transfer to Hospice Adjusted for Race and Demographic Variables eFigure 3. Forest Plot of Estimated Odds Ratios of 30-Day Mortality or Transfer to Hospice Adjusted for Race, Demographic Variables, and Member-Level Income Measured by Zip Code eFigure 4. Forest Plot of Estimated Odds Ratios of 30-Day Mortality or Transfer to Hospice Adjusted for Race, Demographic Variables, Member-Level Income (by Zip Code), and Clinical Comorbidities Along With Nursing Home Admission Status eFigure 5. Forest Plot of Estimated Odds Ratios of 30-Day Mortality or Transfer to Hospice Adjusted for Race, Demographic Variables, Member-Level Income (by Zip Code), Clinical Comorbidities Along With Nursing Home Admission Status, and Time Passed Between January 1, 2020, and Patient’s Hospital Admission Date eFigure 6. Forest Plot of Estimated Odds Ratios of 30-Day Mortality or Transfer to Hospice Adjusted For Race, Demographic Variables, Member-Level Income (by Zip Code), Clinical Comorbidities Along With Nursing Home Admission Status, Time Differences, and Census Region in Which Hospitals Are Located eFigure 7. Forest Plot of Estimated Odds Ratios of 30-Day Mortality or Transfer to Hospice Adjusted for Race, Demographic Variables, Member-Level Income (by Zip Code), Clinical Comorbidities Along With Nursing Home Admission Status, Time Differences, and 1,188 Hospitals eFigure 8. Forest Plot of Estimated Odds Ratios of 30-Day Mortality or Transfer to Hospice Adjusted for Race, Demographic Variables, Member-Level Income (by Zip Code), Clinical Comorbidities Along With Nursing Home Admission Status, Time Differences, and States at Which Hospitals Are Located eFigure 9. Forest Plot of Estimated Odds Ratios of 30-Day Mortality or Transfer to Hospice Adjusted Only for Fixed Effects of 1,188 [file jamanetwopen-e2112842-s001.pdf]

# Supplementary Online Content

Asch DA, Islam MN, Sheils NE, et al. Patient and hospital factors associated with differences in mortality rates among Black and White US Medicare beneficiaries hospitalized with COVID-19 infection. *JAMA Netw Open*. 2021;4(6):e2112842.
doi:10.1001/jamanetworkopen.2021.12842

**eAppendix 1.** Supplemental Methods

**eFigure 1.** Forest Plot of Estimated Odds Ratios of 30-Day Mortality or Transfer to Hospice Adjusted for Race

**eFigure 2.** Forest Plot of Estimated Odds Ratios of 30-day Mortality or Transfer to Hospice Adjusted for Race and Demographic Variables

**eFigure 3.** Forest Plot of Estimated Odds Ratios of 30-Day Mortality or Transfer to Hospice Adjusted for Race, Demographic Variables, and Member-Level Income Measured by Zip Code

**eFigure 4.** Forest Plot of Estimated Odds Ratios of 30-Day Mortality or Transfer to Hospice Adjusted for Race, Demographic Variables, Member-Level Income (by Zip Code), and Clinical Comorbidities Along With Nursing Home Admission Status

**eFigure 5.** Forest Plot of Estimated Odds Ratios of 30-Day Mortality or Transfer to Hospice Adjusted for Race, Demographic Variables, Member-Level Income (by Zip Code), Clinical Comorbidities Along With Nursing Home Admission Status, and Time Passed Between January 1, 2020, and Patient's Hospital Admission Date

**eFigure 6.** Forest Plot of Estimated Odds Ratios of 30-Day Mortality or Transfer to Hospice Adjusted For Race, Demographic Variables, Member-Level Income (by Zip Code), Clinical Comorbidities Along With Nursing Home Admission Status, Time Differences, and Census Region in Which Hospitals Are Located

**eFigure 7.** Forest Plot of Estimated Odds Ratios of 30-Day Mortality or Transfer to Hospice Adjusted for Race, Demographic Variables, Member-Level Income (by Zip Code), Clinical Comorbidities Along With Nursing Home Admission Status, Time Differences, and 1,188 Hospitals

**eFigure 8.** Forest Plot of Estimated Odds Ratios of 30-Day Mortality or Transfer to Hospice Adjusted for Race, Demographic Variables, Member-Level Income (by Zip Code), Clinical Comorbidities Along With Nursing Home Admission Status, Time Differences, and States at Which Hospitals Are Located

**eFigure 9.** Forest Plot of Estimated Odds Ratios of 30-Day Mortality or Transfer to Hospice Adjusted Only for Fixed Effects of 1,188 Hospitals

**eFigure 10.** Differences of Adjusted Odds Ratios Associated With Race Effect Based on 500 Bootstrap Samples Between Nested Models.

**eFigure 11.** Distribution of Predicted Probabilities Had Each Patient, Hypothetically, Been Black and White Based on Model-C (Top-Left), Model-D (Top-Right), Model-E (Middle-Left), Model-F (Middle-Right), and Model-G (Bottom)

**eFigure 12.** Derivation of the Analytical Dataset

**eTable 1.** Goodness of Fit Measures for Nested Models

**eTable 2.** *ICD-10* Codes Used in the Analyses

**eAppendix 2.** An Illustration of Estimating Mortality Differences in Black and White Patients Based on a Simulated Dataset

**eReferences.**

This supplementary material has been provided by the authors to give readers additional information about their work.

© 2021 Asch DA et al. *JAMA Network Open.*

## **eAppendix 1.** Supplemental Methods

### Data Sources

We used administrative claims for Medicare Advantage and commercially insured enrollees in a research database from a single large health insurance provider in the United States.

We also used an inpatient COVID-19 dataset which included a daily updated record of COVID-19 inpatient admissions for all insurance enrollees with claims information, representing those admitted to a hospital with a primary or secondary diagnosis of COVID-19 (eTable 2), along with their current disposition (admitted, discharged, transferred, or expired).

### A. Technical details for statistical analyses

Let $i$ denote the hospital and j the COVID patients such that $\mathrm{i} = 1, \ldots, \mathrm{I}$ and $\mathrm{j} = 1, \ldots, n_i$, where $I$ is the total number of hospitals and $n_i$ is the total number of patients with COVID-related hospitalization at hospital i. Let $N = \sum_{i=1}^{I} n_i$ be the total number of patients. Let $Y_{ij}$ refer to the status of the $j$-th patient taking value of 1 if a patient is deceased or transferred to hospice within 30-days of hospital admission, or 0 otherwise. Denote by $U_{ijm}$ the m-th demographic variable and $X_{ijl}$ the l-th comorbidity status associated with the $j$-th subject admitted in the i-th hospital. In addition, we consider time variables, denoted by $T_{ijr}$, quantifying the days between January 1st and the patient's admission dates. These time differences are categorized into R discrete bins featuring different pandemic phases. In the following sections, we describe the statistical models and present the results. Define by $\boldsymbol{U_{ij}}$, $\boldsymbol{X_{ij}}$, and $\boldsymbol{T_{ij}}$ as $M, L$, and $R$ dimensional vector of covariates for the $ij$-th instance.

### A.1 Description of association models

We cast the following generalized linear model to characterize the association between covariates and binary outcome with fixed hospital effects quantifying between sites variation in our sample.

$$logit\ \Pr(Y_{ij} = 1) = \alpha + \sum_{m=1}^{M} U_{ijm}\delta_m + \sum_{l=1}^{L} X_{ijl}\beta_l + \sum_{r=1}^{R} T_{ijr}\tau_r + \gamma_i.$$

Here $\alpha$, $\delta_m, \beta_l, \tau_r$, and $\gamma_i$ correspond to the fixed parameters associated with the intercept, demographic, comorbidities, pandemic phases, and hospital attributes, respectively. While the above model is viewed as a full (saturated) model, we add-and-estimate the effect of risk factors sequentially resulting in a series of reduced and nested models; one of the objectives is to assess whether the racial segregation can be explained by adding such variables sequentially.

We fit the GLM[1] using the logit link function and estimate the parameters by the iteratively re-weighted least square (IRLS) algorithm; the model is implemented using the $stats$ R-package. eFigures 1-8 report the estimated adjusted odds ratios of various risk factors along with 95% confidence intervals and $p$-values based on Wald Z-statistic for testing the null effects; distributional results for GLMs are based on large sample approximations. Inverse variance weighted means for logit are calculated for each nested model had each patient (hypothetically) been Black and white; inverse-link transformation is used to obtain predicted mean values in probability scale. We assess the numerical performances of nested models by McFadden, Craig-Uhler's pseudo $R$-squared values, Somer's $D_{xy}$, and $C$-statistic. In general, as we add more clinically relevant covariates, the model quantifies more variation in response reflected in better predictive performance. Collinearity between covariates are assessed by generalized variance inflation factor (GVIF); where all values are less than 2.00. We compare nested GLMs to test the appropriateness of adding additional parameters using asymptotic likelihood ratio test (LRT); see $p$-

© 2021 Asch DA et al. *JAMA Network Open.*

values in eTable 1. For the fixed effect model with hospital effects, inference based on profiling approach treating hospital effect as a nuisance parameter is implemented.[2] Though results based on profiling method is not reported, both methods provide exact same results. The mean number of patients per site is approximately 37 with an inter quartile range (IQR) of 39.

## A.2 Inference about the difference between adjusted odds ratio of race effect

We use bootstrapping to test whether the adjusted odds ratio for Black patients estimated from different nested models are statistically different. The bootstrap steps are detailed as below. We repeat the steps for each comparison.

1) Sample $I$ hospitals with replacements assuming hospitals are independent
2) Fit multiple GLMs with respect to competing models using all patients obtained from the bootstrap sample. If some hospitals appear more than once in a bootstrapped sample, we treat them as distinct so that we have $I$ hospital effects to adjust.
3) Estimate fixed regression coefficients $\alpha$, $\delta_m$, $\beta_l$, $\tau_r$, and $\gamma_i$.
4) Estimate adjusted odds ratio for race effects for each model of comparison and calculate the difference.
5) Repeat (1)-(4) 500 times.
6) Estimate bootstrap standard error of difference. Use Wald test statistic to obtain $p$-values (at 5% level of significance) for testing of no difference between estimated aORs associated with competing models.
7) Estimate the bootstrapped based 95% confidence intervals (CIs) based on 2.5$^{th}$ and 97.5$^{th}$ percentiles of differences.

Reported in eFigure 10 are the differences of adjusted odds ratios (aORs) for an underlying race effect between nested models based on 500 bootstrap samples. The corresponding mean differences and 95% CIs based on percentile bootstrap are (0.15; 95% CI: 0.10-0.21), (0.09; 95% CI: 0.04-0.14), (0.08; 95% CI: 0.03-0.13), for “D vs G,” “E vs G,” “F vs G,” respectively. The corresponding $p$-values for testing no differences based on Wald test statistic are <0.0001, 0.0003, 0.0007, respectively. We conjecture that there are significant differences between the estimated odds ratio for Black with the smallest absolute difference between model-H and model-I.

## A.3 Computation of risk standardized event rates

We compute risk standardized event rates (RSERs) where the main idea is to fit a generalized linear mixed model (GLMM),[3] coined as “hierarchical model,” with random hospital effects, $\gamma_{i0}$, that are assumed to follow Gaussian distribution with mean $\alpha + \log(1 + bed_i)$ and unknown variance; see Silber, et al.[4,5,6]

$$logit \Pr(Y_{ij} = 1) = \alpha + \sum_{m=1}^{M} U_{ijm}\delta_m + \sum_{l=1}^{L} X_{ijl}\beta_l + \sum_{r=1}^{R} T_{ijr}\tau_r + \kappa log(1 + bed_i) + \gamma_{i0}$$

Here $\alpha$, $\delta_m$, $\beta_l$, $\tau_r$, and $\kappa$ correspond to the fixed parameters associated with the intercept, demographic, comorbidities, pandemic phases, and hospital attributes, respectively.

Denote by $i*$ a distinct hospital from hospital $i$, define $p_{i*j} = E\,(Y_{i*j} = 1 \big| \boldsymbol{X_{ij}}, \boldsymbol{U_{ij}}, \boldsymbol{T_{ij}}, bed_{i*}, \gamma_{i*0}) = 1/\{1 + exp(\text{-}\alpha - \sum_{m=1}^{M} U_{ijm}\delta_m - \sum_{l=1}^{L} X_{ijl}\beta_l - \sum_{r=1}^{R} T_{ijr}\tau_r - \kappa log(1 + bed_{i*}) - \gamma_{i*0})\}$, the

© 2021 Asch DA et al. *JAMA Network Open.*

probability of experiencing the event for the j-th patient admitted in the i-th hospital. Next, directly standardized (DS) event rates for the $i$-th hospital are computed as

$$s_{i,DS} = \sum_{i*=1}^{I} \sum_{j=1}^{n_{i*}} p_{i*j} \ / \ N.$$

Using a similar intuition, we calculate risk standardized mortality rates (RSMRs), though not reported, where the response is solely the inpatient mortality within 30 days of initial admission for COVID-19.

We fit the model assuming logit link function. The likelihood function of the GLMM is evaluated using adaptive Gauss-Hermite quadrature rule with eleven quadrature points; fixed and random effect parameters are estimated via optimizing the log likelihood function. The model is implemented using the $GLMMadaptive$ R-package with default control parameters.[7] Empirical best linear unbiased predictors (EBLUP) of random effects are obtained using a Bayesian formulation. The estimated variance of the random effects is 0.14 with 95% CI (0.10, 0.18). To check collinearity diagnostics, generalized variance inflation factors (< 2.00) are investigated for the fixed effects. The spearman correlation coefficient between GLM and GLMM adjusted for risk factors is 1.00 ($p$-value < 0.0001) indicating stability in parameter estimation. The corresponding measures for the goodness-of-fit are conditional $R$-squared value (0.14), $C$-statistic (0.68), and Somer's $D_{xy}$ (0.36), and $p$-value (0.1636) for Hosmer-Lemeshow test with ten groups.

## A.4 Simulation of counter-factual arguments

Let the number of Black and white patients denoted by $N_b$ and $N_w$. Using GLMM as descried above, we estimate the patient specific RSERs for each of $N_b$ patients who are hypothetically being treated at each of I hospitals resulting in an $(N_b \times I)$ matrix of RSERs. Let $\pi_0$ denote the observed (weighted) event rate for $N_b$ Black patients. To entertain the counterfactual argument of Black patients going to the hospitals where white patients typically are admitted, we randomly assign each Black patient to a hospital using a probability distribution. We apply multinomial distribution where each patient can be assigned to one of I hospitals and the probability of assignment is dictated by the corresponding proportion of whites distributed in each hospital. Consider I mutually exclusive choices with the corresponding probabilities of $p_1,\ p_2,\ \ldots,\ \text{and}\ p_I$,. We simulate data as below -

1) Do for each simulation
   a) Estimate the cumulative probabilities and divide the (0, 1) interval into I subintervals with each being equal in length to the probabilities of the I categories.
   b) Generate $N_b$ independent pseudo-random numbers using uniform distribution between 0 and 1 to determine in which of the $I$ intervals they belong to.
   c) Need to ensure there is at-least one Black patient in each of the I categories to be consistent with the derivation of the original analytical dataset. It is possible that in some random assignments, there are categories with no Black patients; this could be due to the small proportions of white patients admitted to some hospitals. Say, there are $I_0$ such intervals with zero Black patients. In this situation, $I_0$ Black patients are randomly selected without replacement from the pool of categories each having more than ten Black patients and transferred to $I_0$ categories; note the sum of the counts of the categories should be equal to $N_b$.
   d) Obtain patients' pseudo hospital ID based on the random assignments and concatenate them in an $(N_b \times 1)$-dimensional vector. Take average of patient level RSERs and denote it by $\pi_b$.
2) Repeat the process 1000 times.

© 2021 Asch DA et al. *JAMA Network Open.*

3) Calculate the mean of $\pi_b$; $b = 1, \ldots, 1000$. Obtain 95% confidence intervals via 2.5$^{th}$ and 97.5$^{th}$ percentiles of differences between $\pi_0$ and mean estimates $\pi_b$.

© 2021 Asch DA et al. *JAMA Network Open.*

## B. Analytical results

In this section, we report the pertinent results associated with the models described in Section-A and in the manuscript.

**eFigure 1.** Forest plot of estimated odds ratios of 30-day mortality or transfer to hospice adjusted for race.

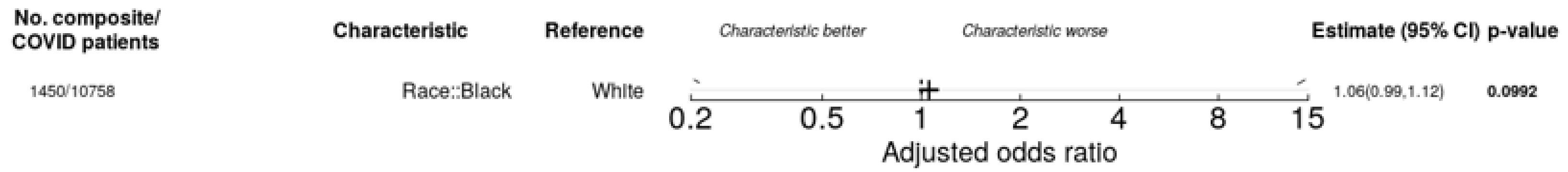


**eFigure 2.** Forest plot of estimated odds ratios of 30-day mortality or transfer to hospice adjusted for race and demographic variables.

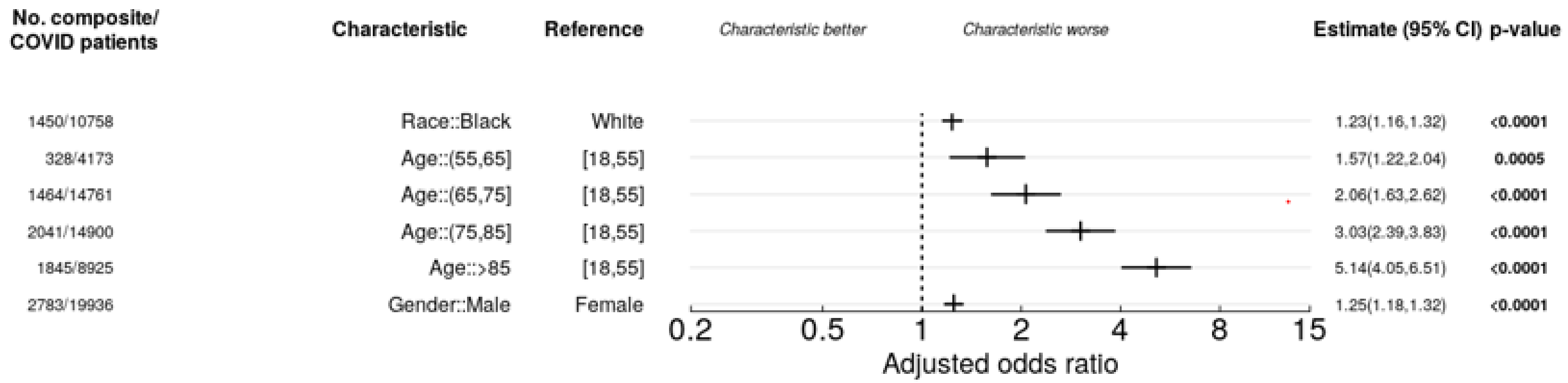


© 2021 Asch DA et al. *JAMA Network Open*

**eFigure3.** Forest plot of estimated odds ratios of 30-day mortality or transfer to hospice adjusted for race, demographic variables, and member-level income measured by zip code.

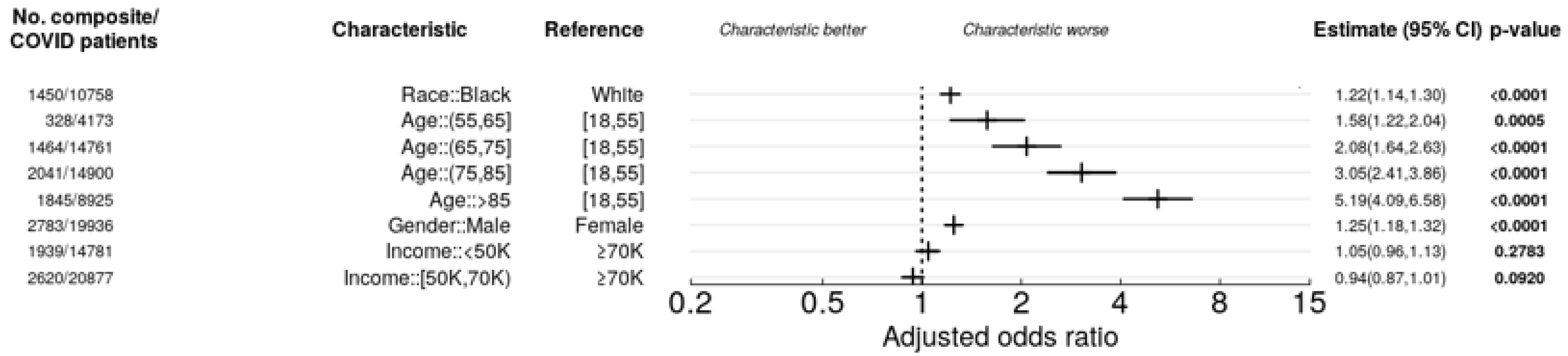


© 2021 Asch DA et al. *JAMA Network Open*

**eFigure 4.** Forest plot of estimated odds ratios of 30-day mortality or transfer to hospice adjusted for race, demographic variables, member-level income (by zip code), and clinical comorbidities along with nursing home admission status.

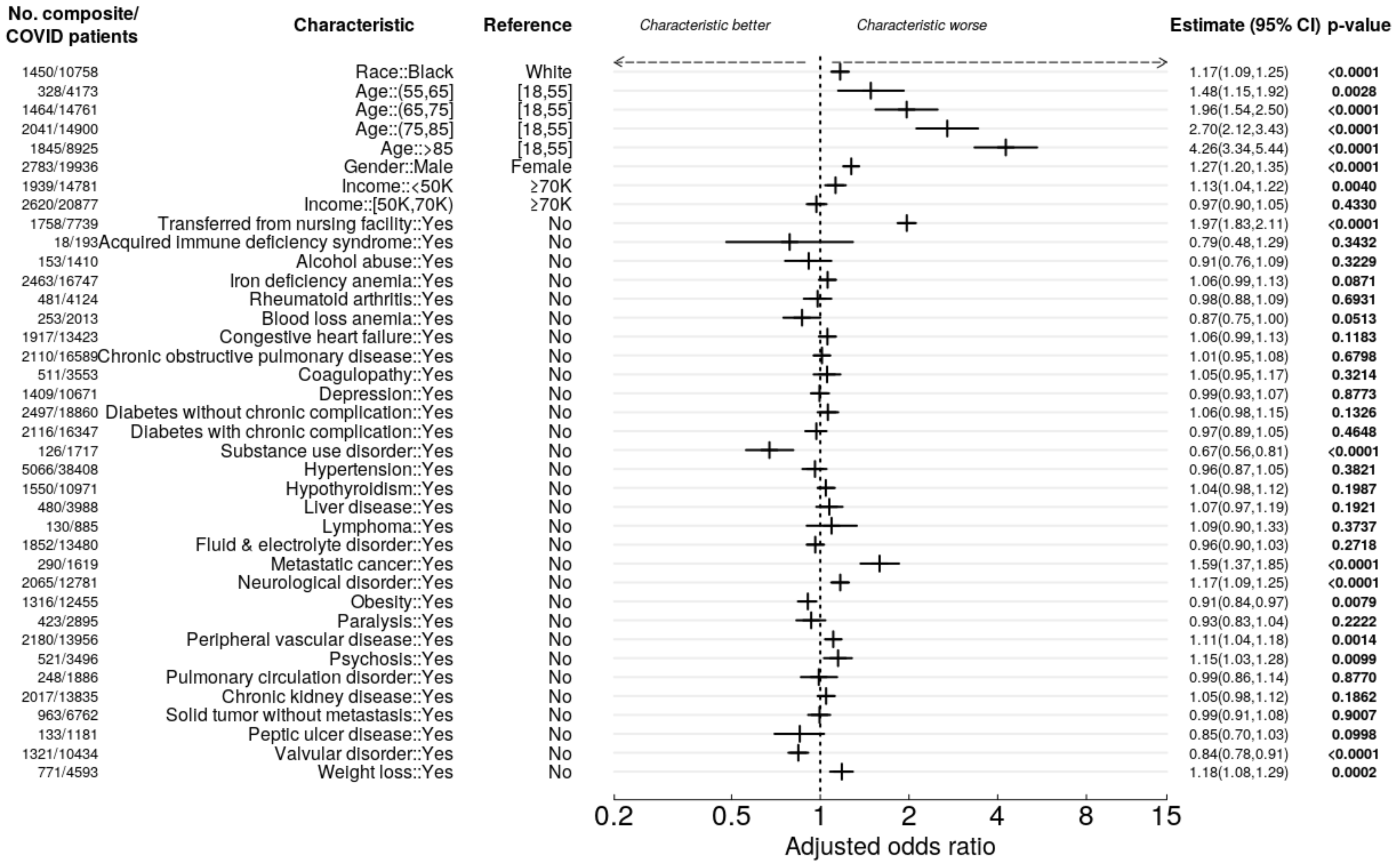


© 2021 Asch DA et al. *JAMA Network Open*

**eFigure 5.** Forest plot of estimated odds ratios of 30-day mortality or transfer to hospice adjusted for race, demographic variables, member-level income (by zip code), clinical comorbidities along with nursing home admission status, and time passed between January 1, 2020 and patient's hospital admission date.

| No. composite/ COVID patients | Characteristic | Reference | Estimate (95% CI) | p-value |
|---|---|---|---|---|
| 1450/10758 | Race::Black | White | 1.11(1.03,1.19) | **0.0047** |
| 328/4173 | Age::(55,65] | [18,55] | 1.46(1.12,1.89) | **0.0046** |
| 1464/14761 | Age::(65,75] | [18,55] | 1.91(1.50,2.43) | **<0.0001** |
| 2041/14900 | Age::(75,85] | [18,55] | 2.64(2.08,3.36) | **<0.0001** |
| 1845/8925 | Age::>85 | [18,55] | 4.21(3.29,5.38) | **<0.0001** |
| 2783/19936 | Gender::Male | Female | 1.25(1.18,1.33) | **<0.0001** |
| 1939/14781 | Income::<50K | ≥70K | 1.24(1.14,1.35) | **<0.0001** |
| 2620/20877 | Income::[50K,70K) | ≥70K | 1.02(0.95,1.10) | **0.5627** |
| 1758/7739 | Transferred from nursing facility::Yes | No | 1.76(1.64,1.88) | **<0.0001** |
| 18/193 | Acquired immune deficiency syndrome::Yes | No | 0.77(0.47,1.27) | **0.3100** |
| 153/1410 | Alcohol abuse::Yes | No | 0.93(0.78,1.12) | **0.4467** |
| 2463/16747 | Iron deficiency anemia::Yes | No | 1.05(0.98,1.12) | **0.1912** |
| 481/4124 | Rheumatoid arthritis::Yes | No | 0.98(0.88,1.09) | **0.6824** |
| 253/2013 | Blood loss anemia::Yes | No | 0.88(0.77,1.02) | **0.0962** |
| 1917/13423 | Congestive heart failure::Yes | No | 1.06(0.98,1.13) | **0.1369** |
| 2110/16589 | Chronic obstructive pulmonary disease::Yes | No | 1.00(0.94,1.07) | **0.9923** |
| 511/3553 | Coagulopathy::Yes | No | 1.05(0.94,1.17) | **0.3635** |
| 1409/10671 | Depression::Yes | No | 1.00(0.93,1.07) | **0.9976** |
| 2497/18860 | Diabetes without chronic complication::Yes | No | 1.06(0.98,1.15) | **0.1385** |
| 2116/16347 | Diabetes with chronic complication::Yes | No | 0.97(0.89,1.05) | **0.4755** |
| 126/1717 | Substance use disorder::Yes | No | 0.67(0.55,0.81) | **<0.0001** |
| 5066/38408 | Hypertension::Yes | No | 0.97(0.88,1.06) | **0.4858** |
| 1550/10971 | Hypothyroidism::Yes | No | 1.04(0.97,1.11) | **0.2818** |
| 480/3988 | Liver disease::Yes | No | 1.06(0.96,1.18) | **0.2590** |
| 130/885 | Lymphoma::Yes | No | 1.09(0.90,1.33) | **0.3667** |
| 1852/13480 | Fluid & electrolyte disorder::Yes | No | 0.96(0.90,1.03) | **0.2712** |
| 290/1619 | Metastatic cancer::Yes | No | 1.60(1.38,1.86) | **<0.0001** |
| 2065/12781 | Neurological disorder::Yes | No | 1.17(1.09,1.25) | **<0.0001** |
| 1316/12455 | Obesity::Yes | No | 0.91(0.84,0.98) | **0.0082** |
| 423/2895 | Paralysis::Yes | No | 0.94(0.84,1.05) | **0.2712** |
| 2180/13956 | Peripheral vascular disease::Yes | No | 1.09(1.03,1.17) | **0.0049** |
| 521/3496 | Psychosis::Yes | No | 1.14(1.02,1.27) | **0.0176** |
| 248/1886 | Pulmonary circulation disorder::Yes | No | 0.98(0.85,1.13) | **0.7763** |
| 2017/13835 | Chronic kidney disease::Yes | No | 1.06(0.99,1.14) | **0.0714** |
| 963/6762 | Solid tumor without metastasis::Yes | No | 0.99(0.91,1.09) | **0.9044** |
| 133/1181 | Peptic ulcer disease::Yes | No | 0.86(0.71,1.05) | **0.1315** |
| 1321/10434 | Valvular disorder::Yes | No | 0.84(0.78,0.91) | **<0.0001** |
| 771/4593 | Weight loss::Yes | No | 1.18(1.08,1.29) | **0.0004** |
| 497/2290 | Days from January 1,2020::(0,90] | >180 | 2.23(1.99,2.50) | **<0.0001** |
| 1691/8161 | Days from January 1,2020::(90,120] | >180 | 1.89(1.75,2.04) | **<0.0001** |
| 1019/8560 | Days from January 1,2020::(120,150] | >180 | 1.03(0.95,1.12) | **0.5048** |
| 741/7926 | Days from January 1,2020::(150,180] | >180 | 0.84(0.77,0.92) | **0.0003** |

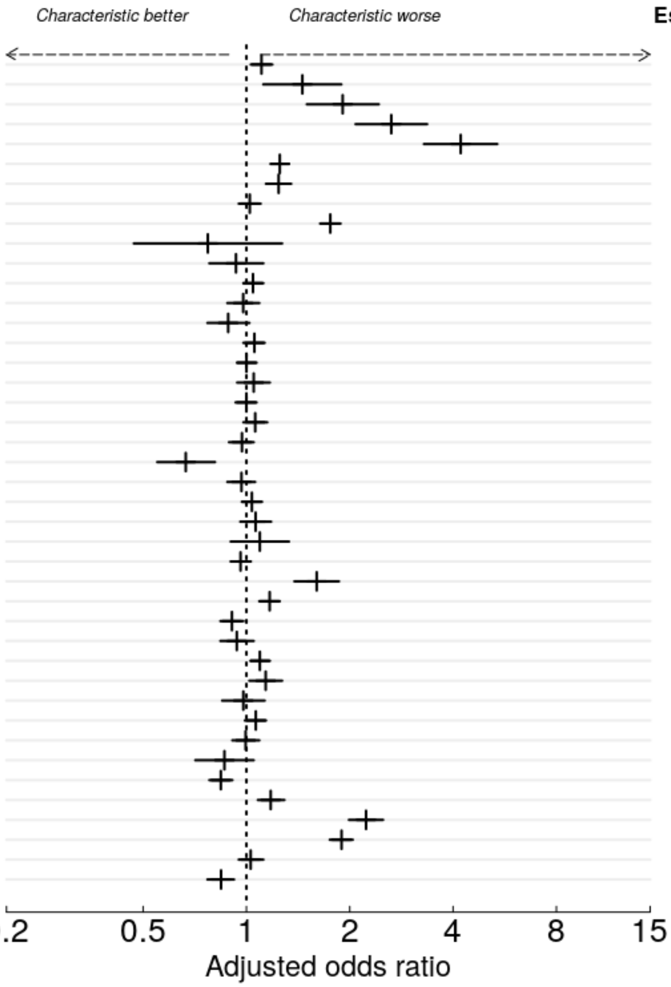


© 2021 Asch DA et al. *JAMA Network Open*

**eFigure 6.** Forest plot of estimated odds ratios of 30-day mortality or transfer to hospice adjusted for race, demographic variables, member-level income (by zip code), clinical comorbidities along with nursing home admission status, time differences, and census region in which hospitals are located.

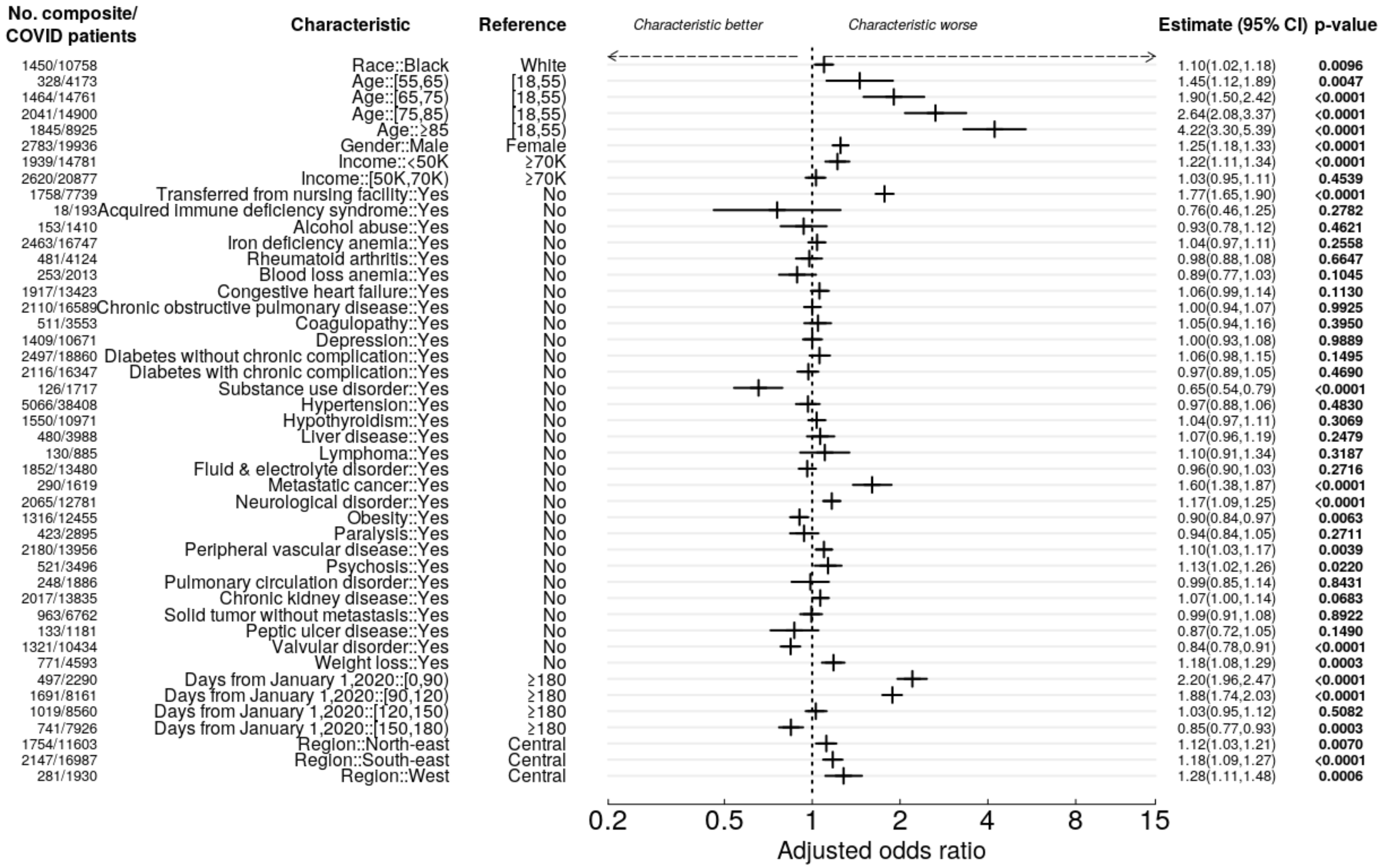


© 2021 Asch DA et al. *JAMA Network Open*

**eFigure 7.** Forest plot of estimated odds ratios of 30-day mortality or transfer to hospice adjusted for race, demographic variables, member-level income (by zip code), clinical comorbidities along with nursing home admission status, time differences, and 1,188 hospitals. We omit reporting each hospital's adjusted odds ratio for clarity.

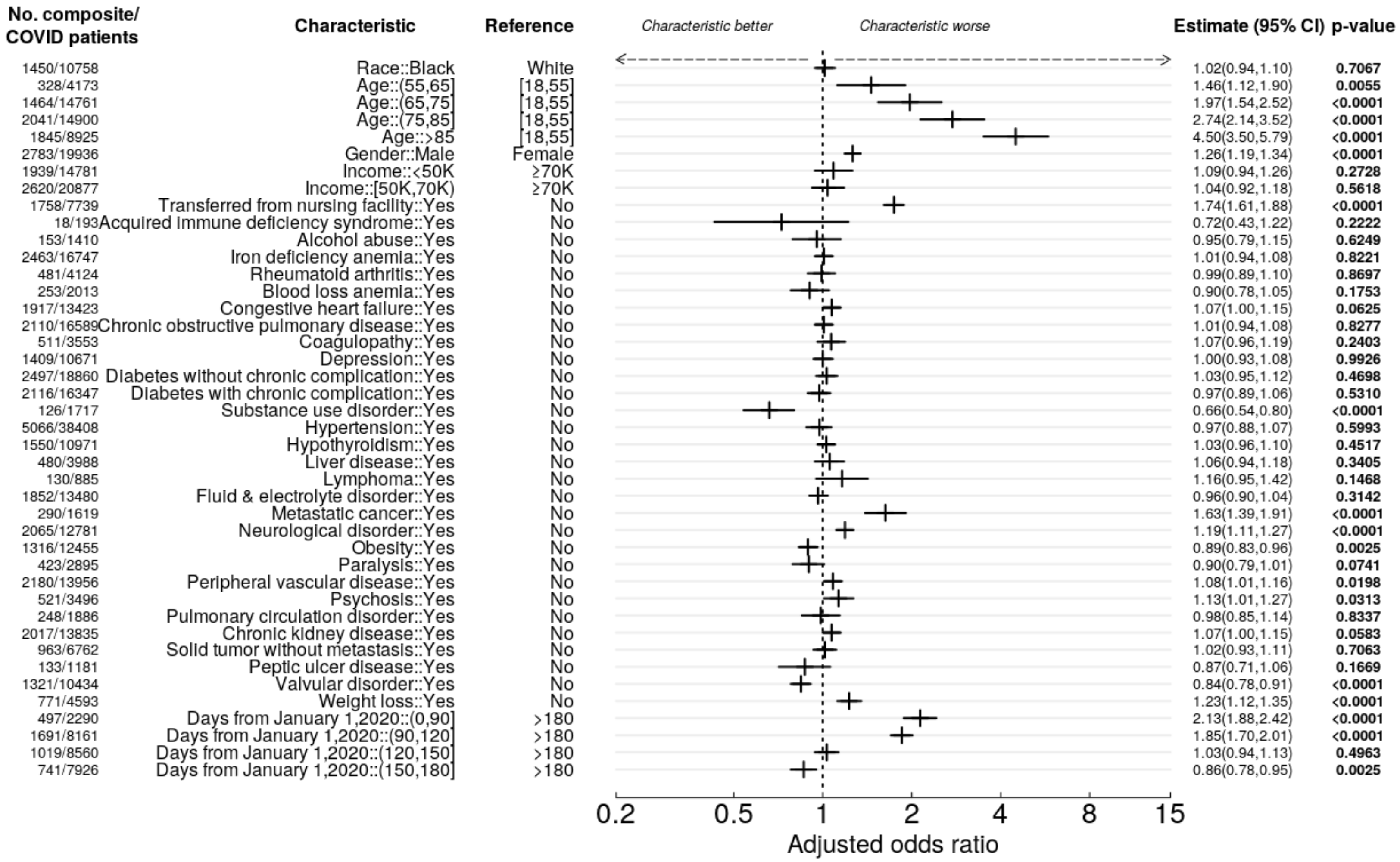


© 2021 Asch DA et al. *JAMA Network Open*

**eFigure 8.** Forest plot of estimated odds ratios of 30-day mortality or transfer to hospice adjusted for race, demographic variables, member-level income (by zip code), clinical comorbidities along with nursing home admission status, time differences, and states at which hospitals are located.

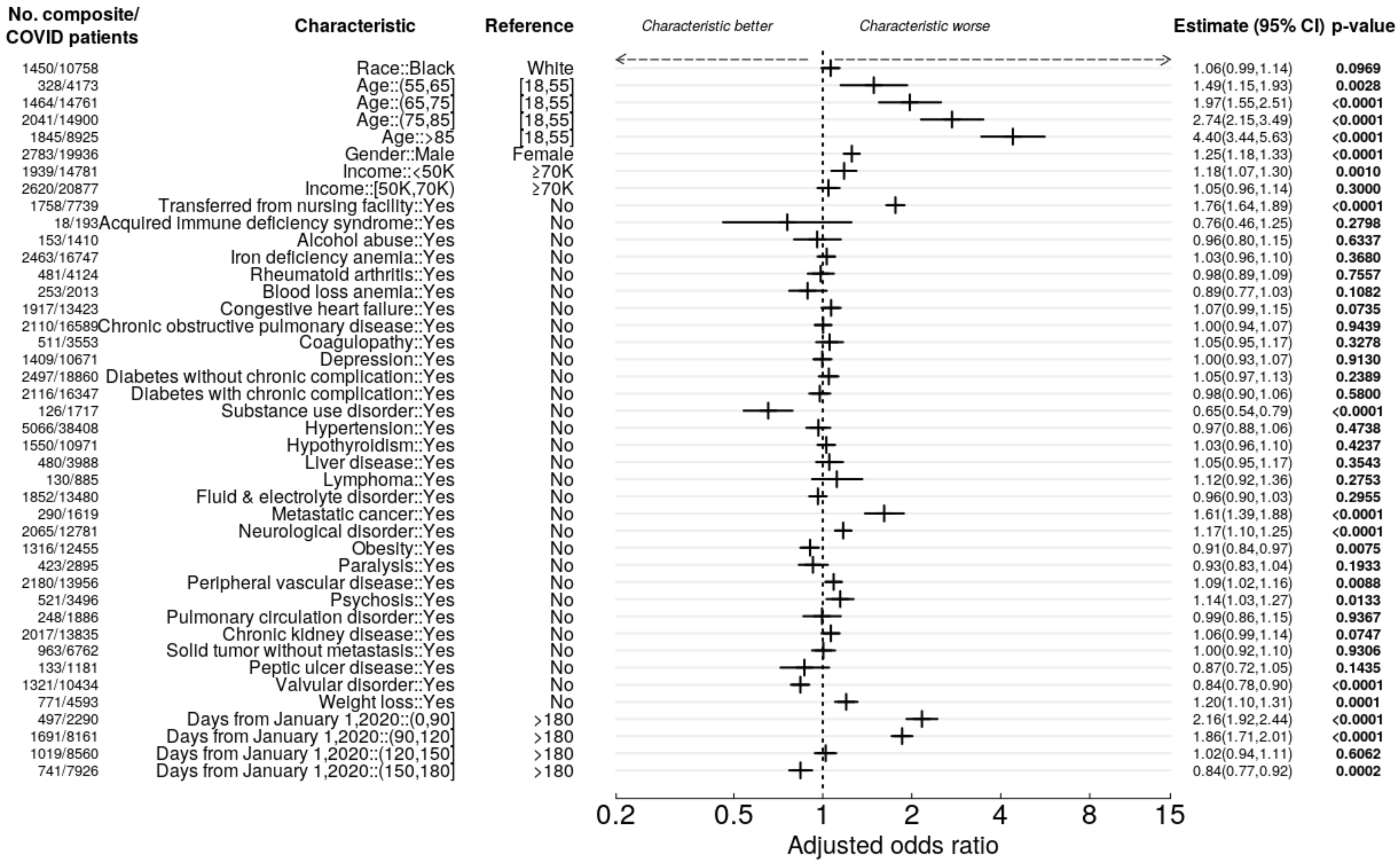


**eFigure 9.** Forest plot of estimated odds ratios of 30-day mortality or transfer to hospice adjusted only for fixed effects of 1,188 hospitals. We omit reporting each hospital's adjusted odds ratio for clarity.

© 2021 Asch DA et al. *JAMA Network Open*

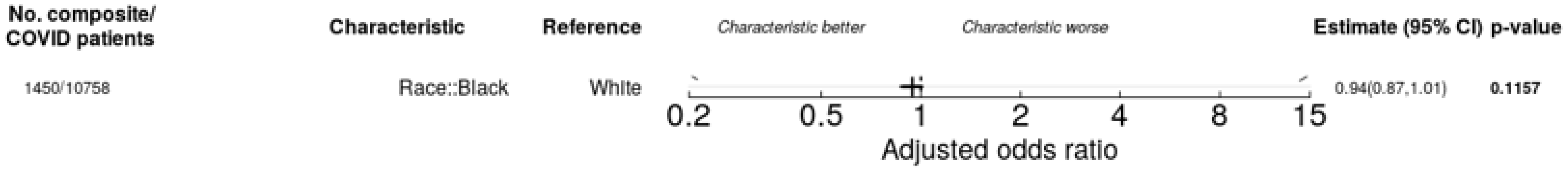


**eFigure 10.** Differences of adjusted odds ratios associated with race effect based on 500 bootstrap samples between nested models.

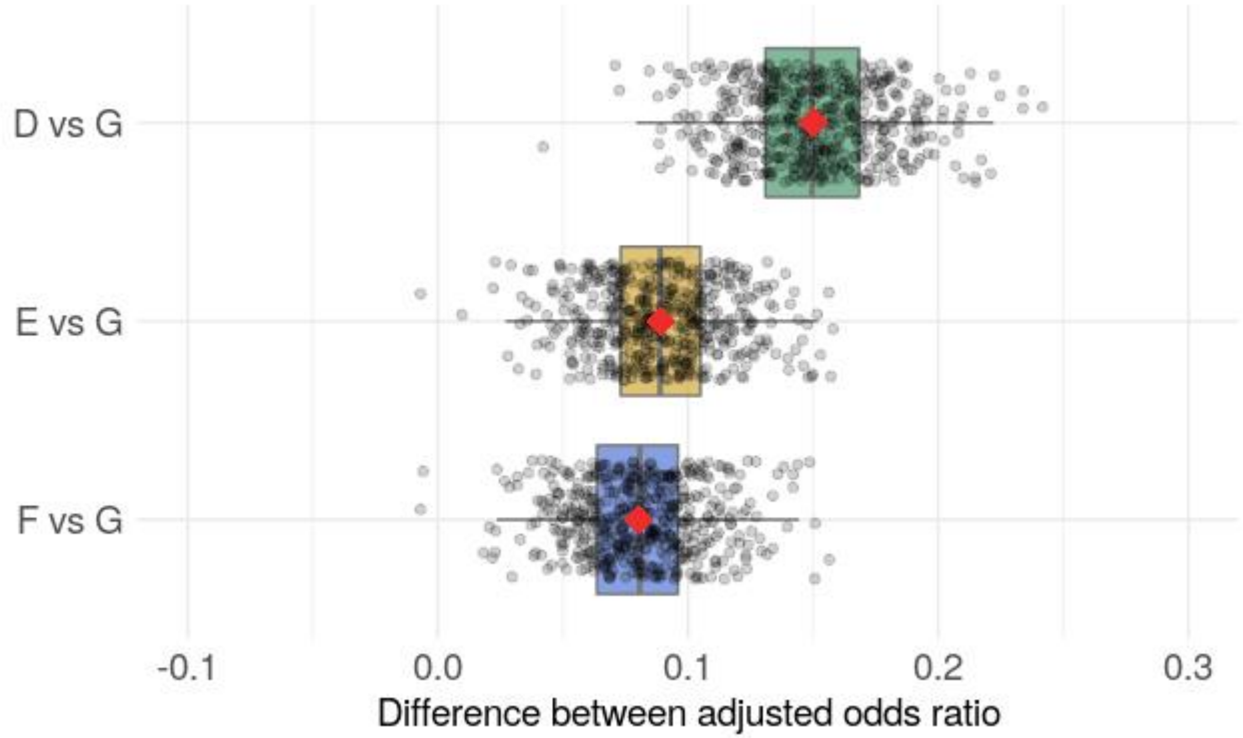


Red diamond = mean; gray line = median.

© 2021 Asch DA et al. *JAMA Network Open*

**eFigure 11.** Distribution of predicted probabilities had each patient, hypothetically, been Black and white based on model-C (top-left), model-D (top-right), model-E (middle-left), model-F (middle-right), and model-G (bottom). Smoothed histograms based on kernel density estimates are overlayed.

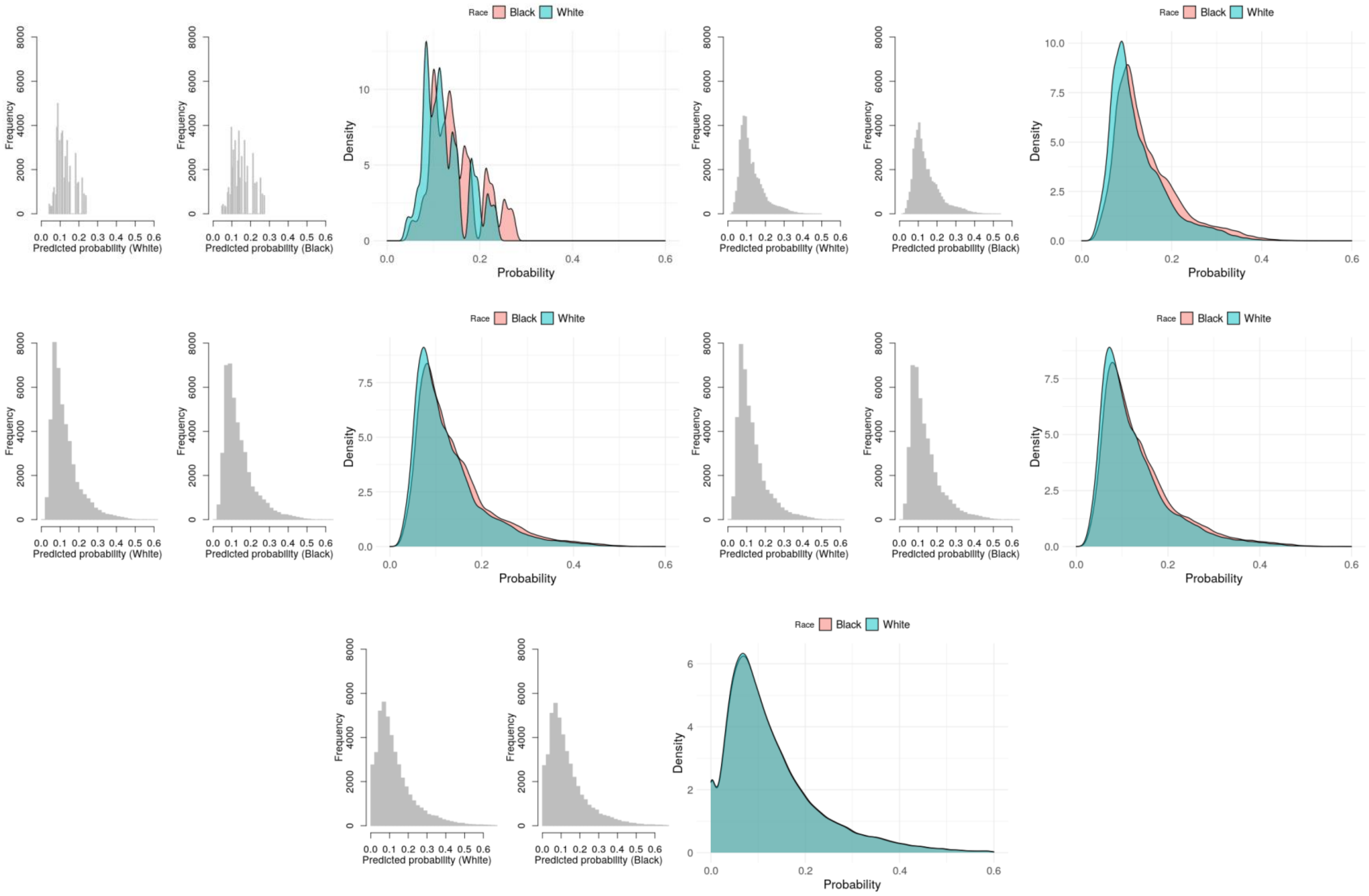


© 2021 Asch DA et al. *JAMA Network Open*

**eFigure 12.** Derivation of the analytical dataset.

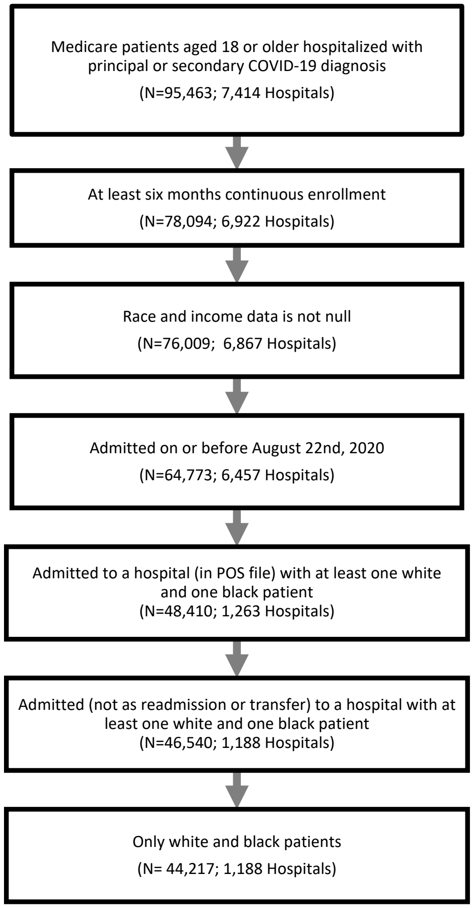


© 2021 Asch DA et al. *JAMA Network Open*

**eTable 1.** Goodness of fit measures for nested models.

| | Model | $C$-Statistic | Somer's $D_{xy}$ | Pseudo $R$-squared McFadden (Craig-Uhler) | $p$-value of LRT |
|---|---|---|---|---|---|
| **A** | Unadjusted 30-day inpatient mortality or referral to hospice | 0.50 | 0.01 | 0.00 (0.00) | NA |
| **B** | 30-day Inpatient mortality or referral to hospice adjusted for age and gender | 0.61 | 0.23 | 0.02 (0.04) | < 0.0001 A vs B |
| **C** | Same as above plus patient-level income | 0.62 | 0.23 | 0.03 (0.04) | 0.0036 B vs C |
| **D** | Same as above plus Elixhauser comorbidities and whether the patient was admitted from a nursing home | 0.66 | 0.32 | 0.05 (0.06) | < 0.0001 C vs D |
| **E** | Same as above plus days between admission and January 1, 2020 | 0.68 | 0.36 | 0.06 (0.09) | < 0.0001 D vs E |
| **F** | Same as above plus region | 0.68 | 0.36 | 0.06 (0.09) | < 0.0001 E vs F |
| **G** | 30-day inpatient mortality or referral to hospice adjusted for age, gender, income, comorbidities, days between admission and January 1, 2020, and hospital | 0.74 | 0.48 | 0.12 (0.16) | < 0.0001 E vs G |

© 2021 Asch DA et al. *JAMA Network Open*

**eTable 2.** *ICD-10* codes used in the analyses

## Inclusion Criteria

| Inclusion Criteria | ICD-10-CM codes |
|---|---|
| COVID-19 | U071, U072, B9729 |

## Elixhauser Comorbidity Indices

| Elixhauser Comorbidity Index | ICD-10-CM codes |
|---|---|
| Acquired immunodeficiency Syndrome | B20 |
| Alcohol Use Disorder | F1010, F1011, F10120, F10121, F10129, F1014, F10150, F10151, F10159, F10180, F10181, F10182, F10188, F1019, F1020, F1021, F10220, F10221, F10229, F10230, F10231, F10232, F10239, F1024, F10250, F10251, F10259, F1026, F1027, F10280, F10281, F10282, F10288, F1029, F10921, F1094, F10950, F10951, F10959, F1096, F1097, F10980, F10981, F10982, F10988, F1099 |
| Iron Deficiency Anemia | D501, D508, D509, D510, D511, D512, D513, D518, D519, D520, D521, D528, D529, D530, D531, D532, D538, D539, D630, D631, D638, D649 |
| Rheumatoid Arthritis | L900, L940, L941, L943, M0500, M05011, M05012, M05019, M05021, M05022, M05029, M05031, M05032, M05039, M05041, M05042, M05049, M05051, M05052, M05059, M05061, M05062, M05069, M05071, M05072, M05079, M0509, M0510, M05111, M05112, M05119, M05121, M05122, M05129, M05131, M05132, M05139, M05141, M05142, M05149, M05151, M05152, M05159, M05161, M05162, M05169, M05171, M05172, M05179, M0519, M0520, M05211, M05212, M05219, M05221, M05222, M05229, M05231, M05232, M05239, M05241, M05242, M05249, M05251, M05252, M05259, M05261, M05262, M05269, M05271, M05272, M05279, M0529, M0530, M05311, M05312, M05319, M05321, M05322, M05329, M05331, M05332, M05339, M05341, M05342, M05349, M05351, M05352, M05359, M05361, M05362, M05369, M05371, M05372, M05379, M0539, M0540, M05411, M05412, M05419, M05421, M05422, M05429, M05431, M05432, M05439, M05441, M05442, M05449, M05451, M05452, M05459, M05461, M05462, M05469, M05471, M05472, M05479, M0549, M0550, M05511, M05512, M05519, M05521, M05522, M05529, M05531, M05532, M05539, M05541, M05542, M05549, M05551, M05552, M05559, M05561, M05562, M05569, M05571, M05572, M05579, M0559, M0560, M05611, M05612, M05619, M05621, M05622, M05629, M05631, M05632, M05639, M05641, M05642, M05649, M05651, M05652, M05659, M05661, M05662, M05669, M05671, M05672, M05679, M0569, M0570, M05711, M05712, M05719, M05721, M05722, M05729, M05731, M05732, M05739, M05741, M05742, M05749, M05751, M05752, M05759, M05761, M05762, M05769, M05771, M05772, M05779, M0579, M0580, M05811, M05812, M05819, M05821, M05822, M05829, M05831, M05832, M05839, M05841, M05842, M05849, M05851, M05852, M05859, M05861, M05862, M05869, M05871, M05872, M05879, M0589, M059, M0600, M06011, M06012, M06019, M06021, M06022, M06029, M06031, M06032, M06039, M06041, M06042, M06049, M06051, M06052, M06059, M06061, M06062, M06069, M06071, M06072, M06079, M0608, M0609, M061, M0620, M06211, M06212, M06219, M06221, M06222, M06229, M06231, M06232, M06239, M06241, M06242, M06249, M06251, M06252, M06259, M06261, M06262, M06269, M06271, M06272, M06279, M0628, M0629, M0630, M06311, M06312, M06319, M06321, M06322, M06329, M06331, M06332, M06339, M06341, M06342, M06349, M06351, M06352, M06359, M06361, M06362, M06369, M06371, |

© 2021 Asch DA et al. *JAMA Network Open*

| | |
|---|---|
| | M06372, M06379, M0638, M0639, M064, M0680, M06811, M06812, M06819, M06821, M06822, M06829, M06831, M06832, M06839, M06841, M06842, M06849, M06851, M06852, M06859, M06861, M06862, M06869, M06871, M06872, M06879, M0688, M0689, M069, M0800, M08011, M08012, M08019, M08021, M08022, M08029, M08031, M08032, M08039, M08041, M08042, M08049, M08051, M08052, M08059, M08061, M08062, M08069, M08071, M08072, M08079, M0808, M0809, M081, M0820, M08211, M08212, M08219, M08221, M08222, M08229, M08231, M08232, M08239, M08241, M08242, M08249, M08251, M08252, M08259, M08261, M08262, M08269, M08271, M08272, M08279, M0828, M0829, M083, M0840, M08411, M08412, M08419, M08421, M08422, M08429, M08431, M08432, M08439, M08441, M08442, M08449, M08451, M08452, M08459, M08461, M08462, M08469, M08471, M08472, M08479, M0848, M0880, M08811, M08812, M08819, M08821, M08822, M08829, M08831, M08832, M08839, M08841, M08842, M08849, M08851, M08852, M08859, M08861, M08862, M08869, M08871, M08872, M08879, M0888, M0889, M0890, M08911, M08912, M08919, M08921, M08922, M08929, M08931, M08932, M08939, M08941, M08942, M08949, M08951, M08952, M08959, M08961, M08962, M08969, M08971, M08972, M08979, M0898, M0899, M1200, M12011, M12012, M12019, M12021, M12022, M12029, M12031, M12032, M12039, M12041, M12042, M12049, M12051, M12052, M12059, M12061, M12062, M12069, M12071, M12072, M12079, M1208, M1209, M320, M3210, M3211, M3212, M3213, M3214, M3215, M3219, M328, M329, M3300, M3301, M3302, M3303, M3309, M3310, M3311, M3312, M3313, M3319, M3320, M3321, M3322, M3329, M3390, M3391, M3392, M3393, M3399, M340, M341, M342, M3481, M3482, M3483, M3489, M349, M3500, M3501, M3502, M3503, M3504, M3509, M351, M353, M355, M358, M359, M360, M368, M450, M451, M452, M453, M454, M455, M456, M457, M458, M459, M4600, M4601, M4602, M4603, M4604, M4605, M4606, M4607, M4608, M4609, M461, M4650, M4651, M4652, M4653, M4654, M4655, M4656, M4657, M4658, M4659, M4680, M4681, M4682, M4683, M4684, M4685, M4686, M4687, M4688, M4689, M4690, M4691, M4692, M4693, M4694, M4695, M4696, M4697, M4698, M4699, M488X1, M488X2, M488X3, M488X4, M488X5, M488X6, M488X7, M488X8, M488X9, M4980, M4981, M4982, M4983, M4984, M4985, M4986, M4987, M4988, M4989 |
| Blood Loss Anemia | D500, O9081, O99011, O99012, O99013, O99019, O9902, O9903 |
| Congestive Heart Failure | I0981, I110, I130, I132, I501, I5020, I5021, I5022, I5023, I5030, I5031, I5032, I5033, I5040, I5041, I5042, I5043, I50810, I50811, I50812, I50813, I50814, I5082, I5083, I5084, I5089, I509 |
| Chronic Obstructive Pulmonary Disease | J40, J410, J411, J418, J42, J430, J431, J432, J438, J439, J440, J441, J449, J4520, J4521, J4522, J4530, J4531, J4532, J4540, J4541, J4542, J4550, J4551, J4552, J45901, J45902, J45909, J45990, J45991, J45998, J470, J471, J479, J60, J61, J620, J628, J630, J631, J632, J633, J634, J635, J636, J64, J660, J661, J662, J668, J670, J671, J672, J673, J674, J675, J676, J677, J678, J679, J684 |
| Coagulopathy | D65, D66, D67, D680, D681, D682, D68311, D68312, D68318, D6832, D684, D688, D689, D691, D693, D6941, D6942, D6949, D6951, D6959, D696, D7582, O99111, O99112, O99113, O99119, O9912, O9913 |
| Depression | F320, F321, F322, F323, F328, F3281, F3289, F329, F330, F331, F332, F333, F338, F339, F341, F4321 |
| Diabetes without Chronic Complications | E0800, E0801, E0810, E0811, E089, E0900, E0901, E0910, E0911, E099, E1010, E1011, E109, E1100, E1101, E1110, E1111, E119, E1300, E1301, E1310, E1311, E139, O24011, O24012, O24013, O24019, O2402, O2403, O24111, O24112, O24113, O24119, O2412, O2413, O24311, O24312, O24313, O24319, O2432, O2433, O24811, O24812, O24813, O24819, O2482, O2483, O24911, O24912, O24913, O24919, O2492, O2493 |

© 2021 Asch DA et al. *JAMA Network Open*

| | |
|---|---|
| Diabetes with Chronic Complications | E0821, E0822, E0829, E08311, E08319, E08321, E083211, E083212, E083213, E083219, E08329, E083291, E083292, E083293, E083299, E08331, E083311, E083312, E083313, E083319, E08339, E083391, E083392, E083393, E083399, E08341, E083411, E083412, E083413, E083419, E08349, E083491, E083492, E083493, E083499, E08351, E083511, E083512, E083513, E083519, E083521, E083522, E083523, E083529, E083531, E083532, E083533, E083539, E083541, E083542, E083543, E083549, E083551, E083552, E083553, E083559, E08359, E083591, E083592, E083593, E083599, E0836, E0837X1, E0837X2, E0837X3, E0837X9, E0839, E0840, E0841, E0842, E0843, E0844, E0849, E0851, E0852, E0859, E08610, E08618, E08620, E08621, E08622, E08628, E08630, E08638, E08641, E08649, E0865, E0869, E088, E0921, E0922, E0929, E09311, E09319, E09321, E093211, E093212, E093213, E093219, E09329, E093291, E093292, E093293, E093299, E09331, E093311, E093312, E093313, E093319, E09339, E093391, E093392, E093393, E093399, E09341, E093411, E093412, E093413, E093419, E09349, E093491, E093492, E093493, E093499, E09351, E093511, E093512, E093513, E093519, E093521, E093522, E093523, E093529, E093531, E093532, E093533, E093539, E093541, E093542, E093543, E093549, E093551, E093552, E093553, E093559, E09359, E093591, E093592, E093593, E093599, E0936, E0937X1, E0937X2, E0937X3, E0937X9, E0939, E0940, E0941, E0942, E0943, E0944, E0949, E0951, E0952, E0959, E09610, E09618, E09620, E09621, E09622, E09628, E09630, E09638, E09641, E09649, E0965, E0969, E098, E1021, E1022, E1029, E10311, E10319, E10321, E103211, E103212, E103213, E103219, E10329, E103291, E103292, E103293, E103299, E10331, E103311, E103312, E103313, E103319, E10339, E103391, E103392, E103393, E103399, E10341, E103411, E103412, E103413, E103419, E10349, E103491, E103492, E103493, E103499, E10351, E103511, E103512, E103513, E103519, E103521, E103522, E103523, E103529, E103531, E103532, E103533, E103539, E103541, E103542, E103543, E103549, E103551, E103552, E103553, E103559, E10359, E103591, E103592, E103593, E103599, E1036, E1037X1, E1037X2, E1037X3, E1037X9, E1039, E1040, E1041, E1042, E1043, E1044, E1049, E1051, E1052, E1059, E10610, E10618, E10620, E10621, E10622, E10628, E10630, E10638, E10641, E10649, E1065, E1069, E108, E1121, E1122, E1129, E11311, E11319, E11321, E113211, E113212, E113213, E113219, E11329, E113291, E113292, E113293, E113299, E11331, E113311, E113312, E113313, E113319, E11339, E113391, E113392, E113393, E113399, E11341, E113411, E113412, E113413, E113419, E11349, E113491, E113492, E113493, E113499, E11351, E113511, E113512, E113513, E113519, E113521, E113522, E113523, E113529, E113531, E113532, E113533, E113539, E113541, E113542, E113543, E113549, E113551, E113552, E113553, E113559, E11359, E113591, E113592, E113593, E113599, E1136, E1137X1, E1137X2, E1137X3, E1137X9, E1139, E1140, E1141, E1142, E1143, E1144, E1149, E1151, E1152, E1159, E11610, E11618, E11620, E11621, E11622, E11628, E11630, E11638, E11641, E11649, E1165, E1169, E118, E1321, E1322, E1329, E13311, E13319, E13321, E133211, E133212, E133213, E133219, E13329, E133291, E133292, E133293, E133299, E13331, E133311, E133312, E133313, E133319, E13339, E133391, E133392, E133393, E133399, E13341, E133411, E133412, E133413, E133419, E13349, E133491, E133492, E133493, E133499, E13351, E133511, E133512, E133513, E133519, E133521, E133522, E133523, E133529, E133531, E133532, E133533, E133539, E133541, E133542, E133543, E133549, E133551, E133552, E133553, E133559, E13359, E133591, E133592, E133593, E133599, E1336, E1337X1, E1337X2, E1337X3, E1337X9, E1339, E1340, E1341, E1342, E1343, E1344, E1349, E1351, E1352, E1359, E13610, E13618, E13620, E13621, E13622, E13628, E13630, E13638, E13641, E13649, E1365, E1369, E138, P702 |

© 2021 Asch DA et al. *JAMA Network Open*

| | |
|---|---|
| Substance Use Disorder | F1110, F1111, F11120, F11121, F11122, F11129, F1114, F11150, F11151, F11159, F11181, F11182, F11188, F1119, F1120, F1121, F11220, F11221, F11222, F11229, F1123, F1124, F11250, F11251, F11259, F11281, F11282, F11288, F1129, F1210, F1211, F12120, F12121, F12122, F12129, F12150, F12151, F12159, F12180, F12188, F1219, F1220, F1221, F12220, F12221, F12222, F12229, F1223, F12250, F12251, F12259, F12280, F12288, F1229, F1310, F1311, F13120, F13121, F13129, F1314, F13150, F13151, F13159, F13180, F13181, F13182, F13188, F1319, F1320, F1321, F13220, F13221, F13229, F13230, F13231, F13232, F13239, F1324, F13250, F13251, F13259, F1326, F1327, F13280, F13281, F13282, F13288, F1329, F1410, F1411, F14120, F14121, F14122, F14129, F1414, F14150, F14151, F14159, F14180, F14181, F14182, F14188, F1419, F1420, F1421, F14220, F14221, F14222, F14229, F1423, F1424, F14250, F14251, F14259, F14280, F14281, F14282, F14288, F1429, F1510, F1511, F15120, F15121, F15122, F15129, F1514, F15150, F15151, F15159, F15180, F15181, F15182, F15188, F1519, F1520, F1521, F15220, F15221, F15222, F15229, F1523, F1524, F15250, F15251, F15259, F15280, F15281, F15282, F15288, F1529, F1610, F1611, F16120, F16121, F16122, F16129, F1614, F16150, F16151, F16159, F16180, F16183, F16188, F1619, F1620, F1621, F16220, F16221, F16229, F1624, F16250, F16251, F16259, F16280, F16283, F16288, F1629, F1810, F1811, F18120, F18121, F18129, F1814, F18150, F18151, F18159, F1817, F18180, F18188, F1819, F1820, F1821, F18220, F18221, F18229, F1824, F18250, F18251, F18259, F1827, F18280, F18288, F1829, F1910, F1911, F19120, F19121, F19122, F19129, F1914, F19150, F19151, F19159, F1916, F1917, F19180, F19181, F19182, F19188, F1919, F1920, F1921, F19220, F19221, F19222, F19229, F19230, F19231, F19232, F19239, F1924, F19250, F19251, F19259, F1926, F1927, F19280, F19281, F19282, F19288, F1929, F550, F551, F552, F553, F554, F558, O99320, O99321, O99322, O99323, O99324, O99325 |
| Hypertension | I10, I110, I119, I120, I129, I130, I1310, I1311, I132, I150, I151, I152, I158, I159, I160, I161, I169, I674, O10011, O10012, O10013, O10019, O1002, O1003, O10111, O10112, O10113, O10119, O1012, O1013, O10211, O10212, O10213, O10219, O1022, O1023, O10311, O10312, O10313, O10319, O1032, O1033, O10411, O10412, O10413, O10419, O1042, O1043, O10911, O10912, O10913, O10919, O1092, O1093, O111, O112, O113, O114, O115, O119, O161, O162, O163, O164, O165, O169 |
| Hypothyroidism | E000, E001, E002, E009, E018, E02, E030, E031, E032, E033, E038, E039, E890 |
| Liver Disease | B180, B181, B182, I8500, I8501, I8510, I8511, K700, K702, K7030, K7031, K7040, K7041, K709, K7210, K7211, K7290, K7291, K730, K731, K732, K738, K739, K740, K741, K742, K743, K744, K745, K7460, K7469, K754, K7581, K760, K766, K7689, K769, Z944 |

© 2021 Asch DA et al. *JAMA Network Open*

| | |
|---|---|
| Lymphoma | C8100, C8101, C8102, C8103, C8104, C8105, C8106, C8107, C8108, C8109, C8110, C8111, C8112, C8113, C8114, C8115, C8116, C8117, C8118, C8119, C8120, C8121, C8122, C8123, C8124, C8125, C8126, C8127, C8128, C8129, C8130, C8131, C8132, C8133, C8134, C8135, C8136, C8137, C8138, C8139, C8140, C8141, C8142, C8143, C8144, C8145, C8146, C8147, C8148, C8149, C8170, C8171, C8172, C8173, C8174, C8175, C8176, C8177, C8178, C8179, C8190, C8191, C8192, C8193, C8194, C8195, C8196, C8197, C8198, C8199, C8200, C8201, C8202, C8203, C8204, C8205, C8206, C8207, C8208, C8209, C8210, C8211, C8212, C8213, C8214, C8215, C8216, C8217, C8218, C8219, C8220, C8221, C8222, C8223, C8224, C8225, C8226, C8227, C8228, C8229, C8230, C8231, C8232, C8233, C8234, C8235, C8236, C8237, C8238, C8239, C8240, C8241, C8242, C8243, C8244, C8245, C8246, C8247, C8248, C8249, C8250, C8251, C8252, C8253, C8254, C8255, C8256, C8257, C8258, C8259, C8260, C8261, C8262, C8263, C8264, C8265, C8266, C8267, C8268, C8269, C8280, C8281, C8282, C8283, C8284, C8285, C8286, C8287, C8288, C8289, C8290, C8291, C8292, C8293, C8294, C8295, C8296, C8297, C8298, C8299, C8300, C8301, C8302, C8303, C8304, C8305, C8306, C8307, C8308, C8309, C8310, C8311, C8312, C8313, C8314, C8315, C8316, C8317, C8318, C8319, C8330, C8331, C8332, C8333, C8334, C8335, C8336, C8337, C8338, C8339, C8350, C8351, C8352, C8353, C8354, C8355, C8356, C8357, C8358, C8359, C8370, C8371, C8372, C8373, C8374, C8375, C8376, C8377, C8378, C8379, C8380, C8381, C8382, C8383, C8384, C8385, C8386, C8387, C8388, C8389, C8390, C8391, C8392, C8393, C8394, C8395, C8396, C8397, C8398, C8399, C8400, C8401, C8402, C8403, C8404, C8405, C8406, C8407, C8408, C8409, C8410, C8411, C8412, C8413, C8414, C8415, C8416, C8417, C8418, C8419, C8440, C8441, C8442, C8443, C8444, C8445, C8446, C8447, C8448, C8449, C8460, C8461, C8462, C8463, C8464, C8465, C8466, C8467, C8468, C8469, C8470, C8471, C8472, C8473, C8474, C8475, C8476, C8477, C8478, C8479, C8490, C8491, C8492, C8493, C8494, C8495, C8496, C8497, C8498, C8499, C84A0, C84A1, C84A2, C84A3, C84A4, C84A5, C84A6, C84A7, C84A8, C84A9, C84Z0, C84Z1, C84Z2, C84Z3, C84Z4, C84Z5, C84Z6, C84Z7, C84Z8, C84Z9, C8510, C8511, C8512, C8513, C8514, C8515, C8516, C8517, C8518, C8519, C8520, C8521, C8522, C8523, C8524, C8525, C8526, C8527, C8528, C8529, C8580, C8581, C8582, C8583, C8584, C8585, C8586, C8587, C8588, C8589, C8590, C8591, C8592, C8593, C8594, C8595, C8596, C8597, C8598, C8599, C860, C861, C862, C863, C864, C865, C866, C880, C882, C883, C884, C888, C889, C9000, C9001, C9002, C9010, C9011, C9012, C9020, C9021, C9022, C9030, C9031, C9032, C960, C962, C9620, C9621, C9622, C9629, C964, C969, C96A, C96Z, D47Z9 |
| Fluid and Electrolyte Disorder | E860, E861, E869, E870, E871, E872, E873, E874, E875, E876, E8770, E8771, E8779, E878 |
| Metastatic Cancer | C770, C771, C772, C773, C774, C775, C778, C779, C7800, C7801, C7802, C781, C782, C7830, C7839, C784, C785, C786, C787, C7880, C7889, C7900, C7901, C7902, C7910, C7911, C7919, C792, C7931, C7932, C7940, C7949, C7951, C7952, C7960, C7961, C7962, C7970, C7971, C7972, C7981, C7982, C7989, C799, C7B00, C7B01, C7B02, C7B03, C7B04, C7B09, C7B1, C7B8, C800, C801, R180 |

© 2021 Asch DA et al. *JAMA Network Open*

| | |
|---|---|
| Neurological Disorder | E7500, E7501, E7502, E7509, E7510, E7511, E7519, E7523, E7525, E7526, E7529, E754, F842, G10, G110, G111, G112, G113, G114, G118, G119, G120, G121, G1220, G1221, G1222, G1223, G1224, G1225, G1229, G128, G129, G132, G138, G20, G214, G2401, G2402, G2409, G242, G248, G254, G255, G2581, G300, G301, G308, G309, G3101, G3109, G311, G312, G3181, G3182, G3183, G3184, G3185, G3189, G319, G3281, G35, G361, G368, G369, G370, G371, G372, G373, G374, G375, G378, G379, G40001, G40009, G40011, G40019, G40101, G40109, G40111, G40119, G40201, G40209, G40211, G40219, G40301, G40309, G40311, G40319, G40401, G40409, G40411, G40419, G40501, G40509, G40801, G40802, G40803, G40804, G40811, G40812, G40813, G40814, G40821, G40822, G40823, G40824, G4089, G40901, G40909, G40911, G40919, G40A01, G40A09, G40A11, G40A19, G40B01, G40B09, G40B11, G40B19, G47411, G47419, G47421, G47429, G803, G890, G910, G911, G912, G913, G914, G918, G919, G937, G9389, G939, G94, O99350, O99351, O99352, O99353, O99354, O99355, P9160, P9161, P9162, P9163, R410, R4182, R4701, R5600, R5601, R561, R569 |
| Obesity | E6601, E6609, E661, E662, E668, E669, O99210, O99211, O99212, O99213, O99214, O99215, R939, Z6830, Z6831, Z6832, Z6833, Z6834, Z6835, Z6836, Z6837, Z6838, Z6839, Z6841, Z6842, Z6843, Z6844, Z6845, Z6854 |
| Paralysis | G041, G800, G801, G802, G804, G808, G809, G8100, G8101, G8102, G8103, G8104, G8110, G8111, G8112, G8113, G8114, G8190, G8191, G8192, G8193, G8194, G8220, G8221, G8222, G8250, G8251, G8252, G8253, G8254, G830, G8310, G8311, G8312, G8313, G8314, G8320, G8321, G8322, G8323, G8324, G8330, G8331, G8332, G8333, G8334, G834, G835, G8381, G8382, G8383, G8384, G8389, G839, I69031, I69032, I69033, I69034, I69039, I69041, I69042, I69043, I69044, I69049, I69051, I69052, I69053, I69054, I69059, I69061, I69062, I69063, I69064, I69065, I69069, I69131, I69132, I69133, I69134, I69139, I69141, I69142, I69143, I69144, I69149, I69151, I69152, I69153, I69154, I69159, I69161, I69162, I69163, I69164, I69165, I69169, I69231, I69232, I69233, I69234, I69239, I69241, I69242, I69243, I69244, I69249, I69251, I69252, I69253, I69254, I69259, I69261, I69262, I69263, I69264, I69265, I69269, I69331, I69332, I69333, I69334, I69339, I69341, I69342, I69343, I69344, I69349, I69351, I69352, I69353, I69354, I69359, I69361, I69362, I69363, I69364, I69365, I69369, I69831, I69832, I69833, I69834, I69839, I69841, I69842, I69843, I69844, I69849, I69851, I69852, I69853, I69854, I69859, I69861, I69862, I69863, I69864, I69865, I69869, I69931, I69932, I69933, I69934, I69939, I69941, I69942, I69943, I69944, I69949, I69951, I69952, I69953, I69954, I69959, I69961, I69962, I69963, I69964, I69965, I69969, R532 |

© 2021 Asch DA et al. *JAMA Network Open*

| | |
|---|---|
| Peripheral Vascular Disease | I700, I701, I70201, I70202, I70203, I70208, I70209, I70211, I70212, I70213, I70218, I70219, I70221, I70222, I70223, I70228, I70229, I70231, I70232, I70233, I70234, I70235, I70238, I70239, I70241, I70242, I70243, I70244, I70245, I70248, I70249, I7025, I70261, I70262, I70263, I70268, I70269, I70291, I70292, I70293, I70298, I70299, I70301, I70302, I70303, I70308, I70309, I70311, I70312, I70313, I70318, I70319, I70321, I70322, I70323, I70328, I70329, I70331, I70332, I70333, I70334, I70335, I70338, I70339, I70341, I70342, I70343, I70344, I70345, I70348, I70349, I7035, I70361, I70362, I70363, I70368, I70369, I70391, I70392, I70393, I70398, I70399, I70401, I70402, I70403, I70408, I70409, I70411, I70412, I70413, I70418, I70419, I70421, I70422, I70423, I70428, I70429, I70431, I70432, I70433, I70434, I70435, I70438, I70439, I70441, I70442, I70443, I70444, I70445, I70448, I70449, I7045, I70461, I70462, I70463, I70468, I70469, I70491, I70492, I70493, I70498, I70499, I70501, I70502, I70503, I70508, I70509, I70511, I70512, I70513, I70518, I70519, I70521, I70522, I70523, I70528, I70529, I70531, I70532, I70533, I70534, I70535, I70538, I70539, I70541, I70542, I70543, I70544, I70545, I70548, I70549, I7055, I70561, I70562, I70563, I70568, I70569, I70591, I70592, I70593, I70598, I70599, I70601, I70602, I70603, I70608, I70609, I70611, I70612, I70613, I70618, I70619, I70621, I70622, I70623, I70628, I70629, I70631, I70632, I70633, I70634, I70635, I70638, I70639, I70641, I70642, I70643, I70644, I70645, I70648, I70649, I7065, I70661, I70662, I70663, I70668, I70669, I70691, I70692, I70693, I70698, I70699, I70701, I70702, I70703, I70708, I70709, I70711, I70712, I70713, I70718, I70719, I70721, I70722, I70723, I70728, I70729, I70731, I70732, I70733, I70734, I70735, I70738, I70739, I70741, I70742, I70743, I70744, I70745, I70748, I70749, I7075, I70761, I70762, I70763, I70768, I70769, I70791, I70792, I70793, I70798, I70799, I708, I7090, I7091, I7092, I7100, I7101, I7102, I7103, I711, I712, I713, I714, I715, I716, I718, I719, I720, I721, I722, I723, I724, I725, I726, I728, I729, I731, I7381, I7389, I739, I742, I743, I744, I76, I771, I7770, I7771, I7772, I7773, I7774, I7775, I7776, I7777, I7779, I790, I791, I798, K551, K558, K559, Z95820, Z95828 |
| Psychosis | F200, F201, F202, F203, F205, F2081, F2089, F209, F22, F23, F24, F250, F251, F258, F259, F28, F29, F3010, F3011, F3012, F3013, F302, F303, F304, F308, F309, F310, F3110, F3111, F3112, F3113, F312, F3130, F3131, F3132, F314, F315, F3160, F3161, F3162, F3163, F3164, F3170, F3171, F3172, F3173, F3174, F3175, F3176, F3177, F3178, F3181, F3189, F319, F324, F325, F3340, F3341, F3342, F348, F3481, F3489, F349, F39, F4489, F843 |
| Pulmonary Circulation | I2601, I2602, I2609, I2690, I2692, I2699, I270, I271, I2781, I2782, I2783, I2789, I279, I289, T800XXA, T82817A, T82818A |
| Chronic Kidney Disease | I120, I1311, I132, N183, N184, N185, N186, N189, N19, Z4901, Z4902, Z4931, Z4932, Z9115, Z940, Z992 |

© 2021 Asch DA et al. *JAMA Network Open*

| | |
|---|---|
| Solid Tumor without Metastasis | C000, C001, C002, C003, C004, C005, C006, C008, C009, C01, C020, C021, C022, C023, C024, C028, C029, C030, C031, C039, C040, C041, C048, C049, C050, C051, C052, C058, C059, C060, C061, C062, C0680, C0689, C069, C07, C080, C081, C089, C090, C091, C098, C099, C100, C101, C102, C103, C104, C108, C109, C110, C111, C112, C113, C118, C119, C12, C130, C131, C132, C138, C139, C140, C142, C148, C153, C154, C155, C158, C159, C160, C161, C162, C163, C164, C165, C166, C168, C169, C170, C171, C172, C173, C178, C179, C180, C181, C182, C183, C184, C185, C186, C187, C188, C189, C19, C20, C210, C211, C212, C218, C220, C221, C222, C223, C224, C227, C228, C229, C23, C240, C241, C248, C249, C250, C251, C252, C253, C254, C257, C258, C259, C260, C261, C269, C300, C301, C310, C311, C312, C313, C318, C319, C320, C321, C322, C323, C328, C329, C33, C3400, C3401, C3402, C3410, C3411, C3412, C342, C3430, C3431, C3432, C3480, C3481, C3482, C3490, C3491, C3492, C37, C380, C381, C382, C383, C384, C388, C390, C399, C4000, C4001, C4002, C4010, C4011, C4012, C4020, C4021, C4022, C4030, C4031, C4032, C4080, C4081, C4082, C4090, C4091, C4092, C410, C411, C412, C413, C414, C419, C430, C4310, C4311, C43111, C43112, C4312, C43121, C43122, C4320, C4321, C4322, C4330, C4331, C4339, C434, C4351, C4352, C4359, C4360, C4361, C4362, C4370, C4371, C4372, C438, C439, C450, C451, C452, C457, C470, C4710, C4711, C4712, C4720, C4721, C4722, C473, C474, C475, C476, C478, C479, C480, C481, C482, C488, C490, C4910, C4911, C4912, C4920, C4921, C4922, C493, C494, C495, C496, C498, C499, C49A0, C49A1, C49A2, C49A3, C49A4, C49A5, C49A9, C4A0, C4A10, C4A11, C4A111, C4A112, C4A12, C4A121, C4A122, C4A20, C4A21, C4A22, C4A30, C4A31, C4A39, C4A4, C4A51, C4A52, C4A59, C4A60, C4A61, C4A62, C4A70, C4A71, C4A72, C4A8, C4A9, C50011, C50012, C50019, C50021, C50022, C50029, C50111, C50112, C50119, C50121, C50122, C50129, C50211, C50212, C50219, C50221, C50222, C50229, C50311, C50312, C50319, C50321, C50322, C50329, C50411, C50412, C50419, C50421, C50422, C50429, C50511, C50512, C50519, C50521, C50522, C50529, C50611, C50612, C50619, C50621, C50622, C50629, C50811, C50812, C50819, C50821, C50822, C50829, C50911, C50912, C50919, C50921, C50922, C50929, C510, C511, C512, C518, C519, C52, C530, C531, C538, C539, C540, C541, C542, C543, C548, C549, C55, C561, C562, C569, C5700, C5701, C5702, C5710, C5711, C5712, C5720, C5721, C5722, C573, C574, C577, C578, C579, C58, C600, C601, C602, C608, C609, C61, C6200, C6201, C6202, C6210, C6211, C6212, C6290, C6291, C6292, C6300, C6301, C6302, C6310, C6311, C6312, C632, C637, C638, C639, C641, C642, C649, C651, C652, C659, C661, C662, C669, C670, C671, C672, C673, C674, C675, C676, C677, C678, C679, C680, C681, C688, C689, C6900, C6901, C6902, C6910, C6911, C6912, C6920, C6921, C6922, C6930, C6931, C6932, C6940, C6941, C6942, C6950, C6951, C6952, C6960, C6961, C6962, C6980, C6981, C6982, C6990, C6991, C6992, C700, C701, C709, C710, C711, C712, C713, C714, C715, C716, C717, C718, C719, C720, C721, C7220, C7221, C7222, C7230, C7231, C7232, C7240, C7241, C7242, C7250, C7259, C729, C73, C7400, C7401, C7402, C7410, C7411, C7412, C7490, C7491, C7492, C750, C751, C752, C753, C754, C755, C758, C759, C760, C761, C762, C763, C7640, C7641, C7642, C7650, C7651, C7652, C768, C7A00, C7A010, C7A011, C7A012, C7A019, C7A020, C7A021, C7A022, C7A023, C7A024, C7A025, C7A026, C7A029, C7A090, C7A091, C7A092, C7A093, C7A094, C7A095, C7A096, C7A098, D030, D0310, D0311, D03111, D03112, D0312, D03121, D03122, D0320, D0321, D0322, D0330, D0339, D034, D0351, D0352, D0359, D0360, D0361, D0362, D0370, D0371, D0372, D038, D039, E3121, E3122, E3123 |
| Peptic Ulcer | K254, K255, K256, K257, K259, K264, K265, K266, K267, K269, K274, K275, K276, K277, K279, K284, K285, K286, K287, K289 |
| Valvular Disorder | A5203, I050, I051, I052, I058, I059, I060, I061, I062, I068, I069, I070, I071, I072, I078, I079, I080, I081, I082, I083, I088, I089, I091, I0989, I340, I341, I342, I348, I349, I350, I351, I352, I358, I359, I360, I361, I362, I368, I369, I370, I371, I372, I378, I379, I38, I39, Q230, Q231, Q232, Q233, Z952, Z953, Z954 |
| Weight Loss | E40, E41, E42, E43, E440, E441, E45, E46, E640, R634, R636 |

© 2021 Asch DA et al. *JAMA Network Open*

**eAppendix 2.** An illustration of estimating mortality differences in Black and white patients based on a simulated dataset

Md Nazmul Islam

11/16/2020

## Necessary functions

We load the necessary packages and define the inputs and outputs associated with the main functions used in the illustration. Next we spell out the analytical functions used in the manuscript.

```
library(glmmTMB); library(glmmML); library(GLMMadaptive); library(MASS);
```

```
##

## Attaching package: 'MASS'
```

```
## The following object is masked from 'package:GLMMadaptive':##

##           negative.binomial
```

```
library(dplyr); library(DescTools); library(MuMIn); library(psych);
```

```
##

## Attaching package: 'dplyr'
```

```
## The following object is masked from 'package:MASS':##

##           select
```

© 2021 Asch DA et al. *JAMA Network Open*

```
## The following objects are masked from 'package:stats':##

##        filter, lag
```

```
## The following objects are masked from 'package:base':##

##        intersect, setdiff, setequal, union
```

```
##

## Attaching package: 'DescTools'
```

```
## The following object is masked from 'package:GLMMadaptive':##

##        VIF
```

```
## Registered S3 methods overwritten by 'MuMIn':
##     method            from
##     [illegible]
##     summary.glmmML glmmML
```

```
##

## Attaching package: 'psych'
```

```
## The following objects are masked from 'package:DescTools':##

##        AUC, ICC, SD
```

```
library(performance);library(rpivotTable);library(ggplotify);library(pastecs)
```

```
##

## Attaching package: 'pastecs'
```

```
## The following objects are masked from 'package:dplyr':##

##          first, last
```

```
library(lmtest); library(pscl); library(ResourceSelection); library(LogisticDx)
```

```
## Loading required package: zoo
```

```
##

## Attaching package: 'zoo'
```

```
## The following objects are masked from 'package:base':##

##          as.Date, as.Date.numeric
```

```
## Classes and Methods for R developed in the## Political Science
Computational Laboratory## Department of Political Science

## Stanford University## Simon
Jackman

## hurdle and zeroinfl functions by Achim Zeileis
```

```
## ResourceSelection 0.3-5                    2019-07-22
```

```
library(ggplot2); library(gridExtra)
```

```
##

## Attaching package: 'ggplot2'
```

```
## The following objects are masked from 'package:psych':##

##            %+%, alpha
```

```
##

## Attaching package: 'gridExtra'
```

```
## The following object is masked from 'package:dplyr':##

##            combine
```

© 2021 Asch DA et al. *JAMA Network Open*

```
## Definitions (input)

# ylist = List of possible response variable names; "mbr_death_flag", "mbr_composite_flag, "mbr_hospice_flag"
# ylist_cat = Main variable of interest (e.g., mbr_composite_flag)

# variable_list = List of variables of interest separated by ":" e.g. "Age:Race:Comorbidtity"

#                 will fit model with a list of covariates such as age, race, and 29 Elixhauser comorbidities
# comorb_name = List of comorbidity variable names (comorb_id) and full names (comorb_name)

# Data2 = Dataframe containing reponse & covariates after arranging in appropriate category
# maxnum = 2; Integer indicating minimum number of patients per site

# reml = TRUE; Boolean for REML, otherwise ML (Primarily used for random effect estimation in glmmTMB)
# bootH = Number of bootstrap in glmmML for hypothesis testing by permutation

# ranlist = Random effect; "(1 | hosp_name)"

# refh = "Hospital_1"; Reference hospital name (must be a character)

# state = FALSE; logical whether we add "state" (e.g., Alabama) in the model
# refs = NULL; Reference for state

# mlist_race = "race_cd"; Name of race variable in dataset
# varlist_race = "Race::african-american"; Race of interest
# GLM = TRUE; Fits multiple generalized linear model

# GLM_FX = FALSE; Boolean if TRUE hospitals are modeled as fixed effects

# GLM_ML = FALSE; Boolean if TRUE profiling likelihood method is used to estimate parameters
#                  treating hospital effects as nuisance parameters (computationally fast)

# GLMM_RE = FALSE; Boolean if TRUE hospitals are modeled as random effects

# addsize = TRUE; Boolean if TRUE size (e.g., bed size) is added in computing scores in hierarchical model
# laplace = TRUE; Boolean if TRUE laplace approximation is adopted; otherwise

#                  approximation of integrand is considered via Gaussian quadrature with 11 quadrature points
# fixed = TRUE; Boolean if TRUE fixed hospital effects are computed

# hospital = FALSE; Boolean if TRUE hospital effects are estimated either by fixed or random parameters
# fitr = Fitted glm() / glmmTMB() / glmmML() object

# fit_type = "glm_wot" (without hospital effect) / glm_w (with hospital effect) / "glmmML" (profile)
# nameb = a dataframe with RSER, hospital names, white-and-Black population and proportion,
```

© 2021 Asch DA et al. *JAMA Network Open*

```
#           corresponding observed Black death rate
# totalb = Total number of Black patients

# totalw = Total number of white patients

# method = "1-step"; Method to assign Black patients based on Multinomial distribution


## Definitions (output)

# score_silber = Site-specific RSER based on Silber et al.
# score_drye = Site-specific RSER based on Drye et al.

# score_DS = Matrix of RSERs N x K; N (number of patients) and K (number of sites)
# reference = Unique site names

# LRT_re = LRT of random effects
```

© 2021 Asch DA et al. *JAMA Network Open*

```
# aic_null = Akaike information criteria for GLM model
# aic_alt = Akaike information criteria for GLMM model

# SS = Marginal & conditional R-squared (goodness of fit) for GLMM
# Summary = Summary of GLMM fit

# cor_lme_lmer = Correlation of fixed effects between GLM and GLMM

# rank_cor = Correlation between predicted probabilities and observed responses (C and Dxy statistic) for GLMM
# fom_w_hosp = Fit object with fixed hospital effects

# fom_wot_hosp = Fit object withot fixed hospital effects
# fom_rand_hosp = Fit object with random hospital effects

# fom_w_hosp_pro = Fit object (profile) with fixed nuisance hospital effects

# blk_glmm = Recycled link function predictions for Black (based on mixed model)
# wht_glmm = Recycled link function predictions for white (based on mixed model)

# blk_glm_fx = Recycled link function predictions for Black (based on fixed hospital)
# wht_glm_fx = Recycled link function predictions for white (based on fixed hospital)

# blk_glm_wot = Recycled link function predictions for Black (without hospital effect model)
# wht_glm_wot = Recycled link function predictions for white (without hospital effect model)
```

© 2021 Asch DA et al. *JAMA Network Open*

```
# A1. Create categorical data (Multiple rows per site)
arrange_dataframe_race_wothosp <- function(data){

  ## Income variables
  data$mbr_income_class <- ifelse(data$mbr_income < 50000, "50K > Income",
                                  ifelse(data$mbr_income >= 50000 & data$mbr_income < 70000, "50K <= Income < 70K",
                                         ifelse(data$mbr_income >= 70000, "70K <= Income", NA)))

  table(data$mbr_income_class)

  ## Counts of days since Feb1
  count_day <- as.numeric(c(data$mbr_admit_dt - as.Date("2020-01-01"))) # year-month-day
  count_day_class <- ifelse(count_day <= 90, "1 < Count <= 90",
                            ifelse(count_day > 90 & count_day <= 120, "90 < Count <= 120",
                                   ifelse(count_day > 120 & count_day <= 150, "120 < Count <= 150",
                                          ifelse(count_day > 150 & count_day <= 180, "150 < Count <= 180",
                                                 #ifelse(count_day > 180 & count_day <= 210, "180 < Count <= 210",
                                                 "180 < Count"))))
  data$count_day  <- count_day
  data$count_day2 <- count_day^2

  data$count_day_class <- factor(count_day_class, levels = c("1 < Count <= 90", "90 < Count <= 120",
                                                             "120 < Count <= 150", "150 < Count <= 180",
                                                             "180 < Count"))

  data$gender <- ifelse(data$gender == "m", "Male",
                        ifelse(data$gender == "f", "Female", NA))
```

© 2021 Asch DA et al. *JAMA Network Open*

```
ixf <- grep("aeci_", colnames(data),   value   = F)

QQ <- paste0("data$", colnames(data)[ixf]," <- data %>% select(", colnames(data)[ixf],") %>%replace(is.na(.), 0) %>%
                    mutate(", colnames(data)[ixf]," = ifelse(", colnames(data)[ixf]," > 0, 'Yes', 'No')) %>%unlist()",
                    collapse = ";")QQ
<- eval(parse(text = QQ))

comorb_id <- grep("aeci_", colnames(data),   value   = T)
#data$mapd_comm_flag  <-  ifelse(data$lob  ==  "medicare",  "Medicare",  "Commercial")
```

© 2021 Asch DA et al. *JAMA Network Open*

```
# age class

data$age_class <- ifelse(data$age <= 55, "18 < age <= 55",
        ifelse(data$age > 55 & data$age <= 65, "55 < age <= 65",
            ifelse(data$age > 65 & data$age <= 75, "65 < age <= 75",
                ifelse(data$age > 75 & data$age <= 85, "75 < age <= 85",
                    "85 < age"))))

table(data$age_class)



data$race_cd[which(is.na(data$race_cd) == TRUE)] <- "unknown"

data$race_cd_class <- ifelse(data$race_cd %in% c("african-american", "hispanic", "native american",
                                    "other", "asian"), "Non-caucasian",
        ifelse(data$race_cd %in% "caucasian", "caucasian", "unknown"))



data$mbr_death_flag <- ifelse(data$mbr_death_flag == 1, "Death", "Alive")
data$mbr_death_flag   <- data$mbr_death_flag %>% replace(is.na(.), "Alive")


data$mbr_hospice_flag <- ifelse(data$mbr_hospice_flag == 1, "Hospice", "Alive")
data$mbr_hospice_flag   <- data$mbr_hospice_flag %>% replace(is.na(.), "Alive")


data$mbr_composite_flag <- ifelse(data$mbr_death_flag == "Death" | data$mbr_hospice_flag == "Hospice",

        "Event", "Alive")


data$snf_nursing_flag <- ifelse(data$snf_nursing_flag == 1, "Yes", "No") data$snf_nursing_flag
<- data$snf_nursing_flag %>% replace(is.na(.), "No")


data$hosp_region <- ifelse(data$hosp_region == "ct", "Central",

        ifelse(data$hosp_region == "ne", "North-east",
            ifelse(data$hosp_region == "se", "South-east",

                ifelse(data$hosp_region == "we", "West", "Other"))))
```

© 2021 Asch DA et al. *JAMA Network Open*

```
data$hosp_usr_class <- ifelse(data$hosp_usr_class == "U", "Urban",
                              ifelse(data$hosp_usr_class == "R", "Rural",
                                     ifelse(data$hosp_usr_class == "S", "Semi-urban",
                                            "Other")))

data$mbr_ses <- ifelse(data$mbr_ses <= 45, "(0,45]",
```

© 2021 Asch DA et al. *JAMA Network Open*

```
                                ifelse(data$mbr_ses > 45 & data$mbr_ses <= 55, "(45,55]",
                                       ">55"))

  output = list(data = data, comorb_id = comorb_id)
}

# A2. Not exist
'%!in%' <- function(x, y) !('%in%'(x, y))

# A3. Functions to select sites with at-least 1 Black and 1 white patient
b1w1 <- function(Main2, racet = ">=1 non-white - >=1 white"){
  ## Order hospital name
  Main2 <- Main2[order(Main2$hosp_name), ]

  ## atleast 1 white / 1 non-white
  a1 <- unique(Main2$hosp_name)
  a2 <- unlist(lapply(seq_len(length(a1)), function(ii) {

    r1 <- which(Main2$hosp_name == a1[ii])
    if(racet == ">=1 non-white - >=1 white"){r2 <-
      table(Main2$race_cd_class[r1]) if(length(r2) <
      2){
        0 # donot add this site
      }else{
        1 # add this site
      }
    } else if(racet == ">=1 black - >=1 white"){r2 <-
      table(Main2$race_cd[r1]) if("african-american"
      %in% names(r2)){
        1
```

```
      }else{
        0# no-black for this site
      }
    }

})) 
table(a2)

del_site <- a1[which(a2 == 0)]
Main2 <- Main2[which(Main2$hosp_name %!in% del_site), ]
# removing these sites as they don't meet 1 white/nonwhite
```

© 2021 Asch DA et al. *JAMA Network Open*

```
    Main2 <- Main2[order(Main2$hosp_name), ] # ordering


    output = list(Main2 = Main2)

}

# A4. Filtering function

filtering_function <- function(AA, BB, CC, DD, EE, FF, Main){


    Main2G <- Main

    if(AA != "none"){
        mystring <- AA

        splt <- unlist(strsplit(mystring, "::"))AA1 <- splt[1]

        AA2 <- ifelse(splt[2] == "Yes", 'Yes', ifelse(splt[2] == "No", 'No', splt[2]))

        filter <- paste0("which(Main2G$", AA1, " == '", AA2, "')", sep = "")filter <-
        eval(parse(text = filter))

        if(length(filter) > 0){

            Main2G <- Main2G[filter, ] # creating subset by filters

        }else{

            Main2G <- Main2G

        }

    }

    if(BB != "none"){

        mystring <- BB

        if(mystring == ">= 1mo in 2019"){ filter2 <-
            which(Main2G$mm_2019 >= 1)

        } else if(mystring == ">= 2mo in 2019"){filter2
            <- which(Main2G$mm_2019 >= 2)

        } else if(mystring == ">= 3mo in 2019"){filter2
            <- which(Main2G$mm_2019 >= 3)
```

© 2021 Asch DA et al. *JAMA Network Open*

```
} else if(mystring == ">= 4mo in 2019"){filter2
    <- which(Main2G$mm_2019 >= 4)
} else if(mystring == ">= 5mo in 2019"){filter2
    <- which(Main2G$mm_2019 >= 5)
} else if(mystring == ">= 6mo in 2019"){filter2
    <- which(Main2G$mm_2019 >= 6)
} else if(mystring == ">= 7mo in 2019"){filter2
    <- which(Main2G$mm_2019 >= 7)
} else if(mystring == ">= 8mo in 2019"){filter2
    <- which(Main2G$mm_2019 >= 8)
```

© 2021 Asch DA et al. *JAMA Network Open*

```
    } else if(mystring == ">= 9mo in 2019"){filter2
      <- which(Main2G$mm_2019 >= 9)
    } else if(mystring == ">= 10mo in 2019"){filter2 <-
      which(Main2G$mm_2019 >= 10)
    } else if(mystring == ">= 11mo in 2019"){filter2 <-
      which(Main2G$mm_2019 >= 11)
    } else if(mystring == "12mo in 2019"){ filter2 <-
      which(Main2G$mm_2019 >= 12)

    }

    if(length(filter2) > 0){

      Main2G <- Main2G[filter2, ] # creating subset by filters

    }else{

      Main2G <- Main2G

    }

  }

  CC_num <- 1

  if(CC != "none"){

    CC_num <- as.numeric(strsplit(CC, ">=")[[1]][2])

    ht <- aggregate(Main2G$hosp_name, by = list(Main2G$hosp_name), length)select_hosp
    <- ht$Group.1[which(ht$x >= CC_num)] if(length(select_hosp) > 0){

      Main2G <- Main2G[Main2G$hosp_name %in% select_hosp, ]

    }else{

      Main2G <- Main2G

    }

  }

  if(DD != "none"){# lob

    filter4 <- which(Main2G$mapd_comm_flag == DD)


    if(length(filter4) > 0){
```

© 2021 Asch DA et al. *JAMA Network Open*

```
        Main2G <- Main2G[filter4, ] # creating subset by filters
    }else{
        Main2G <- Main2G
    }
}
if(EE != "none"){
    filter5 <- which(Main2G$age >=
                                as.numeric(strsplit(EE, ">=")[[1]][2]))
    if(length(filter5) > 0){
        Main2G <- Main2G[filter5, ] # creating subset by filters
    }else{
```

© 2021 Asch DA et al. *JAMA Network Open*

```
        Main2G <- Main2G
      }
    }
    if(FF != "none"){
      filter6 <- which(Main2G$disp_desc %!in% "discharged/transferred to another health care institution")
      if(length(filter6) > 0){
        Main2G <- Main2G[filter6, ] # creating subset by filters
      }else{
        Main2G <- Main2G
      }
    }
    output = list(Main2G = Main2G, CC_num = CC_num)
}

# A5. Setting lables in dataframe (OR)
function_character <- function(Data22, variable_list_BAR, count_variable = TRUE, refh, refs){symbol <- "\u2265";
    symbol2 <- intToUtf8(8804)

    Data22 <- Data22 %>% mutate_if(is.character, as.factor)

    ## Reference the last hospital
    Data22$hosp_name <- as.factor(Data22$hosp_name)
    if(refh  %in%  unique(Data22$hosp_name)){
      Data22 <- within(Data22, hosp_name   <- relevel(hosp_name, ref = refh))
    } else  if(refh %!in% unique(Data22$hosp_name)){ # possible that after applying 1b/1h, we may no longer have this refh (E.
G. West)
      Data22 <- within(Data22, hosp_name   <- relevel(hosp_name, ref = as.character(unique(Data22$hosp_name)[1])))
```

© 2021 Asch DA et al. *JAMA Network Open*

```
}

if(length(grep(variable_list_BAR, "State", value = TRUE)) > 0){
    Data22 <- within(Data22, hosp_state_full <- relevel(hosp_state_full, ref = refs))
}

if(length(grep(variable_list_BAR, "Income", value = TRUE)) > 0){

    Data22$mbr_income_class <- factor(Data22$mbr_income_class, levels = c("50K > Income",
                                                                          "50K <= Income < 70K",
                                                                          "70K <= Income"))
    Data22 <- within(Data22, mbr_income_class <- relevel(mbr_income_class, ref = "70K <= Income"))
}
```

© 2021 Asch DA et al. *JAMA Network Open*

```
if(length(grep(variable_list_BAR, "Race", value = TRUE)) > 0){
   #try(Data22 <- within(Data22, race_cd_class <- relevel(race_cd_class, ref = "caucasian")))
   try(Data22 <- within(Data22, race_cd <- relevel(race_cd, ref = "caucasian")))
}

if(length(grep(variable_list_BAR, "Age", value = TRUE)) > 0){
                              Data22$age_class <- factor(Data22$age_class, levels = c("18 < age <= 55", "55 < age <= 65",
                                                                                      "65 < age <= 75", "75 < age <= 85",
                                                                               "85 < age"))
                  Data22 <- within(Data22, age_class <- relevel(age_class, ref = "18 < age <= 55"))
}
if(length(grep(variable_list_BAR, "Gender", value = TRUE)) > 0){ Data22 <-
   within(Data22, gender <- relevel(gender, ref = "Female"))
}

if(count_variable  == TRUE){
   if(length(grep(variable_list_BAR, "Count", value = TRUE)) > 0){
      Data22$count_day_class <- factor(Data22$count_day_class, levels = c("1 < Count <= 90",
                                                                          "90 < Count <= 120",
                                                                          "120 < Count <= 150", "150 < Count <= 180",
                                                                          "180 < Count"))
      Data22 <- within(Data22, count_day_class <- relevel(count_day_class, ref = "180 < Count"))
   }
}

if(length(grep(variable_list_BAR, "Comorbidity", value = TRUE)) > 0){
```

© 2021 Asch DA et al. *JAMA Network Open*

```
    QQ <- paste0("Data22 <- within(Data22, ", comorb_id, " <- relevel(",comorb_id, ", ref = 'No'))", collapse = "; ")QQ <- eval(parse(text = QQ))

}

if(length(grep(variable_list_BAR, "Nurse", value = TRUE)) > 0){

    Data22 <- within(Data22, snf_nursing_flag   <- relevel(snf_nursing_flag, ref = "No"))

}


if(length(grep(variable_list_BAR, "Region", value = TRUE)) > 0){

    Data22 <- within(Data22, hosp_region <- relevel(hosp_region, ref = "Central"))

}


if(length(grep(variable_list_BAR, "Numeric", value = TRUE)) > 0){Data22$count_day <-
    scale(Data22$count_day)
```

© 2021 Asch DA et al. *JAMA Network Open*

```
        Data22$count_day2 <- scale(Data22$count_day2)

    }

    #try(Data22 <- within(Data22, mbr_death_flag <- relevel(mbr_death_flag, ref = "Alive")))
    #try(Data22 <- within(Data22, mbr_hospice_flag <- relevel(mbr_hospice_flag, ref = "Alive")))
    Data22 <- within(Data22, mbr_composite_flag <- relevel(mbr_composite_flag, ref = "Alive"))

    Data22 <- Data22[order(Data22$hosp_name), ] # order by hosp name

    output = list(Data22 = Data22)

}

# A6. Model fit :: glm + glmmML + glmmTMB

blm_model <- function(variable_list = "Age:Gender:Race:Nurse:Comorbidity:Count:Region:RaceCount",Data2, comorb_name
                      = list(comorb_nm, comorb_id),
                      ylist = c("mbr_composite_flag", "mbr_hospice_flag", "mbr_death_flag"),ylist_cat =
                      "mbr_composite_flag",
                      reml = FALSE, bootH = FALSE,ranlist
                      = "(1 | hosp_name)", refh =
                      "HospitalXX",
                      state = FALSE, refs =
                      "Alabama",
                      mlist_race = "race_cd",
                      varlist_race = "Race::african-american", GLM = TRUE,
                      GLM_FX = FALSE, GLM_ML = FALSE,GLMM_RE =
                      FALSE, laplace = FALSE) {

    symbol <- "\u2265"; symbol2 <- intToUtf8(8804)

    ylist_num <- paste0("", strsplit(ylist_cat, "flag")[[1]], "num")
```

© 2021 Asch DA et al. *JAMA Network Open*

```
mlist_race <- mlist_race; varlist_race <- varlist_race; reflist_race <- rep("Caucasian", length(varlist_race));mlist0_race <- rep(mlist_race, length(varlist_race))

mlist_age <- c("age_class"); varlist_age <- c("Age::[55,65)", "Age::[65,75)", "Age::[75,85)", paste0("Age::", symbol,"85"

)); reflist_age <- rep("[18,55)", length(varlist_age)); mlist0_age <- rep("age_class", length(varlist_age))

mlist_income <- c("mbr_income_class"); varlist_income <- c("Income::<50K", "Income::[50K,70K)"); reflist_income <- rep(paste0("Income::", symbol,"70K"), length(varlist_income)); mlist0_income <- rep("mbr_income_class", length(varlist_income))

mlist_gender <- c("gender"); varlist_gender <- c("Gender::Male"); reflist_gender <- rep("Female", length(varlist_gender));mlist0_gender <- rep("gender", length(varlist_gender))
```

© 2021 Asch DA et al. *JAMA Network Open*

```
comorb_nm <- comorb_name[[1]]; comorb_id <- comorb_name[[2]]

mlist_comorbidity <- c(comorb_id); varlist_comorbidity <- paste0("", comorb_nm, "::Yes", sep = ""); reflist_comorbidity <-rep("No",
length(varlist_comorbidity)); mlist0_comorbidity <- comorb_id

mlist_nurse <- c("snf_nursing_flag"); varlist_nurse <- c("Transferred from nursing facility::Yes"); reflist_nurse <- rep("No", length(varlist_nurse));
mlist0_nurse <- rep("snf_nursing_flag", length(varlist_nurse))

mlist_count <- c("count_day_class"); varlist_count <- c("Days from January 1,2020::[0,90)",
                                                         "Days from January 1,2020::[90,120)","Days
                                                         from January 1,2020::[120,150)","Days from
                                                         January 1,2020::[150,180)")

reflist_count <- rep(paste0("", symbol,"180"), length(varlist_count));mlist0_count <-
rep("count_day_class", length(varlist_count)) mlist_region <- c("hosp_region");
varlist_region <- c(

  "Region::North-east",
  "Region::South-east",
  "Region::West")

reflist_region <- rep("Central", length(varlist_region)); mlist0_region <-
rep("hosp_region", length(varlist_region))


mlist_racecount <- c(paste0("",mlist_race,"*count_day_class"));

varlist_racecount <- c(paste0("",strsplit(varlist_race,"::")[[1]][2],"/Days from January 1,2020::[0,90)"),
                       paste0("",strsplit(varlist_race,"::")[[1]][2],"/Days from January 1,2020::[90,120)"),

                       paste0("",strsplit(varlist_race,"::")[[1]][2],"/Days from January 1,2020::[120,150)"),

                       paste0("",strsplit(varlist_race,"::")[[1]][2],"/Days from January 1,2020::[150,180)"))reflist_racecount <-
c(paste0("Caucasian/Days from January 1,2020::",symbol,"90"),

                       paste0("Caucasian/Days from January 1,2020::<90|",symbol,"120"),
                       paste0("Caucasian/Days from January 1,2020::<120|",symbol,"150"),
                       paste0("Caucasian/Days from January 1,2020::<150|",symbol,"180"))

mlist0_racecount <- rep(mlist_racecount, length(varlist_racecount))



## Fixed effects

txt <- strsplit(variable_list, ":")[[1]]

txt2 <- paste0("mlist_", tolower(txt), "", sep = "", collapse = ", ")XXX <- paste0("c(",
txt2,")")

XXX <- eval(parse(text = XXX))
```

© 2021 Asch DA et al. *JAMA Network Open*

```
variable_list_BAR <- paste0("", txt, collapse = "|")

## Delete other races except black-white

dim(Data2)
```

```
## Data for all cases

Data22 <- Data2[, colnames(Data2) %in% c(XXX, ylist_cat, "hosp_name")]Data22 <-
Data22[complete.cases(Data22), ]

dim(Data22); dim(Data2)



## If state is available (obtain full names for U.S. states)

if(state == TRUE){

library(usmap)

tr <- as.numeric(table(Data22$hosp_state))names(tr)
<- toupper(names(tr))

statecase2 <- data.frame("abbr" = as.character(toupper(sort(unique(Data22$hosp_state)))))statecase2$abbr <-
as.character(statecase2$abbr)

statecase2$full <- as.character(statepop$full)[match(statecase2$abbr, statepop$abbr)]statecase2$cases <- tr

statecase2$fips <- as.character(statepop$fips)[match(statecase2$abbr, statepop$abbr)]statecase2 <-
as_tibble(statecase2)

B1 <- as.character(toupper(Data22$hosp_state))B2 <-
rep(NA, nrow(Data22))

for(ii in 1 : length(tr)){

  i1 <- which(statecase2$abbr == statecase2$abbr[ii])name2fill <-
  statecase2$full[i1]

  B2[which(B1 == statecase2$abbr[ii])] <- name2fill

}

Data22$hosp_state_full <- B2

}


uid <- unique(Data22$hosp_name); length(uid); lid <- length(uid)



## Setting reference

chft <- function_character(Data22, variable_list_BAR, count_variable = TRUE, refh, refs)
```

© 2021 Asch DA et al. *JAMA Network Open*

```
Data22 <- chft$Data22 # Ordered by hospital name (Data2 OR GGdata is not ordered by hospital name)


fom_w_hosp <- fom_wot_hosp <- fom_rand_hosp <-   fom_w_hosp_pro <-time1
    <- time2 <- time3 <- time4 <-

    blk_glmm <- wht_glmm <- blk_glm_fx <- wht_glm_fx <- blk_glm_wot <- wht_glm_wot <- NULL


# prediction data

Data22_blk <- Data22_wht <- Data22;

if(mlist_race == "race_cd"){
```

© 2021 Asch DA et al. *JAMA Network Open*

```
    Data22_blk$race_cd <- ifelse(Data22_blk$race_cd == "caucasian", "african-american", "african-american") # all  black
    Data22_wht$race_cd <- ifelse(Data22_wht$race_cd == "african-american", "caucasian", "caucasian") # all  white
}

## Base model (GLM) - WOT fixed hospital effects
if(GLM == TRUE){
    if(state == FALSE){
        a1 <- paste0("glm(", ylist_cat," ~ ")
        a2 <- paste0("", XXX, "", collapse = " + ")
        QQ<-paste0("",a1,"",a2,",data=Data22,family=binomial(link='logit'))")# remove NAs by default
        time1 <- system.time(fom_wot_hosp <- try(eval(parse(text = QQ))))[3]

        blk_glm_wot <- predict.glm(fom_wot_hosp, newdata = Data22_blk,
                                   type = "link", se.fit = TRUE) wht_glm_wot <-
        predict.glm(fom_wot_hosp, newdata = Data22_wht,
                                   type = "link", se.fit = TRUE)

    }else  if(state == TRUE){
        a1 <- paste0("glm(", ylist_cat," ~ hosp_state_full + ")a2 <- paste0("",
        XXX, "", collapse = " + ")
        QQ<-paste0("",a1,"",a2,",data=Data22,family=binomial(link='logit'))")# remove NAs by default
        time1 <- system.time(fom_wot_hosp <- try(eval(parse(text = QQ))))[3]

        blk_glm_wot <- predict.glm(fom_wot_hosp, newdata = Data22_blk,
                                   type = "link", se.fit = TRUE) wht_glm_wot <-
        predict.glm(fom_wot_hosp, newdata = Data22_wht,
                                   type = "link", se.fit = TRUE)
    }
    summary(fom_wot_hosp)
```

© 2021 Asch DA et al. *JAMA Network Open*

```
}

## Base model (GLM) - With fixed hospital effects + Profile Data22$mbr_composite_num
<- ifelse(Data22$mbr_composite_flag == "Alive", 0, 1)if(ylist_cat != "mbr_composite_flag"){

    try(Data22$mbr_death_num <- ifelse(Data22$mbr_death_flag == "Alive", 0, 1))

    try(Data22$mbr_hospice_num <- ifelse(Data22$mbr_hospice_flag == "Alive", 0, 1))

}

if(GLM_ML == TRUE){

    a1 <- paste0("glmmboot(", ylist_num," ~ ")
```

© 2021 Asch DA et al. *JAMA Network Open*

```
a2 <- paste0("", XXX, "", collapse = " + ")
if(bootH == TRUE){
  QQ <- paste0("", a1, "", a2, ", cluster = hosp_name, family = binomial, boot = bootH, data = Data22)") # remove NAs bydefault
  time2 <- system.time(fom_w_hosp_pro <- try(eval(parse(text = QQ))))[3]
}else if(bootH==FALSE){
  QQ <- paste0("", a1, "", a2, ", cluster = hosp_name, family = binomial, boot = 0, data = Data22") # remove NAs by def
ault
  time2 <- system.time(fom_w_hosp_pro <- try(eval(parse(text = QQ))))[3]
}
}

## Base model (GLM) - With fixed hospital effects
if(GLM_FX == TRUE){
  a1 <- paste0("glm(", ylist_cat," ~ hosp_name + ")a2 <-
  paste0("", XXX, "", collapse = " + ")
  QQ<-paste0("",a1,"",a2,",data=Data22,family=binomial(link='logit'))")# remove NAs by default
  time3 <- system.time(fom_w_hosp <- try(eval(parse(text = QQ))))[3]

  blk_glm_fx <- predict.glm(fom_w_hosp, newdata = Data22_blk,
                            se.fit = TRUE, type = "link") wht_glm_fx <-
  predict.glm(fom_w_hosp, newdata = Data22_wht,
                            se.fit = TRUE, type = "link")
}

## Base model GLMM - with random hospital effects
if(GLMM_RE == TRUE){
  if(laplace == TRUE){
```

© 2021 Asch DA et al. *JAMA Network Open*

```
a1 <- paste0("glmmTMB(", ylist_cat," ~ ", ranlist, " + ")a2 <- paste0("",
XXX, "", collapse = " + ")

QQ <- paste0("", a1, "", a2, ", REML = reml, data = Data22, family = binomial)") # remove NAs by default

time4 <- system.time(fom_rand_hosp <- try(eval(parse(text = QQ))))[3]


blk_glmm <- predict(fom_rand_hosp, newdata = Data22_blk,

                    se.fit = TRUE, type = "link") wht_glmm <-
predict(fom_rand_hosp, newdata = Data22_wht,

                    se.fit = TRUE, type = "link")

} else if(laplace == FALSE){

  # Get the parenthesis and what is inside

  ranlist <- substring(ranlist, 2, nchar(ranlist) - 1) # extract within parenthesis
```

© 2021 Asch DA et al. *JAMA Network Open*

```
            a1 <- paste0("", ylist_cat," ~ ")

            a2 <- paste0("", XXX, "", collapse = " + ")

            QQ <- paste0("GLMMadaptive::mixed_model(fixed = ", a1, "", a2, ", random = ~", ranlist, ",data = Data22, family =
                        binomial(), control = list(nAGQ = 11))")

            time4 <- system.time(fom_rand_hosp <- try(eval(parse(text = QQ))))[3]


            blk_glmm <- predict(fom_rand_hosp, newdata = Data22_blk, newdata2 = NULL,type_pred =
                                    c("link"), se.fit = TRUE,

                                    type = c("subject_specific"))

            wht_glmm <- predict(fom_rand_hosp, newdata = Data22_wht, newdata2 = NULL,type_pred
                                    = c("link"), se.fit = TRUE,

                                    type = c("subject_specific"))

        }

    }


    output = list(fom_w_hosp = fom_w_hosp,

                  fom_wot_hosp = fom_wot_hosp,
                  fom_rand_hosp = fom_rand_hosp,
                  fom_w_hosp_pro = fom_w_hosp_pro,
                  time1 = time1, time2 = time2, time3 =
                  time3, time4 = time4,

                  blk_glmm = blk_glmm, wht_glmm = wht_glmm, blk_glm_fx
                  = blk_glm_fx, wht_glm_fx = wht_glm_fx,

                  blk_glm_wot = blk_glm_wot, wht_glm_wot = wht_glm_wot,Data22
                  = Data22)

}



# A7. Plot

plotOR_blm <- function(fit,

                       fit_type = "glmmML",   mlist_race = "race_cd",
                       varlist_race = "Race::african-american",

                       reflist_race = "White", comorb_name = list(comorb_nm, comorb_id), ylist =
                       c("mbr_composite_flag", "mbr_hospice_flag", "mbr_death_flag"),resp_type = "death",
```

© 2021 Asch DA et al. *JAMA Network Open*

```
variable_list = "Age:Gender:Race:Nurse:Comorbidity:Count",state =
FALSE,

xl = 0.2, xu = 15,

txtcol = paste0("No. Events/\nCOVID patients"),xlab_title =
paste0("Adjusted odds ratio"), topr = 30){
```

© 2021 Asch DA et al. *JAMA Network Open*

```
symbol <- "\u2265"; symbol2 <- intToUtf8(8804)

mlist_race <- mlist_race; varlist_race <- varlist_race; reflist_race <- rep(reflist_race, length(varlist_race));mlist0_race <- rep(mlist_race,
length(varlist_race))

mlist_age <- c("age_class"); varlist_age <- c("Age::[55,65)", "Age::[65,75)", "Age::[75,85)", paste0("Age::", symbol,"85"

)); reflist_age <- rep("[18,55)", length(varlist_age)); mlist0_age <- rep("age_class", length(varlist_age))

mlist_gender <- c("gender"); varlist_gender <- c("Gender::Male"); reflist_gender <- rep("Female", length(varlist_gender));mlist0_gender <- rep("gender",
length(varlist_gender))

mlist_income <- c("mbr_income_class"); varlist_income <- c("Income::<50K", "Income::[50K,70K)");

reflist_income <- rep(paste0("", symbol,"70K"), length(varlist_income)); mlist0_income <- rep("mbr_income_class", length(varlist_income))


comorb_nm <- comorb_name[[1]]; comorb_id <- comorb_name[[2]]

mlist_comorbidity <- c(comorb_id); varlist_comorbidity <- paste0("", comorb_nm, "::Yes", sep = ""); reflist_comorbidity <-rep("No",
length(varlist_comorbidity)); mlist0_comorbidity <- comorb_id

mlist_nurse <- c("snf_nursing_flag"); varlist_nurse <- c("Transferred from nursing facility::Yes"); reflist_nurse <- rep("No", length(varlist_nurse));
mlist0_nurse <- rep("snf_nursing_flag", length(varlist_nurse))

mlist_count <- c("count_day_class"); varlist_count <- c("Days from January 1,2020::[0,90)",

                                                  "Days from January 1,2020::[90,120)","Days
                                                  from January 1,2020::[120,150)","Days from
                                                  January 1,2020::[150,180)")

reflist_count <- rep(paste0("", symbol,"180"), length(varlist_count));mlist0_count <-
rep("count_day_class", length(varlist_count)) mlist_region <- c("hosp_region");
varlist_region <- c(

 "Region::North-east",
  "Region::South-east",
  "Region::West")


reflist_region <- rep("Central", length(varlist_region)); mlist0_region <- rep("hosp_region", length(varlist_region))mlist_racecount <-


c(paste0("",mlist_race,"*count_day_class"));

varlist_racecount <- c(paste0("Race::",tolower(strsplit(varlist_race,"::")[[1]][2])," & Days::[0,90)"),

                      paste0("Race::",tolower(strsplit(varlist_race,"::")[[1]][2])," & Days::[90,120)"),

                      paste0("Race::",tolower(strsplit(varlist_race,"::")[[1]][2])," & Days::[120,150)"),
```

© 2021 Asch DA et al. *JAMA Network Open*

```
paste0("Race::",tolower(strsplit(varlist_race,"::")[[1]][2])," & Days::[150,180)"))reflist_racecount <-
rep("o/w", length(varlist_racecount))

mlist0_racecount <- rep(mlist_racecount, length(varlist_racecount))



## Fixed effects

txt <- strsplit(variable_list, ":")[[1]]
```

© 2021 Asch DA et al. *JAMA Network Open*

```
txt2 <- paste0("mlist_", tolower(txt), "", sep = "", collapse = ", ")XXX <- paste0("c(",
txt2,")")

XXX <- eval(parse(text = XXX))

variable_list_BAR <- paste0("", txt, collapse = "|")


Data22 <- fit$Data22


if(fit_type == "glmmML"){ whos
    <- fit$fom_w_hosp_pro

    orp <- whos$coefficients #$coefficients[-1,  1]

    VCV <- diag(whos$variance)

} else if(fit_type == "glm_w"){whos <-
    fit$fom_w_hosp

    orp <- whos$coefficients[-c(1, grep("hosp_name", names(whos$coefficients), value = FALSE))]#[-seq_len(lid)]VCV <-
    diag(vcov(whos))[-c(1, grep("hosp_name", names(whos$coefficients), value = FALSE))]#[-seq_len(lid)]

} else  if(fit_type == "glm_wot"){whos
    <- fit$fom_wot_hosp if(state ==
    FALSE){

        orp <- whos$coefficients[-1]VCV <-
        diag(vcov(whos))[-1]

    }else  if(state == TRUE){

        orp <- whos$coefficients[-c(1, grep("hosp_state_full", names(whos$coefficients), value = FALSE))]VCV <- diag(vcov(whos))[-
        c(1, grep("hosp_state_full", names(whos$coefficients), value = FALSE))]

    }

} else  if(fit_type == "glmmTMB"){
    whos <- fit$fom_rand_hosp

    orp <- summary(whos)$coefficients$cond[-1, 1] #$coefficients[-1,  1]

    VCV <- diag(vcov(fit$fom_rand_hosp)$cond)[-1]

    #QQ  <-  eval(parse(text  =  QQ))

} else  if(fit_type == "glmmQUAD"){
    whos <- fit$fom_rand_hosp

    orp <-   summary(whos)$coef_table[-1, 1]or <-
    exp(orp)
```

© 2021 Asch DA et al. *JAMA Network Open*

```
    VCV <- vcov(whos, sandwich = FALSE)

    VCV <- diag(VCV)[- c(1, length(diag(VCV)))]

  }
```

*## refernce: http://people.upei.ca/hstryhn/stryhn208.pdf uses profile based confidence interval inverting LRT*
*## Instead we use wald Hosmer & Lemeshow's Applied logistic regression (2nd edition) as below*

*## https://stats.stackexchange.com/questions/5304/why-is-there-a-difference-between-manually-calculating-a-logistic-regression-95*

```
or <- exp(orp) sdorp
<- sqrt(VCV)

lb <- exp(orp - 1.96 * sdorp)ub <-
exp(orp + 1.96 * sdorp)

dat <- data.frame("Variable" = names(or),

                  "bOR" =  or,

                  "lb" =  lb,

                  "ub" = ub,

                  "pval" = 2 * (1 - pnorm(abs(orp/sdorp))))


txt <- strsplit(variable_list, ":")[[1]]

## final name

txt2 <- paste0("mlist0_", tolower(txt), "", sep = "", collapse = ", ")QQ <- paste0("c(",
txt2,")")

QQ <-   eval(parse(text = QQ))



inout_ix <- unlist(lapply(seq_len(length(QQ)),   function(ii)   { inout <-

  grep(ifelse(QQ[ii] %in% "race_cd_class*count_day_class",

                         paste0("race_cd_class", Hmisc::capitalize(strsplit(varlist_race,"::")[[1]][2]), ":count_day_class"
), QQ[ii]),

                dat$Variable, value = F)


  if(length(inout) >= 1){

    1

  }else{

    9999 # missing

   }

 }))
```

```
lni <- which(inout_ix == 9999)

if(length(lni) > 0){

    dat$finalname <-   QQ[- which(inout_ix == 9999)]

}else{

    dat$finalname <- QQ

}


## ylab_name

txt2 <- paste0("varlist_", tolower(txt), "", sep = "", collapse = ", ")QQ <- paste0("c(",
txt2,")")
```

© 2021 Asch DA et al. *JAMA Network Open*

```
QQ <- eval(parse(text = QQ))

if(length(lni) > 0){
    dat$ylab_name <-   QQ[- which(inout_ix == 9999)]
}else{
    dat$ylab_name <- QQ
}
##reference
txt2 <- paste0("reflist_", tolower(txt), "", sep = "", collapse = ", ")QQ <- paste0("c(",
txt2,")")
QQ <- eval(parse(text = QQ))

if(length(lni) > 0){
    dat$reference <- QQ[- which(inout_ix == 9999)]
}else{
    dat$reference <- QQ
}
name_coef <- as.character(dat$Variable)

##Obtain  cases
ylab_name_numb <- t(do.call(cbind, lapply(seq_len(nrow(dat)), function(uu) {where <-
    dat$finalname[uu]

    if(where  != "race_cd_class*count_day_class"){
        numb <- table(Data22[, colnames(Data22) %in% where])what <-
        strsplit(name_coef[uu], where)[[1]][2]
        numb <- numb[which(names(numb) == what)]

        QQ <- subset(Data22, select = c(where,
```

```
ifelse(resp_type == "composite", ylist[1],
       ifelse(resp_type == "hospice", ylist[2], ylist[3]))))

numb_event <- length(which(QQ[, 1] == what & QQ[, 2] %in% c("Event", "Hospice", "Death"))) # 1st column = respective variable and 2nd column respective events

}else if(where == "race_cd_class*count_day_class"){

numb <- table(Data22[, colnames(Data22) %in% c("race_cd_class", "count_day_class")])

a1 <- Hmisc::capitalize(strsplit(strsplit(name_coef[uu], "race_cd_class")[[1]][2], ":")[[1]][1])a2 <- strsplit(name_coef[uu], "count_day_class")[[1]][2]

numb <- numb[which(rownames(numb) == a2), which(colnames(numb) == a1)]

where <- c("race_cd_class", "count_day_class")
```

© 2021 Asch DA et al. *JAMA Network Open*

```
            QQ <- subset(Data22, select = c(where, ifelse(resp_type == "composite", ylist[1],
                                                                                    ifelse(resp_type == "hospice",ylist[2],
                                                                                           ylist[3]))))


            numb_event <- length(which(QQ[, 1] == a1 & QQ[, 2] == a2 & QQ[, 3] %in% c("Event", "Hospice", "Death")))

        }

        as.vector(c(numb, numb_event))

    })))


    ## Merging all

    dat$ylab_name_numb <- ylab_name_numb[, 1]
    dat$ylab_name_numb_event <- ylab_name_numb[, 2]



    dat$allpoint <- unlist(lapply(seq_len(nrow(dat)), function(hh)

        paste0("",sprintf("%2.2f",round(dat$bOR[hh], 2)),"(",sprintf("%2.2f",round(dat$lb[hh], 2)),",",sprintf("%2.2f",round(dat
$ub[hh], 2)), ")")))


    dat$ylab_name_numbX = paste0("", dat$ylab_name_numb_event, "/", dat$ylab_name_numb, "")zerome <-

    which(dat$ylab_name_numbX == "0/0")

    if(length(zerome) > 0){

        dat$ylab_name_numbX[zerome] <- NA

    }


    dat$pval <- sprintf("%0.4f", dat$pval)
    dat$pval[which(dat$pval == "0.0000")] <- "<0.0001"


    # make black first

    dat <- rbind(dat[grep("Race::", dat$ylab_name, value = F), ],dat[-
                          grep("Race::", dat$ylab_name, value = F), ])
```

© 2021 Asch DA et al. *JAMA Network Open*

```
par(bty = "n", mar = c(2, 25, 1, 5), mgp = c(2, 0.5, 0), xpd = FALSE, tcl = 0.25, cex = 0.9)
#par(bty = "n", mar = c(5, 30, topr, 15),
#      mgp = c(2, 0.5, 0), xpd = FALSE, tcl = 0.25,
#      cex = 0.9)
plotOR_paper(dataplot = dat,
             xl = xl, xu = xu,title =
             "", txtcol = txtcol,
             xlab_title = xlab_title)
```

© 2021 Asch DA et al. *JAMA Network Open*

```
}


## A8. Used within plotOR_blm

plotOR_paper <- function(dataplot, xl = 0.2, xu = 5,

                         title = "Main title",

                         txtcol = paste0("No. Events/\nCOVID patients"),xlab_title =
                         "Adjusted odds ratio"){

  lc <- nrow(dataplot)revy
  <- seq_len(lc)


  plot(x = dataplot$bOR, y = rev(seq_len(lc)), xlab =
       xlab_title, ylab = '', xaxt = "n",yaxt = "n", pch = NA,
       log = "x",

       lwd = 2, cex.lab = 1.5, cex.main = 2,

       cex = 2, cex.axis = 2,xlim =
       c(xl, xu))


  atx <- sort(unique(c(xl, 0.5, 1, 2, 4, 8, xu)))axis(1, at = atx,
  labels = atx, cex.axis = 1.7)ven_tex_lb <- xl;

  ven_tex_ub <- 0.9;

  ven_tex_lb1    <-    1.1;
  ven_tex_ub1 <- xu


  ven_tex <- nrow(dataplot) + 1.5 #45;


  RR <- as.character(dataplot$reference)
  dataplot$reference <- RR


  # Var names

  mtext(side = 2, StrAlign(dataplot$ylab_name, sep = "\\r"),at = rev(revy),
        line = 7, las = 2,
```

© 2021 Asch DA et al. *JAMA Network Open*

```
        cex = 1, font = 1)

# Reference names

mtext(side = 2, StrAlign(dataplot$reference, sep = "\\r"), at = rev(revy), las = 1, cex = 1,font = 1, line = ifelse(txtcol ==
        "No. Events/\nCOVID patients", 2, 1), col = "black")


# CI values
```

© 2021 Asch DA et al. *JAMA Network Open*

```
mtext(side = 4, StrAlign(dataplot$allpoint, sep = "\\r"),at = rev(revy),
      las = 2, cex = 0.8, font = 1)

# P values
col_pv <- ifelse(dataplot$pval <= 0.05, "orange",
                 ifelse(dataplot$pval > 0.05 & dataplot$pval <= 0.10, "purple", "black"))

mtext(side = 4, StrAlign(as.character(dataplot$pval), sep = "\\r"),at = rev(revy), col =
      "black",
      las = 2, line = 7, cex = 0.8, font = 2)

# Events
mtext(side = 2, dataplot$ylab_name_numbX, at = rev(revy), las = 2,line = 25, cex =
      0.8, font = 1, col = "black")

# Column names
mtext(side = 2, txtcol, at = (ven_tex + 1),line = 23, las =
      1, cex = 1,
      font = 2, col = "black")

mtext(side = 2, paste0("Characteristic better"), at = (ven_tex + 1),line = -9, #-34
      (side = 4),
      las = 1, cex = 0.8, lwd = 1.2,
      font = 3,
      col = "black")

mtext(side = 2, paste0("Characteristic worse"), at = (ven_tex + 1),line = -20,# in side 4 line
      = -22,
      las = 1, cex = 0.8, lwd = 1.2,font = 3, col
      = "black")
```

© 2021 Asch DA et al. *JAMA Network Open*

```
mtext(side = 4, title, at = (ven_tex + 2),line = -43,
      las = 1, cex = 1.5, lwd = 1.2,font = 21,
      col = "black")



mtext(side = 2, paste0("Reference"), at = (ven_tex + 1),line = 1, #3,
```

© 2021 Asch DA et al. *JAMA Network Open*

```
las = 1, cex = 1, lwd = 1.2,font = 2,
col = "black")


mtext(side = 2, paste0("Characteristic"), at = (ven_tex + 1),line = 9,

las = 1, cex = 1, lwd = 1.2,font = 2,
col = "black")


mtext(side = 4, paste0("Estimate (95% CI)"),at =
(ven_tex + 1),

line = -1,

las = 1, cex = 1, lwd = 1.2,font = 2,
col = "black")


mtext(side = 4, paste0("p-value"),at =
(ven_tex + 1),

line = 7,

las = 1, cex = 1, lwd = 1.2,font = 2,
col = "black")



segments(x0 = xl, x1 = xu, y0 = revy, y1 = revy,col =
"#EEEEEE", lty = 1, lwd = 2)

segments(x0 = 1, x1 = 1, y0 = 0, y1 = tail(revy, 1) + 1,col = "black", lty = 3,
lwd = 2) #  2  DASHED


segments(round(dataplot$lb, 2),

rev(revy), round(dataplot$ub, 2),
rev(revy),

col = "black", lty = 1, lwd = 2)


arrows(ven_tex_lb, (ven_tex -

ifelse(txtcol == "No. Events/\nCOVID patients", 0.7, 1)),ven_tex_ub,
(ven_tex -

ifelse(txtcol == "No. Events/\nCOVID patients", 0.7, 1)), col = "black",lwd = 1, code = 1, lty =
5, length = 0.10)
```

© 2021 Asch DA et al. *JAMA Network Open*

```
arrows(ven_tex_lb1, (ven_tex -
                              ifelse(txtcol == "No. Events/\nCOVID patients", 0.7, 1)),ven_tex_ub1,
       (ven_tex -
                              ifelse(txtcol == "No. Events/\nCOVID patients", 0.7, 1)), col = "black",lwd = 1, code = 2, lty =
       5, length = 0.10)
```

© 2021 Asch DA et al. *JAMA Network Open*

```
    points(dataplot$bOR,  rev(seq_len(lc)),xlab = '',
              lwd = 2,

              col = "black",cex
              = 1.5,

              pch = 3)

}
```

## A9. Recycled prediction

```
SM_FIT <- function(fitr, fixed = TRUE, hospital = FALSE, mu1 = -1){


    if(fixed == TRUE){

       if(hospital == FALSE){

          cf <- exp(fitr$fom_wot_hosp$coefficients) racef <-
          cf[grep("race", names(cf), value = F)]


          pfit_blk <- fitr$blk_glm_wot$fitpfit_wht
          <- fitr$wht_glm_wot$fit


          pfit_blk_se <- fitr$blk_glm_wot$se.fit
          pfit_wht_se <- fitr$wht_glm_wot$se.fit


          smm_blk <- summary(fitr$blk_glm_wot$fit);
          smm_wht <- summary(fitr$wht_glm_wot$fit);


          lb_thet_wht <- pfit_wht - (1.96 * pfit_wht_se)
          ub_thet_wht <- pfit_wht + (1.96 * pfit_wht_se)


          lb_thet_blk <- pfit_blk - (1.96 * pfit_blk_se)ub_thet_blk <-
          pfit_blk + (1.96 * pfit_blk_se)


          pred_blk <- fitr$fom_wot_hosp$family$linkinv(pfit_blk) pred_lb_blk <-
          fitr$fom_wot_hosp$family$linkinv(lb_thet_blk)pred_ub_blk <-
          fitr$fom_wot_hosp$family$linkinv(ub_thet_blk)
```

© 2021 Asch DA et al. *JAMA Network Open*

```
pred_wht <- fitr$fom_wot_hosp$family$linkinv(pfit_wht) pred_lb_wht <-
fitr$fom_wot_hosp$family$linkinv(lb_thet_wht)pred_ub_wht <-
fitr$fom_wot_hosp$family$linkinv(ub_thet_wht)
```

© 2021 Asch DA et al. *JAMA Network Open*

```
#tst <-  try(t.test(x  =  as.numeric(fitr$wht_glm_wot$fit),
#                     y = as.numeric(fitr$blk_glm_wot$fit),
#                     mu =   mu1,
#                     alternative  =  "less",
#                     paired =  TRUE,
#                     var.equal  =  FALSE))

# Black
lb_blk_naive <- smm_blk[names(smm_blk) %in% "Mean"] - (1.96 *
                                      sd(fitr$blk_glm_wot$fit) / sqrt(nrow(fitr$Data22)))ub_blk_naive
<- smm_blk[names(smm_blk) %in% "Mean"] + (1.96 *
                                      sd(fitr$blk_glm_wot$fit) / sqrt(nrow(fitr$Data22)))

# White
lb_wht_naive <- smm_wht[names(smm_wht) %in% "Mean"] - (1.96 *
                                      sd(fitr$wht_glm_wot$fit) / sqrt(nrow(fitr$Data22)))
ub_wht_naive <- smm_wht[names(smm_wht) %in% "Mean"] + (1.96 *
                                      sd(fitr$wht_glm_wot$fit) / sqrt(nrow(fitr$Data22)))

} else if(hospital == TRUE){

cf <- exp(fitr$fom_w_hosp$coefficients)
racef <- cf[grep("race", names(cf), value = F)]racef

pfit_blk <- fitr$blk_glm_fx$fitpfit_wht
<- fitr$wht_glm_fx$fit

pfit_blk_se <- fitr$blk_glm_fx$se.fitpfit_wht_se
<- fitr$wht_glm_fx$se.fit
```

© 2021 Asch DA et al. *JAMA Network Open*

```
smm_blk <- summary(fitr$blk_glm_fx$fit);
smm_wht <- summary(fitr$wht_glm_fx$fit);


lb_thet_wht <- pfit_wht - (1.96 * pfit_wht_se)
ub_thet_wht <- pfit_wht + (1.96 * pfit_wht_se)


lb_thet_blk <- pfit_blk - (1.96 * pfit_blk_se)ub_thet_blk <-
pfit_blk + (1.96 * pfit_blk_se)
```

© 2021 Asch DA et al. *JAMA Network Open*

```
pred_blk <- fitr$fom_w_hosp$family$linkinv(pfit_blk) pred_lb_blk <-
fitr$fom_w_hosp$family$linkinv(lb_thet_blk)pred_ub_blk <-
fitr$fom_w_hosp$family$linkinv(ub_thet_blk)


pred_wht <- fitr$fom_w_hosp$family$linkinv(pfit_wht) pred_lb_wht <-
fitr$fom_w_hosp$family$linkinv(lb_thet_wht)pred_ub_wht <-
fitr$fom_w_hosp$family$linkinv(ub_thet_wht)



#tst <-  try(t.test(x  =  fitr$wht_glm_fx$fit,
#                         y = fitr$blk_glm_fx$fit,
#                         mu =   mu1,
#                         alternative  =  "less",
#                         paired =  TRUE,
#                         var.equal  =  FALSE))


# Black

lb_blk_naive <- smm_blk[names(smm_blk) %in% "Mean"] - (1.96 *
                                              (sd(fitr$blk_glm_fx$fit) / sqrt(nrow(fitr$Data22))))ub_blk_naive
<- smm_blk[names(smm_blk) %in% "Mean"] + (1.96 *
                                              (sd(fitr$blk_glm_fx$fit) / sqrt(nrow(fitr$Data22))))


# White

lb_wht_naive <- smm_wht[names(smm_wht) %in% "Mean"] - (1.96 *
                                              (sd(fitr$wht_glm_fx$fit) / sqrt(nrow(fitr$Data22))))ub_wht_naive
<- smm_wht[names(smm_wht) %in% "Mean"] + (1.96 *
                                              (sd(fitr$wht_glm_fx$fit) / sqrt(nrow(fitr$Data22))))

    }

}else  if(fixed==FALSE){
```

© 2021 Asch DA et al. *JAMA Network Open*

```
cf <- exp(fitr$fom_rand_hosp$coefficients) racef <-
cf[grep("race", names(cf), value = F)]

pfit_blk <- fitr$blk_glmm$pred
pfit_wht <- fitr$wht_glmm$pred

pfit_blk_se <- fitr$blk_glmm$se.fit
pfit_wht_se <- fitr$wht_glmm$se.fit

smm_blk <- summary(fitr$blk_glmm$pred);
```

© 2021 Asch DA et al. *JAMA Network Open*

```
smm_wht <- summary(fitr$wht_glmm$pred);

lb_thet_wht <- pfit_wht - (1.96 * pfit_wht_se)
ub_thet_wht <- pfit_wht + (1.96 * pfit_wht_se)

lb_thet_blk <- pfit_blk - (1.96 * pfit_blk_se)ub_thet_blk <-
pfit_blk + (1.96 * pfit_blk_se)

pred_blk <- fitr$fom_rand_hosp$family$linkinv(pfit_blk) pred_lb_blk <-
fitr$fom_rand_hosp$family$linkinv(lb_thet_blk)pred_ub_blk <-
fitr$fom_rand_hosp$family$linkinv(ub_thet_blk)

pred_wht <- fitr$fom_rand_hosp$family$linkinv(pfit_wht) pred_lb_wht <-
fitr$fom_rand_hosp$family$linkinv(lb_thet_wht)pred_ub_wht <-
fitr$fom_rand_hosp$family$linkinv(ub_thet_wht)

# tst <- try(t.test(x = fitr$wht_glmm$pred,
#                   y = fitr$blk_glmm$pred,
#                   mu = mu1,
#                   alternative = "less",
#                   paired = TRUE,
#                   var.equal = FALSE))

# Black
lb_blk_naive <- smm_blk[names(smm_blk) %in% "Mean"] - (1.96 *
                                                       sd(fitr$blk_glmm$pred) / sqrt(nrow(fitr$Data22)))
ub_blk_naive <- smm_blk[names(smm_blk) %in% "Mean"] + (1.96 *
                                                       sd(fitr$blk_glmm$pred) / sqrt(nrow(fitr$Data22)))

# White
lb_wht_naive <- smm_wht[names(smm_wht) %in% "Mean"] - (1.96 *
                                                       sd(fitr$wht_glmm$pred) / sqrt(nrow(fitr$Data22)))
ub_wht_naive <- smm_wht[names(smm_wht) %in% "Mean"] + (1.96 *
```

© 2021 Asch DA et al. *JAMA Network Open*

```
                                                  sd(fitr$wht_glmm$pred) / sqrt(nrow(fitr$Data22)))
}

tst <- NULL
output = list(racef = racef,
              # link
              pfit_blk = pfit_blk, pfit_wht = pfit_wht,
              pfit_blk_se = pfit_blk_se, pfit_wht_se = pfit_wht_se,
```

© 2021 Asch DA et al. *JAMA Network Open*

```
# Naive about link

lb_blk_naive = lb_blk_naive, ub_blk_naive = ub_blk_naive,lb_wht_naive
= lb_wht_naive, ub_wht_naive = ub_wht_naive,

# Transformed response

pred_blk = pred_blk, pred_lb_blk = pred_lb_blk, pred_ub_blk = pred_ub_blk,pred_wht =
pred_wht, pred_lb_wht = pred_lb_wht, pred_ub_wht = pred_ub_wht,

# Link summary

smm_blk = smm_blk, smm_wht = smm_wht, tst = tst)

}
```

```
## A10. Internal function in Hmisc package for somer's C /Dxy statistic

.somers2 = function (x, y, weights = NULL, normwt = FALSE, na.rm = TRUE)

{

  if (length(y) != length(x))

    stop("y must have same length as x")y <-
  as.integer(y)

  wtpres <- length(weights)

  if (wtpres && (wtpres != length(x)))

    stop("weights must have same length as x")

  if (na.rm){

    miss <- if (wtpres) is.na(x +
      y + weights)

    else is.na(x + y)nmiss
    <- sum(miss)if
    (nmiss > 0) {

      miss <- !missx <-
      x[miss]y <-
      y[miss]if
      (wtpres)
```

© 2021 Asch DA et al. *JAMA Network Open*

```
            weights <- weights[miss]
    }
}
else nmiss <- 0
u <- sort(unique(y))
if (any(!(y %in% 0:1)))
    stop("y must be binary")
if (wtpres){
```

© 2021 Asch DA et al. *JAMA Network Open*

```
    if (normwt)

        weights <- length(x) * weights/sum(weights)n <-
    sum(weights)

  }

  else  n <- length(x)

  if  (n < 2)

    stop("must have >=2 non-missing observations")n1 <- if
  (wtpres)

    sum(weights[y == 1])

  else sum(y == 1)

  if  (n1 == 0 | | n1 == n)

    return(c(C = NA, Dxy = NA, n = n, Missing = nmiss))mean.rank
  <- if  (wtpres){

    require(Hmisc,quietly=TRUE)

    wtd.mean(wtd.rank(x, weights, na.rm = FALSE), weights *y)

  }else  mean(rank(x)[y == 1])

  c.index <- (mean.rank - (n1 + 1)/2)/(n - n1)dxy <- 2 *
  (c.index - 0.5)

  r <- c(c.index, dxy, n, nmiss)

  names(r) <- c("C", "Dxy", "n", "Missing")r

}


## A11. Hypothetically each patient being treated in each and every hospital

blm_model_hypothetical <- function(variable_list = "Age:Gender:Race:Nurse:Comorbidity:Count:Region:RaceCount",

                                   Data2, comorb_name = list(comorb_nm, comorb_id),

                                   ylist = c("mbr_composite_flag", "mbr_hospice_flag", "mbr_death_flag"),ylist_cat =
                                   "mbr_composite_flag",

                                   reml = FALSE,
                                   maxnum = 2,

                                   ranlist = "(1 | hosp_name)",refh =
                                   "xxx",
```

© 2021 Asch DA et al. *JAMA Network Open*

```
refs = NULL,
mlist_race = "race_cd",
varlist_race = "Race::african-american",addsize = TRUE, laplace = FALSE) {

symbol <- "\u2265"; symbol2 <- intToUtf8(8804)

mlist_race <- mlist_race; varlist_race <- varlist_race; reflist_race <- rep("Caucasian", length(varlist_race));
```

© 2021 Asch DA et al. *JAMA Network Open*

```
mlist0_race <- rep(mlist_race, length(varlist_race))

mlist_age <- c("age_class"); varlist_age <- c("Age::[55,65)", "Age::[65,75)", "Age::[75,85)", paste0("Age::", symbol,"85"
)); reflist_age <- rep("[18,55)", length(varlist_age)); mlist0_age <- rep("age_class", length(varlist_age))

mlist_gender <- c("gender"); varlist_gender <- c("Gender::Male"); reflist_gender <- rep("Female", length(varlist_gender));mlist0_gender <- rep("gender",
length(varlist_gender))

mlist_income <- c("mbr_income_class"); varlist_income <- c("Income::<50K", "Income::[50K,70K)"); reflist_income <- rep(paste0("Income::", symbol,"70K"),
length(varlist_income)); mlist0_income <- rep("mbr_income_class", length(varlist_income))

comorb_nm <- comorb_name[[1]]; comorb_id <- comorb_name[[2]]

mlist_comorbidity <- c(comorb_id); varlist_comorbidity <- paste0("", comorb_nm, "::Yes", sep = ""); reflist_comorbidity <-rep("No",
length(varlist_comorbidity)); mlist0_comorbidity <- comorb_id

mlist_nurse <- c("snf_nursing_flag"); varlist_nurse <- c("Transferred from nursing facility::Yes"); reflist_nurse <- rep("No", length(varlist_nurse));
mlist0_nurse <- rep("snf_nursing_flag", length(varlist_nurse))

mlist_count <- c("count_day_class"); varlist_count <- c("Days from January 1,2020::[0,90)",
                                                      "Days from January 1,2020::[90,120)","Days
                                                      from January 1,2020::[120,150)","Days from
                                                      January 1,2020::[150,180)")

reflist_count <- rep(paste0("", symbol,"180"), length(varlist_count));mlist0_count <-
rep("count_day_class", length(varlist_count)) mlist_region <- c("hosp_region");
varlist_region <- c(

  "Region::North-east",
  "Region::South-east",
  "Region::West")

reflist_region <- rep("Central", length(varlist_region)); mlist0_region <- rep("hosp_region", length(varlist_region))



mlist_racecount <- c(paste0("",mlist_race,"*count_day_class"));

varlist_racecount <- c(paste0("",strsplit(varlist_race,"::")[[1]][2],"/Days from January 1,2020::[0,90)"),
                       paste0("",strsplit(varlist_race,"::")[[1]][2],"/Days from January 1,2020::[90,120)"),

                       paste0("",strsplit(varlist_race,"::")[[1]][2],"/Days from January 1,2020::[120,150)"),

                       paste0("",strsplit(varlist_race,"::")[[1]][2],"/Days from January 1,2020::[150,180)"))reflist_racecount <-
c(paste0("Caucasian/Days from January 1,2020::",symbol,"90"),

                       paste0("Caucasian/Days from January 1,2020::<90|",symbol,"120"),
                       paste0("Caucasian/Days from January 1,2020::<120|",symbol,"150"),
                       paste0("Caucasian/Days from January 1,2020::<150|",symbol,"180"))

mlist0_racecount <- rep(mlist_racecount, length(varlist_racecount))
```

© 2021 Asch DA et al. *JAMA Network Open*

```
## Fixed effects

txt <- strsplit(variable_list, ":")[[1]]

txt2 <- paste0("mlist_", tolower(txt), "", sep = "", collapse = ", ")XXX <- paste0("c(",
txt2,")")
```

© 2021 Asch DA et al. *JAMA Network Open*

```
XXX <- eval(parse(text = XXX))

variable_list_BAR <- paste0("", txt, collapse = "|")


ht <- aggregate(Data2$hosp_name, by = list(Data2$hosp_name), length)select_hosp
<- ht$Group.1[which(ht$x >= maxnum)]

Data2 <- Data2[Data2$hosp_name %in% select_hosp, ]


## Data for all cases

Data22 <- Data2[, colnames(Data2) %in% c(XXX, ylist, "hosp_name", "hosp_state",
                                          "hosp_region", "bed", mlist_race)]Data22
<- Data22[complete.cases(Data22), ]

dim(Data22); dim(Data2)


uid <- unique(Data22$hosp_name); length(uid); lid <- length(uid)


## Setting reference

chft <- function_character(Data22, variable_list_BAR, count_variable = TRUE, refh, refs)

Data22 <- chft$Data22 # Ordered by hospital name (Data2 OR GGdata is not ordered by hospital name)


## Base model GLMM - with random hospital effects

if(laplace == TRUE){
  a1 <- paste0("glmmTMB(", ylist_cat," ~ ", ranlist, " + ")a2 <- paste0("",
  XXX, "", collapse = " + ")
  if(addsize == FALSE){
    QQ <- paste0("", a1, "", a2, ", data = Data22, family = binomial(link = 'logit'))") # remove NAs by default
  }else if(addsize == TRUE){
    QQ <- paste0("", a1, "", a2, " + bed, REML = reml, data = Data22, family = binomial(link = 'logit'))") # remove NAs bydefault
  }
  #QQ <- paste0("", a1, "", a2, ", REML = reml, data = Data22, family = binomial)") # remove NAs by default
```

```
time4 <- system.time(fom_rand_hosp <- try(eval(parse(text = QQ))))[3]

} else if(laplace == FALSE){
  # Get the parenthesis and what is inside
  # Remove parenthesis
  ranlist <- substring(ranlist, 2, nchar(ranlist) - 1) # extract within parenthesis
  a1 <- paste0("", ylist_cat," ~ ")
  a2 <- paste0("", XXX, "", collapse = " + ")
  if(addsize == FALSE){
    QQ <- paste0("GLMMadaptive::mixed_model(fixed = ", a1, "", a2, ", random = ~", ranlist, ",data = Data22, family =
                      binomial(), control = list(nAGQ = 11))")
```

© 2021 Asch DA et al. *JAMA Network Open*

```
    }else if(addsize==TRUE){

        QQ <- paste0("GLMMadaptive::mixed_model(fixed = ", a1, "", a2, " + bed, random = ~", ranlist, ",data = Data22, family =
                binomial(), control = list(nAGQ = 11))")

    }

    time4 <- system.time(fom_rand_hosp <- try(eval(parse(text = QQ))))[3]

}

try(rm(QQ))

a1 <- paste0("glm(", ylist_cat," ~ ")

a2 <- paste0("", XXX, "", collapse = " + ")

if(addsize == FALSE){

    QQ<-paste0("",a1,"",a2,",data=Data22,family=binomial(link='logit'))")# remove NAs by default

}else if(addsize==TRUE){

    QQ<-paste0("",a1,"",a2,"+bed,data=Data22,family=binomial(link='logit'))")# remove NAs by default

}

fom<-try(eval(parse(text=QQ)))



## Testing variance (RE)

x2 <- as.numeric(-2 * logLik(fom) + 2 * logLik(fom_rand_hosp))LRT_re <- 0.5 *
(1 - pchisq(x2, df = 1))


## AIC

aic_null <- AIC(logLik(fom))

aic_alt <- AIC(logLik(fom_rand_hosp))


bic_null <- BIC(fom)

bic_alt <- BIC(fom_rand_hosp)
```

© 2021 Asch DA et al. *JAMA Network Open*

```
if(laplace == TRUE){
  ## Correlation test
  cor_lme_lmer <- cor.test(coef(fom), fixef(fom_rand_hosp)$cond) ## Assessing model performance (conditional/marginal R2)
  SS<-MuMIn::r.squaredGLMM(fom_rand_hosp) ## confidence intervals for the variances of the random intercepts
  varint <- confint(fom_rand_hosp)[-c(1 : length(fixef(fom_rand_hosp)$cond)), ]VIFfx <- NULL
} else if(laplace == FALSE){
  ## Correlation test
  cor_lme_lmer <- cor.test(coef(fom), fixef(fom_rand_hosp))SS <-
  r2_nakagawa(fom_rand_hosp, by_group = FALSE)
  varint <- confint(fom_rand_hosp, parm = "var-cov", level = 0.95)VIFfx <-
  GLMMadaptive::VIF(fom_rand_hosp, type = c("fixed"))
}
```

© 2021 Asch DA et al. *JAMA Network Open*

```
## C and Dxy: correlation between predicted probabilities and observed responses

if(ylist_cat == "mbr_death_flag"){

  nY <- ifelse(Data22$mbr_death_flag == "Event", 1, 0)

}else if(ylist_cat == "mbr_composite_flag"){

  nY <- ifelse(Data22$mbr_composite_flag == "Event", 1, 0)

}

probs <- binomial()$linkinv(predict(fom_rand_hosp))rank_cor <-
.somers2(probs, as.numeric(nY))


## Estimating scores for being black/white

if(laplace == TRUE){

  XFOM <- getME(fom_rand_hosp, "X")
  ZFOM <- getME(fom_rand_hosp, "Z")

  FIXFOM <- fixef(fom_rand_hosp)$cond #getME(fom3, "fixef")

  RANFOM <- ranef(fom_rand_hosp)$cond$hosp_name


  LP <- XFOM %*% FIXFOM + ZFOM %*% RANFOM$`(Intercept)`FLP
  <- XFOM %*% FIXFOM


} else if(laplace == FALSE){

  XFOM <- model.matrix(fom_rand_hosp, type = "fixed")

  ZFOM  <- fastDummies::dummy_cols(Data22$hosp_name)[, -1];FIXFOM
  <- fixef(fom_rand_hosp)

  RANFOM <- ranef(fom_rand_hosp)

  LP <- XFOM %*% FIXFOM + as.matrix(ZFOM) %*% RANFOMFLP
  <- XFOM %*% FIXFOM

}


uid <- rownames(RANFOM) # Updating random effects name by the same order of Data22
```

© 2021 Asch DA et al. *JAMA Network Open*

```
if(ranlist == "(1 | hosp_name)" | ranlist == "1 | hosp_name"){pred <-
  as.vector(exp(LP) / (1 + exp(LP)))
  estm <- as.vector(exp(FLP) / (1 + exp(FLP)))

  ## Step 1: Black-vs-White probability distribution

  ## Method 2: Jeff Silber et al.

  if(length(grep("Race", variable_list_BAR, value = T)) == 1){
```

© 2021 Asch DA et al. *JAMA Network Open*

```
bigMat_noncauc <- do.call(cbind, lapply(seq_len(length(uid)), function(ll){

  if(addsize == FALSE){
    # first part is specific-to-patient and second part (hypothetically) for each one of H hospitals
    if(laplace == TRUE){
      QQ <- XFOM[, colnames(XFOM) %!in% grep(mlist_race, colnames(XFOM), value = F)] %*% FIXFOM[names(FIXFOM) %!in%
                                                                                            grep(mlist_race, col
names(XFOM), value = F)] + # RE if subject belongs to this hospital
        RANFOM$`(Intercept)`[which(rownames(RANFOM) == uid[ll])] # logit()

      QQ1 <- paste0("1 * FIXFOM[names(FIXFOM) %in% '", grep(mlist_race, colnames(XFOM), value = T)[1],"']", collapse = " + ") # race as if subject is nonwhite
      QQ1 <- eval(parse(text = QQ1))QQ +
      QQ1

    } else if(laplace == FALSE){
      QQ <- XFOM[, colnames(XFOM) %!in% grep(mlist_race, colnames(XFOM), value = F)] %*% FIXFOM[names(FIXFOM) %!in%
                                                                                            grep(mlist_race, col
names(XFOM), value = F)] + # RE if subject belongs to this hospital
        RANFOM[which(rownames(RANFOM) == uid[ll])] # logit()

      QQ1 <- paste0("1 * FIXFOM[names(FIXFOM) %in% '", grep(mlist_race, colnames(XFOM), value = T)[1],"']", collapse = " + ") # race as if subject is nonwhite
      QQ1 <- eval(parse(text = QQ1))QQ +
      QQ1
    }

  } else if(addsize == TRUE){
    # first part is specific-to-patient and second part (hypothetically) for each one of H hospitals
    if(laplace == TRUE){
M) %!in%
```

© 2021 Asch DA et al. *JAMA Network Open*

```
QQ <-        %!in% c(grep(mlist_race, colnames(XFOM), value = F), "bed")] %*% FIXFOM[names(FIXFO
XFOM[,
colname                                                                          c(grep(mli
s(XFOM)
st_race, colnames(XFOM), value = F), "bed")] + # size changes for different hospital

      RANFOM$`(Intercept)`[which(rownames(RANFOM) == uid[ll])] +
      # RE if subject belongs to this hospital


      Data22$bed[which(Data22$hosp_name == uid[ll])][1] * FIXFOM[names(FIXFOM) %in% "bed"]
    # size if subject belongs to this hospital


    QQ1 <- paste0("1 * FIXFOM[names(FIXFOM) %in% '", grep(mlist_race, colnames(XFOM), value = T)[1],"']", collapse =
```

```
"+")# race as if subject is nonwhite
            QQ1 <- eval(parse(text = QQ1))QQ +
            QQ1

          } else  if(laplace == FALSE){

            QQ <- XFOM[, colnames(XFOM) %!in% c(grep(mlist_race, colnames(XFOM), value = F), "bed")] %*% FIXFOM[names(FIXFO
M) %!in%
                                                                                                        c(grep(mli
st_race, colnames(XFOM), value = F), "bed")] +# size changes for different h
              RANFOM[which(rownames(RANFOM) == uid[ll])] +                    # RE if subject belongs to this hospital

              Data22$bed[which(Data22$hosp_name == uid[ll])][1] * FIXFOM[names(FIXFOM) %in% "bed"] # size if subject belongsto this hospital

            QQ1 <- paste0("1 * FIXFOM[names(FIXFOM) %in% '", grep(mlist_race, colnames(XFOM), value = T)[1],"']", collapse = " + ") # race as if subject is nonwhite
            QQ1 <- eval(parse(text = QQ1))QQ +
            QQ1
          }
        }
      }))

   bigMat_cauc <- do.call(cbind, lapply(seq_len(length(uid)), function(ll){

      if(addsize == FALSE){
        # first part is specific-to-patient and second part (hypothetically) for each one of H hospitals
        if(laplace == TRUE){
          QQ <- XFOM[, colnames(XFOM) %!in% grep(mlist_race, colnames(XFOM), value = F)] %*% FIXFOM[names(FIXFOM) %!in%
                                                                                        grep(mlist_race, col
names(XFOM), value = F)] +  # RE if subject belongs to this hospital
```

© 2021 Asch DA et al. *JAMA Network Open*

```
      RANFOM$`(Intercept)`[which(rownames(RANFOM)==uid[ll])]# logit()
    QQ

  } else if(laplace == FALSE){
    QQ <- XFOM[, colnames(XFOM) %!in% grep(mlist_race, colnames(XFOM), value = F)] %*% FIXFOM[names(FIXFOM) %!in%
                                                                                                   grep(mlist_race, col
names(XFOM), value=F)] +  # RE if subject belongs to this hospital
      RANFOM[which(rownames(RANFOM) == uid[ll])] # logit()
    QQ
  }
```

© 2021 Asch DA et al. *JAMA Network Open*

```
}else if(addsize==TRUE){
  # first part is specific-to-patient and second part (hypothetically) for each one of H hospitals
  if(laplace == TRUE){
    QQ <- XFOM[, colnames(XFOM) %!in% c(grep(mlist_race, colnames(XFOM), value = F), "bed")] %*% FIXFOM[names(FIXFOM) %!in%
                                                                                                   c(grep(mlist_race, colnames
(XFOM), value = F), "bed")] + # size changes for different hospital
      RANFOM$`(Intercept)`[which(rownames(RANFOM) == uid[ll])] +
      # RE if subject belongs to this hospital

      Data22$bed[which(Data22$hosp_name == uid[ll])][1] * FIXFOM[names(FIXFOM) %in% "bed"]
    # size if subject belongs to this hospital

    QQ

  } else if(laplace == FALSE){
    QQ <- XFOM[, colnames(XFOM) %!in% c(grep(mlist_race, colnames(XFOM), value = F), "bed")] %*% FIXFOM[names(FIXFO
M) %!in%
                                                                                                             c(grep(mli
st_race, colnames(XFOM), value = F), "bed")] + # size changes for different h
      RANFOM[which(rownames(RANFOM) == uid[ll])] +                    # RE if subject belongs to this hospital

      Data22$bed[which(Data22$hosp_name == uid[ll])][1] * FIXFOM[names(FIXFOM) %in% "bed"] # size if subject belongsto this hospital

    QQ
  }
 }
}))
```

© 2021 Asch DA et al. *JAMA Network Open*

```
}else{
  bigMat_noncauc <- bigMat_cauc <- NULL
}

## RSERs
if(ylist_cat ==   "mbr_death_flag"){tb <-
  table(Data22$mbr_death_flag)
  ybar <- as.numeric(tb[names(tb) %in% "Event"]) /
    length(Data22$mbr_death_flag)
}else if(ylist_cat == "mbr_composite_flag"){tb <-
  table(Data22$mbr_composite_flag)
  ybar <- as.numeric(tb[names(tb) %in% "Event"]) /
    length(Data22$mbr_composite_flag)
```

© 2021 Asch DA et al. *JAMA Network Open*

```
}


### Method 1: Drye et al.

score <- list()

for(ll in  1 : length(uid)){

    num <- pred[which(Data22$hosp_name == uid[ll])]den <-
    estm[which(Data22$hosp_name == uid[ll])]

    score[[ll]]<-sum(num)/sum(den)*ybar# 1/ni got cancelled out in numer and denom

}

score <- unlist(score)


### Method 2: Jeff et al.

bigMat <- do.call(cbind, lapply(seq_len(length(uid)), function(ll){


    if(addsize == FALSE){

        # first part is specific-to-patient and second part (hypothetically) for each one of H hospitals

        if(laplace == TRUE){

            XFOM %*% FIXFOM +  # RE if subject belongs to this hospital

                RANFOM$`(Intercept)`[which(rownames(RANFOM) == uid[ll])] # logit()

        } else if(laplace == FALSE){

            XFOM %*% FIXFOM +  # RE if subject belongs to this hospital

                RANFOM[which(rownames(RANFOM) == uid[ll])] # logit()

        }


    }else if(addsize == TRUE){

        if(laplace == TRUE){

            XFOM[, colnames(XFOM) %!in% "bed"] %*% FIXFOM[names(FIXFOM) %!in% "bed"] + # size changes for different h
```

© 2021 Asch DA et al. *JAMA Network Open*

```
        RANFOM$`(Intercept)`[which(rownames(RANFOM) == uid[ll])] +                    # RE if subject belongs to this hospit
al
        Data22$bed[which(Data22$hosp_name == uid[ll])][1] * FIXFOM[names(FIXFOM) %in% "bed"] # size if subject belongs t
o this hospital
      } else if(laplace == FALSE){
       XFOM[, colnames(XFOM) %!in% "bed"] %*% FIXFOM[names(FIXFOM) %!in% "bed"] + # size changes for different h
        RANFOM[which(rownames(RANFOM) == uid[ll])] +                    # RE if subject belongs to this hospital
        Data22$bed[which(Data22$hosp_name == uid[ll])][1] * FIXFOM[names(FIXFOM) %in% "bed"] # size if subject belongs t
o this hospital
      }
    }
  }))
```

© 2021 Asch DA et al. *JAMA Network Open*

```
## Unadjusted score

if(ylist_cat == "mbr_death_flag"){

    unadj_score <- unlist(lapply(seq_len(length(uid)),

                                            function(uu) {

                                                tr <- table(Data22$mbr_death_flag[Data22$hosp_name == uid[uu]])


                                                if(length(which(names(tr) %in% "Event")) > 0){
                                                    tr[names(tr) %in% "Event"] / sum(tr)

                                                } else if(length(which(names(tr) %in% "Event")) == 0){0

                                                }


                                            }))

    length(unadj_score)


    ## Volume

    I <- length(uid)

    hosp_orig_vol <- unlist(lapply(seq_len(I), function(ii) {ir1 <-
        which(as.numeric(Data22$hosp_name) == ii)

        ir2 <- table(Data22$mbr_death_flag[ir1])

        paste0("", ir2[which(names(ir2) %in% "Event")], "/", sum(ir2), "")

    }))

} else if(ylist_cat == "mbr_composite_flag"){ unadj_score <-
    unlist(lapply(seq_len(length(uid)),

                                            function(uu) {

                                                tr <- table(Data22$mbr_composite_flag[Data22$hosp_name == uid[uu]])


                                                if(length(which(names(tr) %in% "Event")) > 0){
                                                    tr[names(tr) %in% "Event"] / sum(tr)

                                                } else if(length(which(names(tr) %in% "Event")) == 0){0

                                                }
```

© 2021 Asch DA et al. *JAMA Network Open*

```
                                        }))

length(unadj_score)

## Volume

I <- length(uid)

hosp_orig_vol <- unlist(lapply(seq_len(I), function(ii) {ir1 <-
    which(as.numeric(Data22$hosp_name) == ii)

    ir2 <- table(Data22$mbr_composite_flag[ir1])
```

```
            paste0("", ir2[which(names(ir2) %in% "Event")], "/", sum(ir2), "")

          }))

        }

    }


    logitinv <- function(ff) {exp(ff) / (1 + exp(ff))}

    if(length(grep("Race", variable_list_BAR, value = T)) == 1){

      bigMat_noncauc <- apply(bigMat_noncauc, 2, logitinv) # does not matter 1 or 2

      bigMat_cauc    <-    apply(bigMat_cauc,    2,    logitinv)
      prob_noncauc    <-    apply(bigMat_noncauc,    1,    mean)
      prob_cauc <- apply(bigMat_cauc, 1, mean)

    }else{

      bigMat_noncauc <- bigMat_cauc <-   prob_noncauc <-   prob_cauc <- NULL

    }

    score_DS <- apply(bigMat, 2, logitinv)
    dim(bigMat_noncauc)

    score_DS_mean <- apply(score_DS, 2, mean)


    output = list(prob_noncauc = prob_noncauc,

                  prob_cauc = prob_cauc,
                  score_silber = score_DS_mean,
                  score_drye =   score,

                  score_DS = score_DS,
                  reference = uid,

                  cor_lme_lmer = cor_lme_lmer, SS = SS, varint = varint, VIFfx = VIFfx,LRT_re =   LRT_re,
                  aic_null = aic_null, aic_alt = aic_alt,

                  bic_null =   bic_null,   bic_alt =   bic_alt,
                  fom_rand_hosp = fom_rand_hosp,

                  Data22 = Data22)

}
```

© 2021 Asch DA et al. *JAMA Network Open*

*## A12. Counterfactual assignment to hospital for each patient*

```
counterfactual_RSER <- function(A, I, nameb, totalb, totalw, NB = 500, fitr,
                                method = "1-step",   varlist_race = "african-american") {

  scoreb <- fitr$score_DS[which(fitr$Data22$race_cd == varlist_race), ]; dim(scoreb)
  if(varlist_race == "african-american"){targetprob <-
    nameb$whiteprop
  } else if(varlist_race == "caucasian"){
    targetprob <- nameb$blackprop
```

© 2021 Asch DA et al. *JAMA Network Open*

```
}

counter_patient <- mean_counter_patient <- counter_site <- list()

# Two-methods for assignment of patients following Multinomial
if(method == "2-step"){
  set.seed(A + 4444)
  N1 <- rmultinom(n = 1, size = totalb, prob = targetprob); length(which(N1 == 0))setb <- seq_len(NB)
  if(length(which(N1 == 0)) != 0){
    # take 1 from the sites with more than 8 patients so that we have at-least 1 black
    # smaller prob leads to 0 black patients
    is0 <- which(N1 == 0)
    N1[is0] <- N1[is0] + 1

    is <- which(N1 >= 10)
    takout <- sample(is, length(is0), replace = FALSE)
    N1[takout] <- N1[takout] - 1

    if(length(which(N1 == 0)) == 0){
      print("Have at-least 1 black patient in each site")
    }
  }
  if(sum(N1) != totalb){
    stop("Not having same # of black patients")
  }
  for(bb in 1 : NB){

    iall <- seq_len(totalb)ih <-
    list();
```

© 2021 Asch DA et al. *JAMA Network Open*

```
idh <- rep(NA, length(iall)) # hypothetical hospital ID for patient-level

set.seed(1212 + A + bb)

ih[[1]] <- sample(iall, N1[1], replace = FALSE)

idh [ ih[[1]] ] <- paste0("H", 1, ""); length(ih[[1]]); N1[1];


for(jj in 2 : (I - 1)){

   set.seed(A + 1212 + jj + (1000 * bb))

   ## make it WOT replacement

   QQ <- paste0("ih[[",seq_len(jj - 1),"]]", collapse = ",")QQ <- paste0("c(",
   QQ, ")")

   QQ <- eval(parse(text = QQ))
```

© 2021 Asch DA et al. *JAMA Network Open*

```
    ih[[jj]] <- sample(iall[- QQ], N1[jj], replace = FALSE)
    idh [ ih[[jj]] ] <- rep(paste0("H", jj, ""), length(ih[[jj]]))rm(QQ)
    if(length(ih[[jj]]) != N1[jj]){
        stop("Check for", jj, "")
    }
}
idh[which(is.na(idh))] <- paste0("H", I, "")# what is left will go to last hospital
length(unique(idh))

if(sum(is.na(idh)) != 0){
    stop("Check idh")
}

## Find hypothetical prob
# patient-level
counter_patient[[bb]] <- unlist(lapply(seq_len(I), function(ii){
    r1 <- paste0("which(idh == 'H", ii, "')")r1 <-
    eval(parse(text = r1))
    scoreb[r1, ii]
}))

counter_site[[bb]] <- unlist(lapply(seq_len(I), function(ii){r1 <-
    paste0("which(idh == 'H", ii, "')")
    r1 <- eval(parse(text = r1))
    mean(scoreb[r1, ii])
}))

mean_counter_patient[[bb]]   <-  mean(counter_patient[[bb]])
```

© 2021 Asch DA et al. *JAMA Network Open*

```
} # simulation loop ends

} else if(method == "1-step"){ # 2-step ends cprob
  <- cumsum(targetprob); length(cprob)cprob1 <- c(0,
  cprob)

  nprob <- paste0("H", 1 : I, "")

  for(bb in 1:NB){# simulation over NB starts

    set.seed(A + 1212 + (1000 * bb))

    runb <- runif(n = totalb, min = 0, max = 1)
```

```
A1 <- cprob1[c(1 : length(cprob) )]

A2 <- cprob


# assignment of hospital ID by patients

idh <- unlist(lapply(seq_len(totalb), function(jj) {A0 <-
   rep(runb[jj],  length(cprob) )

   inx <- A0 >= A1 & A0 < A2 #  [lb,ub)                 check if generated point belongs to this interval

   nprob[inx]

}))


if(length(unique(idh)) != I){# situation when not a single black assigned to a hospital (need at-least 1 black)

   set.seed(5555 + bb)

   whichmis <- nprob[nprob %!in% sort(unique(idh))]xx <-
   table(sort(idh))

   ix <- names(xx)[which(as.vector(xx) >= 10)]

   toix <- sample(which(idh %in% ix), length(whichmis), replace = FALSE) shuffled_whichmis
   <- sample(whichmis, length(whichmis), replace = FALSE)
   if(length(unique(shuffled_whichmis)) != length(whichmis)){

      stop("Shuffled and original vector names mismatch.")

   }

   idh[toix] <- shuffled_whichmis

}


if(sum(is.na(idh)) != 0 | length(unique(idh)) != I){

   stop("Check id indexing calculation")

}


## Find hypothetical prob patient-level

counter_patient[[bb]] <- unlist(lapply(seq_len(I), function(ii){r1 <-
   paste0("which(idh == 'H", ii, "')")
```

© 2021 Asch DA et al. *JAMA Network Open*

```
    r1 <- eval(parse(text = r1))
    scoreb[r1, ii]

}))


counter_site[[bb]] <- unlist(lapply(seq_len(I), function(ii){r1 <-
    paste0("which(idh == 'H", ii, "')")

    r1 <- eval(parse(text = r1))
    mean(scoreb[r1, ii])

}))


mean_counter_patient[[bb]]   <-  mean(counter_patient[[bb]])
```

© 2021 Asch DA et al. *JAMA Network Open*

```
      } # bb  ends

    }# 2nd method ends for simulation


    output = list(counter_patient = counter_patient, counter_site =
                            counter_site, mean_counter_patient =
                            mean_counter_patient)

}
```

```
## A15. Find summary statistics of a vector

summary_statistics_table <- function(score2find){stat <-
    stat.desc(score2find, basic = TRUE) stat <- c(stat,
    quantile(score2find, 0.75) -

                        quantile(score2find, 0.25))


    result <- as.matrix(stat)


    rownames(result) <- c(head(rownames(result), (length(stat) - 1)), "IQR")#,



    result <- result[rownames(result) %in% c("nbr.val", "min", "max",
                                                    "median", "mean",
                                                    "SE.mean",
                                                    "var",
                                                    "IQR"), ]


    result <- as.matrix(result)

    rownames(result) <- c("Number", "Minimum", "Maximum", "Median", "Mean",
                                    "SE of mean",
                                    "Variance", "IQR")

    colnames(result) <- "Summary statistics"result <-
    as.data.frame(round(result, 3))
```

© 2021 Asch DA et al. *JAMA Network Open*

```
cimean <- paste0("(", sprintf("%0.2f", mean(score2find) - 1.96 * sd(score2find) /
        sqrt(length(score2find))), ", ",sprintf("%0.2f", mean(score2find) +

                            1.96 * sd(score2find) / sqrt(length(score2find))),")")


result$`Summary statistics`<- sprintf("%0.2f", result$`Summary statistics`)result2 <-
c(result$`Summary statistics`, cimean)

result2 <- as.matrix(result2)

rownames(result2) <- c(rownames(result), "95% CI for mean")
```

© 2021 Asch DA et al. *JAMA Network Open*

```
    colnames(result2) <- "Summary statistics"
    [illegible]
}
```

# Simulating patient outcome data

Here we simulate patient outcome data for different hospitals. Note that data is generated without considering any clinical perspectives. While the results do not mean anything intuitively, this is designed solely for illustrating the adopted methodologies to fit nested generalized linear model and perform counter-factual analysis. Consider response being a binary variable with 1 denoting patient-specific adverse event and 0 otherwise. The covariates consist of demographic, clinical, and comorbid variables.

© 2021 Asch DA et al. *JAMA Network Open*

```
names <- c("age_class", "gender", "mbr_income_class", "race_cd", "snf_nursing_flag", "aeci_aids_flag",
                    "aeci_alcohol_flag", "aeci_anemdef_flag", "aeci_arth_flag", "aeci_bldloss_flag",
                    "aeci_chf_flag", "aeci_chrnlung_flag","aeci_coag_flag", "aeci_depress_flag", "aeci_dm_flag",
                    "aeci_dmcx_flag", "aeci_drug_flag", "aeci_htn_flag", "aeci_hypothy_flag", "aeci_liver_flag",
                    "aeci_lymph_flag", "aeci_lytes_flag", "aeci_mets_flag", "aeci_neuro_flag", "aeci_obese_flag",
                    "aeci_para_flag", "aeci_perivasc_flag", "aeci_psych_flag",

                    "aeci_pulmcirc_flag", "aeci_renlfail_flag", "aeci_tumor_flag", "aeci_ulcer_flag","aeci_valve_flag",
                    "aeci_wghtloss_flag", "count_day_class", "size", "mbr_composite_flag", "hosp_name")

comorb_id <- grep("aeci_", names,   value   = T)

## Name of comorbid variables

comorb_nm <- c("Acquired immune deficiency syndrome", "alcohol abuse",

                         "Iron deficiency anemia", "Rheumatoid arthritis", "bloodloss anemia", "Congestive heart failure",
                         "Chronic obstructive pulmonary disease", "Coagulopathy", "depression", "Diabetes without
                         chronic complication", "Diabetes with chronic complication", "drug abuse", "hypertension",
                         "hypothyroidism", "liver disease", "lymphoma", "fluid/electrolyte disorder","metastatic cancer",
                         "neurological disorders", "obesity", "paralysis", "peripheral vascular disease" ,"psychosis",
                         "pulmonary circulation disorder","Chronic kidney disease", "tumor w/o metastasis", "peptic ulcer
                         disease", "valvular disorder", "Weight loss")

#  comorbidity  names

tow <- names[names %!in% c('gender', 'snf_nursing_flag', 'size', 'mbr_composite_flag','hosp_name',
          'count_day_class', 'age_class', "mbr_income_class", "race_cd")]


K <- 700 # Number of sites

dataN <- list()


set.seed(999999)

beta_com <- round(rnorm(length(tow), 0, 1.5), 2) beta_com[sample(1 :
length(beta_com), 10, replace = FALSE)] <- 0beta_int <- rnorm(1, -3, 4)

beta_age <- rnorm(1, 1, 1)

beta_nurse <- rnorm(1, -0.5, 0.50)

beta_count <- rnorm(1, 0.10, 0.50)

beta_gender <- rnorm(1, -0.50, 0.50)

beta_size <- 0
```

© 2021 Asch DA et al. *JAMA Network Open*

```
beta_race <- rnorm(1, 1.5, 1)

beta_income <- 0
```

© 2021 Asch DA et al. *JAMA Network Open*

```
c(beta_com, beta_age, beta_count,   beta_nurse, beta_size, beta_int, beta_gender, beta_race, beta_income)
```

```
## [1]   1.5400000  0.0000000  0.4100000 -0.3400000  0.9300000 -1.5100000
## [7]  -1.4800000 -2.5900000  0.0000000 -0.8800000  0.0000000  0.0000000
## [13] -1.8100000 -1.5700000  0.0000000  0.4600000  0.7100000  1.0400000
## [19] -1.8800000  0.0000000 -0.7500000  0.0000000  3.0200000  0.0000000
## [25]  0.0000000  0.5500000  0.0000000  1.2800000 -1.3900000  0.1223221
## [31]  0.4374867 -1.1553451  0.0000000 -1.0019836 -0.3439332  2.2595724
## [37]  0.0000000
```

© 2021 Asch DA et al. *JAMA Network Open*

```
# Design matrix representing binary 1/0
dataN_All <- do.call(rbind, lapply(seq_len(K), function(kk) {set.seed(777777 + kk)
  nh <- round(runif(1, 5, 120))  # number of patients in each hospital; minimum 2 and maximum 120
  dataN$hosp_name <- rep(paste0("Hospital_", kk,""), nh)


  dataN$size <- rep(rpois(1, nh / 5), nh) # 1/5th is ICU bed size
  dataN$size_scale <- log(1 + dataN$size)


  for(jj in  1 : length(names)){
    if(names[jj] == "gender"){
      set.seed(jj + 8000)
      QQ <- paste0("dataN$", names[jj], " <- rbinom(", nh, ", 1, 0.60)")QQ <-
      eval(parse(text = QQ))
    }
    if(names[jj] == "mbr_income_class"){set.seed(jj +
      30000)
      dataN$mbr_income <- round(runif(nh, min = 15000, max = 150000)) # min 15K/year to 150K/year
      dataN$mbr_income_scale <- (dataN$mbr_income - min(dataN$mbr_income)) /
                                                        (max(dataN$mbr_income) - min(dataN$mbr_income))
    }
    if(names[jj] == "race_cd"){
      set.seed(jj + 80000)
      QQ <- paste0("dataN$", names[jj], " <- rbinom(", nh, ", 1, 0.30)")QQ <-
      eval(parse(text = QQ))
    }
    if(names[jj] == "snf_nursing_flag"){set.seed(jj + 1)
      QQ <- paste0("dataN$", names[jj], " <- rbinom(", nh, ", 1, 0.40)")QQ <-
      eval(parse(text = QQ))
    }
```

© 2021 Asch DA et al. *JAMA Network Open*

```
if(names[jj] %!in% c("gender", "snf_nursing_flag", "mbr_composite_flag", "hosp_name","size",
                                          "count_day_class", "age_class")){

    set.seed(jj + 1000)

    prob_c <- runif(1, 0.3, 0.7)

    QQ <- paste0("dataN$", names[jj], " <- rbinom(", nh, ", 1, prob_c)")QQ <-
    eval(parse(text = QQ))

}

if(names[jj] == "age_class"){

    set.seed(jj + 3000)
```

© 2021 Asch DA et al. *JAMA Network Open*

```
        dataN$age <- round(rnorm(nh, mean = 65, sd = 20)) # mean age 65 and sd 20

        dataN$age_scale <- (dataN$age - min(dataN$age)) / (max(dataN$age) - min(dataN$age))

    }

    if(names[jj] == "count_day_class"){

        set.seed(jj + 5000)

        dataN$count_day <- round(runif(nh, min = 10, max = 270)) # minimum count of day 10 and maximum 270

        dataN$count_day_scale <- (dataN$count_day - min(dataN$count_day)) / (max(dataN$count_day) - min(dataN$count_day))

    }

} # loop for each variable ends
#as.data.frame(dataN)


tow <- names[names %!in% c('gender', 'snf_nursing_flag', 'size', 'mbr_composite_flag', 'hosp_name','count_day_class',
                           'age_class', "mbr_income_class", "race_cd")]


set.seed(kk + 8899)

#bi <- rnorm(1, 0, 3) # site-specific variance (among unit)

eij1 <- rnorm(nh, 0, 0.5) # each patient-specific


eij2 <- mvrnorm(1, mu = rep(0, nh),

        Sigma = matrix(rep(9, nh ^ 2), nrow = nh) + diag(3, nh))



EE <- paste0("dataN$", tow, " * ", beta_com, "", collapse = "+")

QQ <- paste0("link_fix <- beta_int + dataN$count_day_scale * beta_count + dataN$age_scale * beta_age
              + dataN$mbr_income_scale * beta_income +dataN$snf_nursing_flag * beta_nurse +
              dataN$gender * beta_gender +dataN$race_cd * beta_race + dataN$size_scale *
              beta_size +

              ", EE, " + eij1 + eij2")QQ <-
eval(parse(text = QQ))

QQp <- (exp(QQ)/(1 + exp(QQ)))
```

© 2021 Asch DA et al. *JAMA Network Open*

```
    QQp

    dataN$mbr_composite_flag <- ifelse(QQp   >= 0.70, 1, 0)
    table(dataN$mbr_composite_flag)

    as.data.frame(dataN)

}))

# loop for each hospital ends


# Assigning category names for binary labels

for(jj in  1 : length(names)){

    if(names[jj] == "gender"){

        QQ <- paste0("dataN_All$", names[jj], " <- ifelse(dataN_All$", names[jj], " == 1,
```

© 2021 Asch DA et al. *JAMA Network Open*

```
                    'Male', 'Female')")QQ <-
  eval(parse(text = QQ))

}

if(names[jj] == "snf_nursing_flag"){

  QQ <- paste0("dataN_All$", names[jj], " <- ifelse(dataN_All$", names[jj], " == 1,'Yes', 'No')")

  QQ <- eval(parse(text = QQ))

}

if(names[jj] %!in% c("gender", "snf_nursing_flag", "mbr_composite_flag", "hosp_name","size",
        "count_day_class", "age_class", "mbr_income_class", "race_cd")){

  QQ <- paste0("dataN_All$", names[jj], " <- ifelse(dataN_All$", names[jj], " == 1,'Yes', 'No')")

  QQ <- eval(parse(text = QQ))

}

if(names[jj] == "mbr_composite_flag"){

  QQ <- paste0("dataN_All$", names[jj], " <- ifelse(dataN_All$", names[jj], " == 1,'Event', 'Alive')")

  QQ <- eval(parse(text = QQ))

}

if(names[jj] == "age_class"){

  dataN_All$age_class <- ifelse(dataN_All$age <= 55, "18 < age <= 55", ifelse(dataN_All$age
          > 55 & dataN_All$age <= 65, "55 < age <= 65",

          ifelse(dataN_All$age > 65 & dataN_All$age <= 75, "65 < age <= 75",
          ifelse(dataN_All$age > 75 & dataN_All$age <= 85,

          "75 < age <= 85", "85 < age"))))

}

if(names[jj] == "count_day_class"){

  dataN_All$count_day_class <- ifelse(dataN_All$count_day <= 90, "1 < Count <= 90",
  ifelse(dataN_All$count_day > 90 & dataN_All$count_day <= 120, "90 < Count <= 120",

  ifelse(dataN_All$count_day > 120 & dataN_All$count_day <= 150, "120 < Count <= 150",

  ifelse(dataN_All$count_day > 150 & dataN_All$count_day <= 180, "150 < Count <= 180",

                                                        "180 < Count"))))

}
```

© 2021 Asch DA et al. *JAMA Network Open*

```
if(names[jj] == "mbr_income_class"){

    dataN_All$mbr_income_class <- ifelse(dataN_All$mbr_income <= 50000, "50K > Income",
    ifelse(dataN_All$mbr_income > 50000 & dataN_All$mbr_income <= 70000, "50K <= Income < 70K",

                                                        "70K <= Income"))

}

if(names[jj] == "race_cd"){

    QQ <- paste0("dataN_All$", names[jj], " <- ifelse(dataN_All$", names[jj], " == 1,'african-american',
                        'caucasian')")
```

© 2021 Asch DA et al. *JAMA Network Open*

```
        QQ <- eval(parse(text = QQ))
    }
}


# defining reference categories

dataN_All <- dataN_All %>% mutate_if(is.character, as.factor)

dataN_All <- within(dataN_All, age_class <- relevel(age_class, ref = "18 < age <= 55"))dataN_All <-
within(dataN_All, gender <- relevel(gender, ref = "Female"))

dataN_All <- within(dataN_All, race_cd <- relevel(race_cd, ref = "caucasian")) dataN_All$mbr_income_class <-
factor(dataN_All$mbr_income_class, levels = c("50K > Income",

                                        "50K <= Income < 70K", "70K <= Income"))

dataN_All <- within(dataN_All, mbr_income_class <- relevel(mbr_income_class, ref = "70K <= Income"))dataN_All <-
within(dataN_All, count_day_class <- relevel(count_day_class, ref = "180 < Count")) dataN_All <- within(dataN_All,
snf_nursing_flag   <- relevel(snf_nursing_flag, ref = "No"))

dataN_All <- within(dataN_All, mbr_composite_flag <- relevel(mbr_composite_flag, ref = "Alive"))dataN_All$bed <- log(1
+ dataN_All$size)  # log transformation

comorb <- names[names %!in% c("gender", "snf_nursing_flag", "mbr_composite_flag", "size",

                                                  "hosp_name", "count_day_class", "age_class", "mbr_income_class", "race_cd")]

for(jj in  1 : length(comorb)){
    QQ <- paste0("dataN_All <- within(dataN_All, ", comorb, " <- relevel(",comorb, ", ref = 'No'))",collapse = "; ")

    QQ <- eval(parse(text = QQ))

}
```

# Data check

We ensure one Black and one white per site.

```r
a1 <- unique(dataN_All$hosp_name)

a2 <- unlist(lapply(seq_len(length(a1)), function(ii) {r1 <-
    which(dataN_All$hosp_name == a1[ii])

    r2 <- table(dataN_All$race_cd[r1])

    if(length(r2) < 2){

      0  # donot add this site
    }else{

      1  # add this site
     }
```

```
## a2

##      1
```

```r
## Use Non-white to filter data
if(length(which(a2 == 0)) > 1){ del_site <-
a1[which(a2 == 0)]

dataN_All <- dataN_All[which(dataN_All$hosp_name %!in% del_site), ]

# removing these sites as they don't meet 1 white/nonwhite

}



## Contingency table for race and outcomes by age

subMain <- dataN_All[, colnames(dataN_All) %in% c("mbr_composite_flag",

                                                  "race_cd", "age_class")]

Q <- rpivotTable(data = subMain,

                 cols = c("mbr_composite_flag", "race_cd"),rows =
                 c("age_class"),

                 aggregatorName = "Count",vals =
```

© 2021 Asch DA et al. *JAMA Network Open*

| Table | |
|---|---|
| Count ↕ ↔ | mbr_composite_flag ▾ race_cd ▾ |

© 2021 Asch DA et al. *JAMA Network Open*

age_class

| | mbr_composite_flag | Alive | | Event | | Totals |
|---|---|---|---|---|---|---|
| | race_cd | african-american | caucasian | african-american | caucasian | |
| age_class | | | | | | |
| **18 < age <= 55** | | 4,717 | 9,617 | 1,722 | 2,088 | **18,144** |
| **55 < age <= 65** | | 2,753 | 4,097 | 993 | 839 | **8,682** |
| **65 < age <= 75** | | 1,757 | 3,283 | 986 | 927 | **6,953** |
| **75 < age <= 85** | | 926 | 2,696 | 736 | 469 | **4,827** |
| **85 < age** | | 1,147 | 3,491 | 813 | 547 | **5,998** |
| | **Totals** | **11,300** | **23,184** | **5,250** | **4,870** | **44,604** |

© 2021 Asch DA et al. *JAMA Network Open*

## Fitting generalized linear mixed model We fit multiple generalized linear models with logit link function; the models are fitted sequentially in such a way so that any former model is nested on latter model. The saturated model refers to the one with all covariates (demographic, comorbidity, nurse, time) and hospital effects.

```
# Model-1 :   Race

system.time(fit1 <- blm_model(variable_list = "Race",

                    Data2 = dataN_All, comorb_name = list(comorb_nm, comorb_id),

                    ylist = c("mbr_composite_flag", "mbr_hospice_flag", "mbr_death_flag"),ylist_cat =
                    "mbr_composite_flag",

                    reml = FALSE, bootH = FALSE,ranlist = "(1 |
                    hosp_name)",refh = "Hospital_1",

                    state = FALSE,refs =
                    NULL,

                    mlist_race = "race_cd",

                    varlist_race = "Race::african-american", GLM = TRUE, GLM_FX
                    = FALSE, GLM_ML = FALSE,
```

```
##      user  system elapsed
....    0.60    0.00    0.59
```

```
# Model-2 :   Age + Gender + Race + Income

system.time(fit2 <- blm_model(variable_list = "Age:Gender:Race:Income",

                    Data2 = dataN_All, comorb_name = list(comorb_nm, comorb_id),

                    ylist = c("mbr_composite_flag", "mbr_hospice_flag", "mbr_death_flag"),ylist_cat =
                    "mbr_composite_flag",

                    reml = FALSE, bootH = FALSE,ranlist = "(1 |
                    hosp_name)",refh = "Hospital_1",

                    state = FALSE,refs =
                    NULL,

                    mlist_race = "race_cd",

                    varlist_race = "Race::african-american", GLM = TRUE, GLM_FX
                    = FALSE, GLM_ML = FALSE,GLMM_RE = FALSE, laplace =
                    [illegible]
```

```
##      user  system elapsed
....    0.45    0.00    0.47
```

© 2021 Asch DA et al. *JAMA Network Open*

```
# Model-3 : Age + gender + race + comorbidities + nurse + time

system.time(fit3  <-  blm_model(variable_list  =  "Age:Gender:Race:Income:Nurse:Comorbidity:Count",

                                Data2 = dataN_All, comorb_name = list(comorb_nm, comorb_id),

                                ylist = c("mbr_composite_flag", "mbr_hospice_flag", "mbr_death_flag"),ylist_cat =
                                "mbr_composite_flag",

                                reml = FALSE, bootH = FALSE,ranlist = "(1 |
                                hosp_name)",refh = "Hospital_1",

                                state = FALSE,refs =
                                NULL,

                                mlist_race = "race_cd",

                                varlist_race = "Race::african-american", GLM = TRUE, GLM_FX
                                = FALSE, GLM_ML = FALSE,GLMM_RE = FALSE, laplace =
                                FALSE))
```

```
##     user  system elapsed
....    1.64    0.04    1.69
```

```
# Model-4 : Age + gender + race + comorbidities + nurse + time + hospital

system.time(fit4  <-  blm_model(variable_list  =  "Age:Gender:Race:Income:Nurse:Comorbidity:Count",

                                Data2 = dataN_All, comorb_name = list(comorb_nm, comorb_id),

                                ylist = c("mbr_composite_flag", "mbr_hospice_flag", "mbr_death_flag"),ylist_cat =
                                "mbr_composite_flag",

                                reml = FALSE, bootH = FALSE,ranlist = "(1 |
                                hosp_name)",refh = "Hospital_1",

                                state = FALSE,refs =
                                NULL,

                                mlist_race = "race_cd",

                                varlist_race = "Race::african-american", GLM = TRUE,
                                GLM_FX = TRUE, GLM_ML = FALSE,GLMM_RE = FALSE,
                                laplace = FALSE))
```

```
## Warning: glm.fit: fitted probabilities numerically 0 or 1 occurred
```

```
##     user  system elapsed##
473.11  [illegible]  477.00
```

© 2021 Asch DA et al. *JAMA Network Open*

# Fitting generalized linear mixed model

Following the procedures described in Drye et al. and Silber et al., we compute hospital specific riskstandardized rates (RSERs) or risk standardized mortality rates (RSMRs). In this example, we focus only on composite outcomes (i.e., RSERs).

```
# Hierarchical model (Generalized linear mixed model)

system.time(fit5 <- blm_model_hypothetical(

                    variable_list = "Age:Gender:Income:Nurse:Comorbidity:Count",Data2 = dataN_All,
                    comorb_name = list(comorb_nm, comorb_id),

                    ylist = c("mbr_composite_flag", "mbr_hospice_flag", "mbr_death_flag"),ylist_cat =
                    "mbr_composite_flag",

                    reml = FALSE,
                    maxnum = 2,

                    ranlist = "(1 | hosp_name)",refh =
                    "Hospital_1",

                    refs = NULL,

                    mlist_race = "race_cd",
```

```
##       user   system elapsed##
245.66  2.54  248.36
```

# Association effect

We display the adjusted odds ratio in a forest plot along with 95% CI and p-values for nested models.

© 2021 Asch DA et al. *JAMA Network Open*

```
for(jj in  1 : 4){

if(jj < 3){topr <- 15} else{topr <- 2}

if(jj == 4){GG <- 'glm_w'}else{GG <- 'glm_wot'}QQ <-
paste0("plotOR_blm(fit = fit", jj,",

                    fit_type = GG, mlist_race =
                    'race_cd',

                    varlist_race = 'Race::Black',reflist_race =
                    'White',

                    comorb_name = list(comorb_nm, comorb_id),ylist =
                    c('mbr_composite_flag',

                                  'mbr_hospice_flag','mbr_death_flag'),

                    resp_type = 'composite',

                    variable_list = 'Age:Gender:Race:Income:Nurse:Comorbidity:Count',state = FALSE,

                    xl = 0.2, xu = 15,

                    txtcol = paste0('No. composite/\nCOVID patients'),xlab_title =
                    paste0('Adjusted odds ratio'),

                    topr = topr)")

QQ <- eval(parse(text = QQ))
```

© 2021 Asch DA et al. *JAMA Network Open*

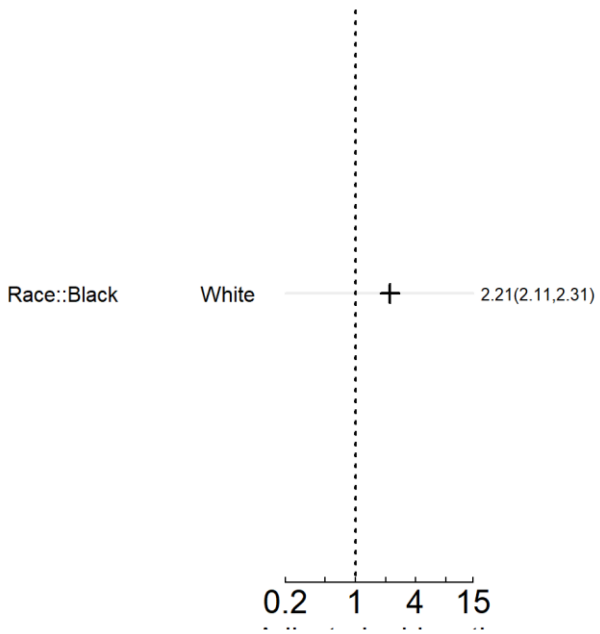


© 2021 Asch DA et al. *JAMA Network Open*

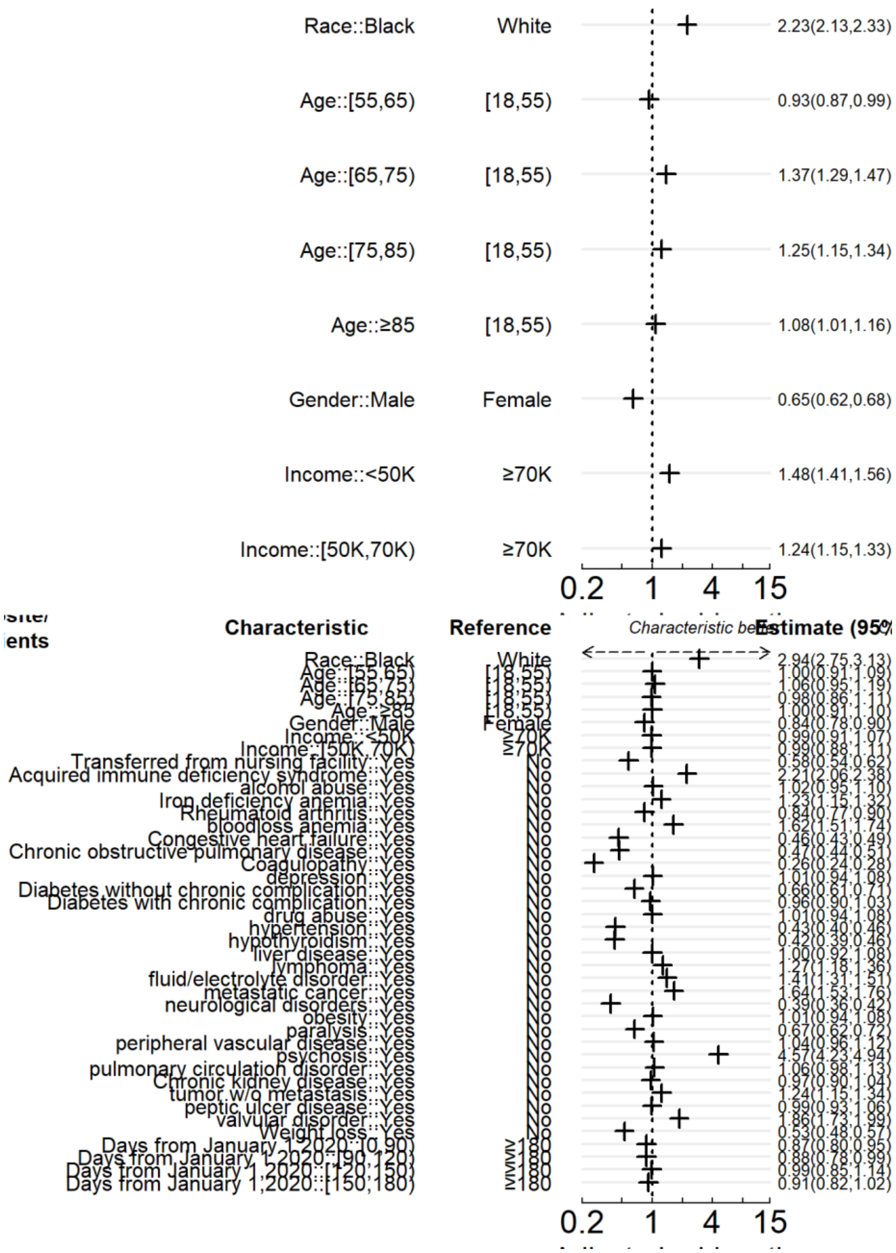


© 2021 Asch DA et al. *JAMA Network Open*

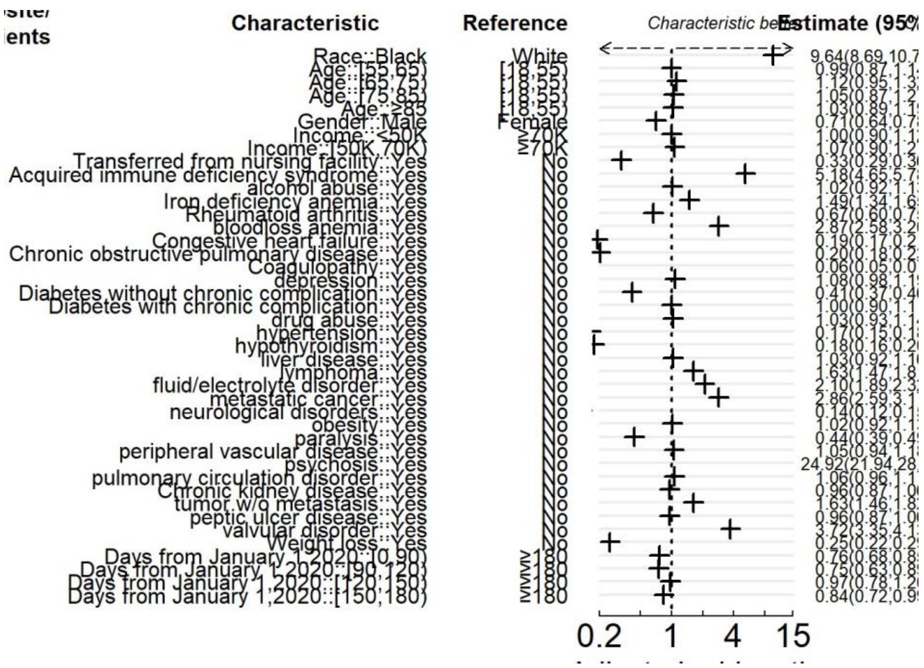

# Predicted probabilities of mortality

We provide the histograms of recylced predictions for Black and white patients. This is based on the assumption that each patient, hypothetically, had been Black and white.

© 2021 Asch DA et al. *JAMA Network Open*

```
mean_pred_blk <- mean_pred_wht <- p1 <- p2 <- p3 <- p4 <- list()

for(jj in  1 : 4){


  if(jj == 4){

    QQ <- paste0("WW <- try(SM_FIT(fitr = fit", jj,", fixed = TRUE, hospital = TRUE, mu1 = 10))")

    # Difference of mu1 = 10 is negligible

  }else{

    QQ <- paste0("WW <- try(SM_FIT(fitr = fit", jj,", fixed = TRUE, hospital = FALSE, mu1 = 10))")

    # Difference of mu1 = 10 is negligible

  }

  QQ <- eval(parse(text = QQ))


  wgh_wht <- 1 / (WW$pfit_wht_se ^ 2);
  wgh_blk <- 1 / (WW$pfit_blk_se ^ 2)
  num_wht <- WW$pfit_wht * wgh_wht;
  num_blk <- WW$pfit_blk * wgh_blk

  thet_wht <- sum(num_wht) / sum(wgh_wht); # weighted mean

  thet_blk <- sum(num_blk) / sum(wgh_blk); # weighted mean


  var_thet_wht <- 1 / sum(wgh_wht);
  var_thet_blk <- 1 / sum(wgh_blk)


  lb_thet_wht <- thet_wht - 1.96 * sqrt(var_thet_wht)
  ub_thet_wht <- thet_wht + 1.96 * sqrt(var_thet_wht)


  lb_thet_blk <- thet_blk - 1.96 * sqrt(var_thet_blk)ub_thet_blk <-
  thet_blk + 1.96 * sqrt(var_thet_blk)


  # White
```

© 2021 Asch DA et al. *JAMA Network Open*

```
mean_pred_wht[[jj]] <- data.frame("Weighted mean" = exp(thet_wht) / (1 + exp(thet_wht)),"Lower weighted
                    mean" = exp(lb_thet_wht) / (1 + exp(lb_thet_wht)),

                    "Upper weighted mean" = exp(ub_thet_wht) / (1 + exp(ub_thet_wht))) * 100


# Black

mean_pred_blk[[jj]] <- data.frame("Weighted mean" = exp(thet_blk) / (1 + exp(thet_blk)),"Lower weighted
                    mean" = exp(lb_thet_blk) / (1 + exp(lb_thet_blk)),

                    "Upper weighted mean" = exp(ub_thet_blk) / (1 + exp(ub_thet_blk))) * 100


# p-1

p1[[jj]] <- as.ggplot(function()
```

© 2021 Asch DA et al. *JAMA Network Open*

```
hist(WW$pred_wht,
    col = "gray",
    breaks = 40,

    xlab = "Predicted probability (White)",border =
    "gray",

    xlim = c(0, 1), ylim =
    c(0, 8000),cex.lab =
    1.1,

    cex.axis = 1.1,

    prob = FALSE, # show densities instead of frequencies

    main = paste0("")))


# p-2

p2[[jj]] <- as.ggplot(function()
hist(WW$pred_blk,

    col = "gray",

    xlab = "Predicted probability (Black)",border =
    "gray",

    breaks = 40, xlim
    = c(0, 1),

    ylim = c(0, 8000),
    cex.lab = 1.1,

    cex.axis = 1.1,

    prob = FALSE, # show densities instead of frequencies

    main = paste0("")))


# p-3

dat <- data.frame(Probability = c(WW$pred_wht,

                                  WW$pred_blk),
                  Race = rep(c("White", "Black"),

                             each = length(WW$pred_wht)))
p3[[jj]] <- ggplot2::ggplot(dat,

               aes(x = Probability, fill = Race)) +
geom_density(alpha = 0.5) + xlim(0, 1) +
```

© 2021 Asch DA et al. *JAMA Network Open*

```
theme_minimal() + labs(x = "Probability") + labs(y =
"Density") +

theme(axis.text.x = element_text(size = 14),axis.text.y =
          element_text(size = 14),axis.title.y =
          element_text(size = 16),axis.title.x =
          element_text(size = 16),axis.title =
          element_text(size = 14),
```

© 2021 Asch DA et al. *JAMA Network Open*

```
            legend.text = element_text(size = 14)) +
    theme(legend.position = "top")

}


for(jj in  1 : 4){

lym <- matrix(c(1, 1, 2, 2, 3, 3, 3, 3), nrow = 1, ncol = 8)

gridExtra::grid.arrange(p1[[jj]],   p2[[jj]],   p3[[jj]],

                                       layout_matrix = lym)
print(mean_pred_wht[[jj]]) print(mean_pred_blk[[jj]])
```

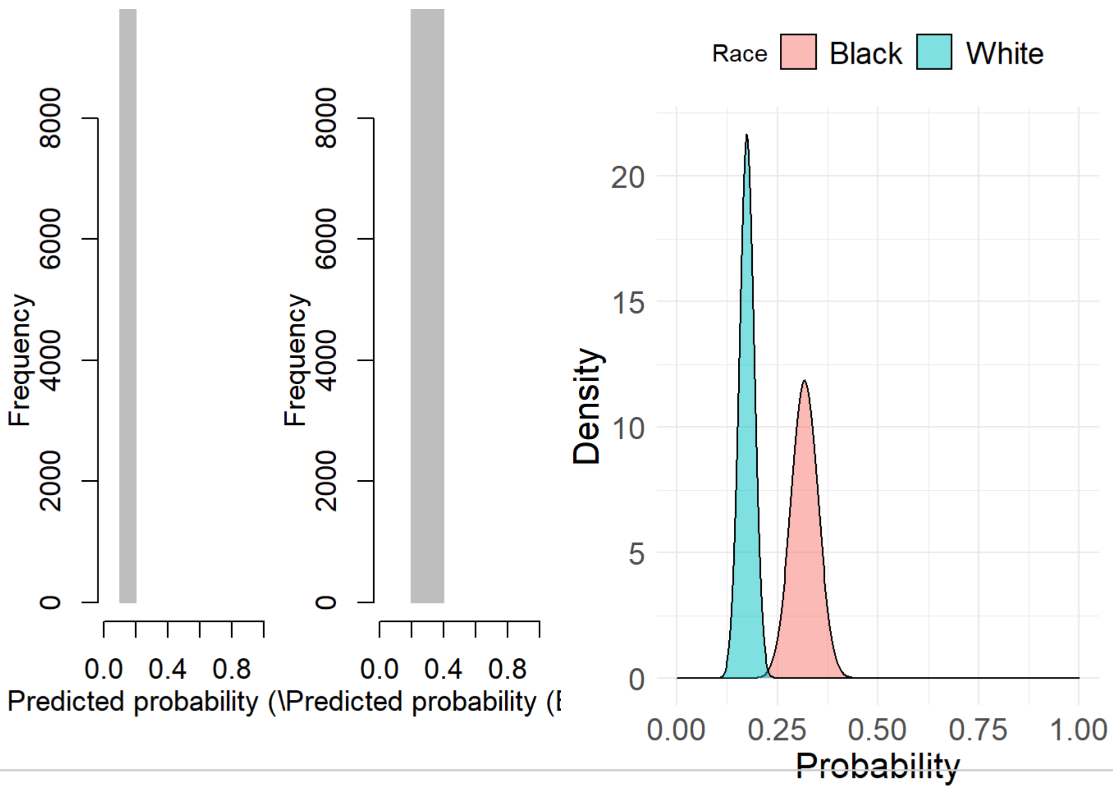


```
##      Weighted.mean  Lower.weighted.mean  Upper.weighted.mean
## 1         17.35938             17.35728             17.36148
## ## Weighted.mean  Lower.weighted.mean  Upper.weighted.mean
1         31.72205              31.7187             31.72541
```

© 2021 Asch DA et al. *JAMA Network Open*

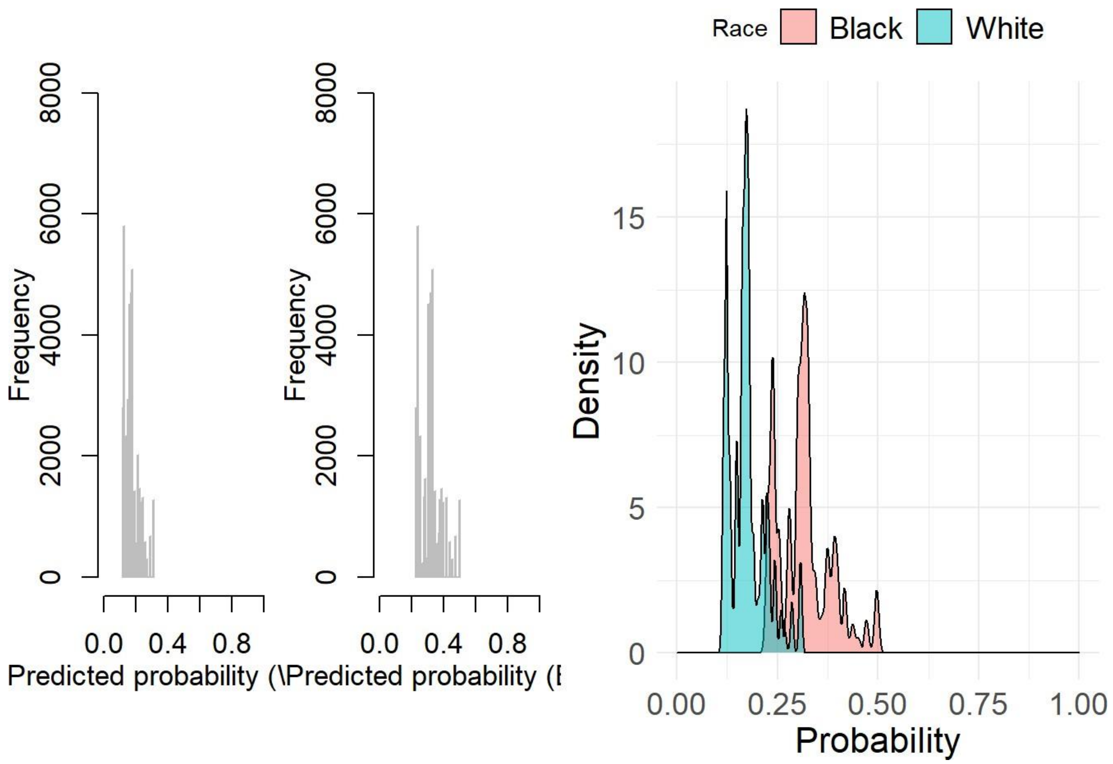


```
##       Weighted.mean  Lower.weighted.mean  Upper.weighted.mean
## 1          16.42484             16.42074             16.42893
## ## Weighted.mean  Lower.weighted.mean  Upper.weighted.mean
1          30.43324             30.42676             30.43972
```

© 2021 Asch DA et al. *JAMA Network Open*

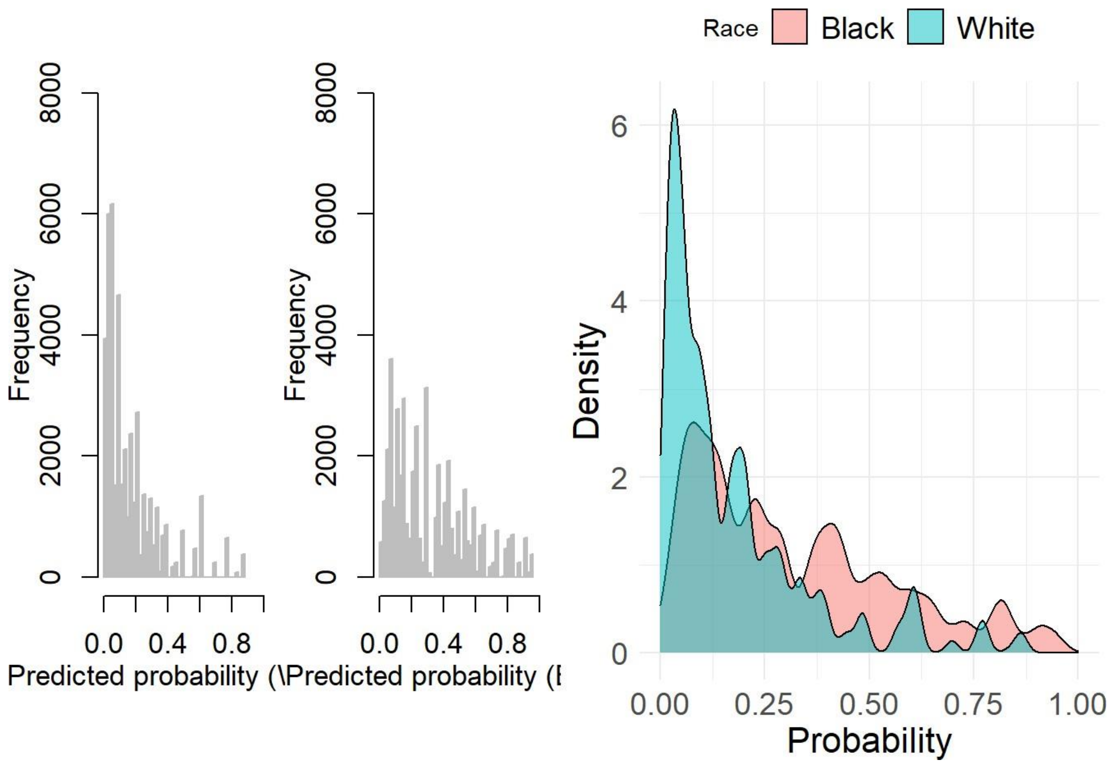


```
##       Weighted.mean   Lower.weighted.mean   Upper.weighted.mean
## 1         14.05109              14.04109              14.06109
## ##    Weighted.mean   Lower.weighted.mean   Upper.weighted.mean
1
          31.49504              31.47706              31.51303
```

© 2021 Asch DA et al. *JAMA Network Open*

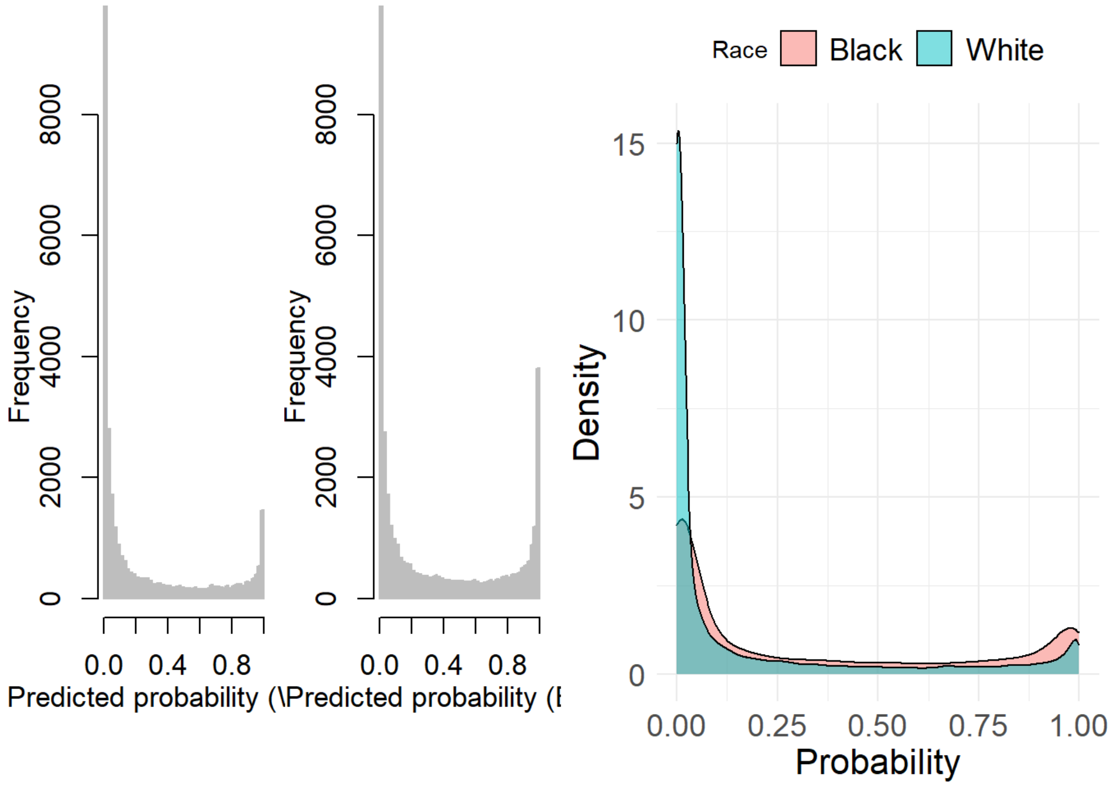


```
##      Weighted.mean   Lower.weighted.mean   Upper.weighted.mean
## 1          6.247784              6.221871              6.273797
## ## Weighted.mean   Lower.weighted.mean   Upper.weighted.mean
1          38.08558              37.98113              38.19014
```

# LRT for nested models

```
Lrt<-BIC<-Mcf<-Hsm<-list()

Lrt <- list("Model 1 vs 2" = lrtest(fit2$fom_wot_hosp, fit1$fom_wot_hosp), "Model 2 vs 3" =
        lrtest(fit3$fom_wot_hosp, fit2$fom_wot_hosp),"Model 3 vs 4" = lrtest(fit4$fom_w_hosp,
        fit3$fom_wot_hosp))
```

© 2021 Asch DA et al. *JAMA Network Open*

# Summary measures for model performance

© 2021 Asch DA et al. *JAMA Network Open*

```
# Pseudo R-squared

Mcf <- list("Model 1" = pR2(fit1$fom_wot_hosp), "Model 2" =
            pR2(fit2$fom_wot_hosp),     "Model     3"     =
            pR2(fit3$fom_wot_hosp),     "Model     4"     =
            pR2(fit4$fom_w_hosp))
```

```
## fitting null model for pseudo-r2 ## fitting null
model for pseudo-r2## fitting null model for pseudo-
r2## fitting null model for pseudo-r2
```

```
# Hosmer-lemeshow

bin <- 10                    # number of groups

Hsm <- list("Model 1" = hoslem.test(fit1$Data22$mbr_composite_num, fitted(fit1$fom_wot_hosp), g = bin), "Model 2" =
            hoslem.test(fit2$Data22$mbr_composite_num,   fitted(fit2$fom_wot_hosp),   g   =   bin),   "Model   3"   =
            hoslem.test(fit3$Data22$mbr_composite_num,   fitted(fit3$fom_wot_hosp),   g   =   bin),   "Model   4"   =
            hoslem.test(fit4$Data22$mbr_composite_num, fitted(fit4$fom_w_hosp), g = bin))


# C-statistics and Somer's Dxy

Cstat <- list("Model 1" = .somers2(fitted(fit1$fom_wot_hosp), as.numeric(fit1$Data22$mbr_composite_num)), "Model 2" =
              .somers2(fitted(fit2$fom_wot_hosp),    as.numeric(fit2$Data22$mbr_composite_num)),    "Model    3"    =
              .somers2(fitted(fit3$fom_wot_hosp),    as.numeric(fit3$Data22$mbr_composite_num)),    "Model    4"    =
              .somers2(fitted(fit4$fom_w_hosp), as.numeric(fit4$Data22$mbr_composite_num)))


# Asymptomatic Likelihood Ratio Test (LRT)

data.frame("Model" = c("Model 1 vs 2", "Model 2 vs 3", "Model 3 vs 4"),"p-values for LRT"
           = c(Lrt[[1]]$`Pr(>Chisq)`[2],

                                Lrt[[2]]$`Pr(>Chisq)`[2],

                                Lrt[[3]]$`Pr(>Chisq)`[2]))
```

© 2021 Asch DA et al. *JAMA Network Open*

```
##                Model p.values.for.LRT
## 1 Model 1 vs 2        1.853311e-152
## 2 Model 2 vs 3         0.000000e+00
```

© 2021 Asch DA et al. *JAMA Network Open*

```
# Measure for goodness of fits

data.frame("Models" = c("Model 1", "Model 2", "Model 3", "Model 4"),"McFadden" = c(Mcf[[1]][4],
Mcf[[2]][4], Mcf[[3]][4], Mcf[[4]][4]),

"Cragg-and-Uhler" = c(Mcf[[1]][6], Mcf[[2]][6], Mcf[[3]][6], Mcf[[4]][6]),

"Hosmer-lemeshow" = c(Hsm[[1]]$p.value, Hsm[[2]]$p.value, Hsm[[3]]$p.value, Hsm[[4]]$p.value),

"C-statistics" = c(Cstat[[1]][1], Cstat[[2]][1], Cstat[[3]][1], Cstat[[4]][1])
```

```
##      Models    McFadden  Cragg.and.Uhler  Hosmer.lemeshow  C.statistics  Somer.Dxy
##  1 Model 1   0.02499741       0.04018766     1.000000e+00     0.5955433  0.1910865
##  2 Model 2   0.04019190       0.06409485     0.000000e+00     0.6273775  0.2547549
##  3 Model 3   0.23950989       0.34420041     4.055594e-01     0.8207602  0.6415203
##  4 Model 4   0.62848636       0.74524926     9.708221e-07     0.9640852  0.9281705
```

# Summary RSERs

```
fitr <- fit5 hist(fitr$score_silber * 100,

     col = "gray",

     xlab = "Distribution of risk standardized event rates (%)",border = "gray",

     xlim = c(0, 100),ylim = c(0,
     400),cex.lab = 1.5,

     cex.axis = 1.5, prob =
     FALSE, main = paste0(""))
```

© 2021 Asch DA et al. *JAMA Network Open*

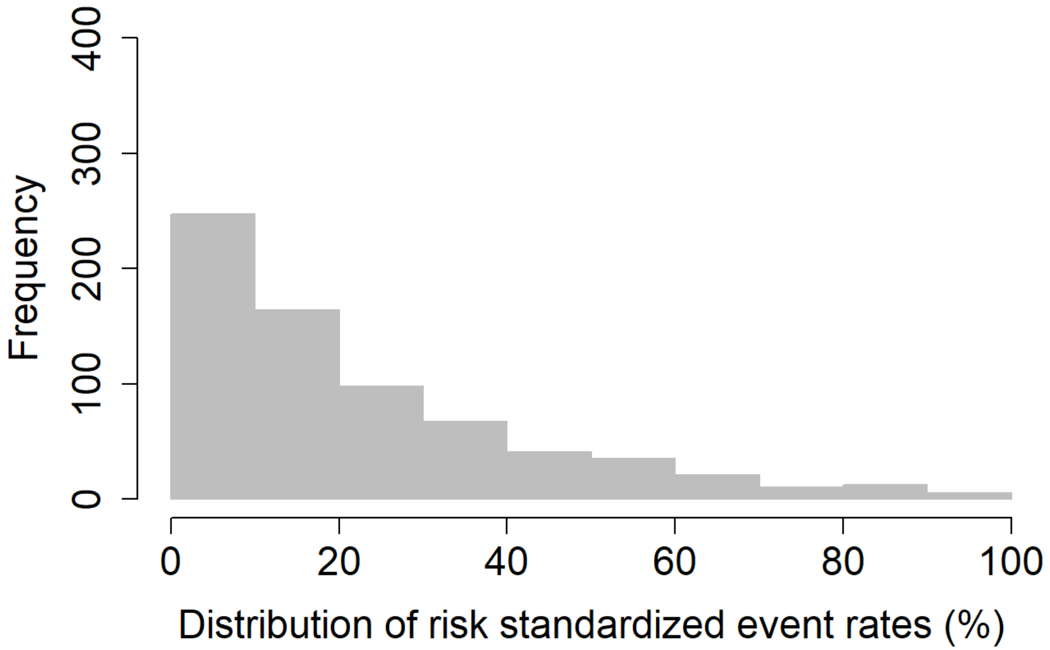


```
plot.new() summary_statistics_table(fitr$score_silber   *   100)
```

© 2021 Asch DA et al. *JAMA Network Open*

| | Summary statistics |
|---|---|
| *Number* | 700.00 |
| *Minimum* | 0.57 |
| *Maximum* | 97.00 |
| *Median* | 16.38 |
| *Mean* | 22.44 |
| *SE of mean* | 0.77 |
| *Variance* | 416.47 |
| *IQR* | 25.94 |
| *95% CI for mean* | (20.93, 23.95) |

© 2021 Asch DA et al. *JAMA Network Open*

# Numerical experiment

We conduct a numerical experiment where we distribute Black patients in different hospitals in a way white patients are distributed in the sample while we retain Black patients' clinical and demographic attributes unperturbed. We repeat the process 500 times and report the mean improvement in Black mortality rate.

```
fitrd <- fitr$Data22; dim(fitrd)
```

```
## [1] 44604      38
```

© 2021 Asch DA et al. *JAMA Network Open*

```
totalb <- table(fitrd$race_cd); totalb
```

```
##
##        caucasian   african-american
##              28054              16550
```

```
totalw <- totalb[which(names(totalb) %!in% "african-american")]; totalw
```

```
## caucasian##
         28054
```

© 2021 Asch DA et al. *JAMA Network Open*

```
totalb <- totalb[which(names(totalb) %in% "african-american")]nameb <-
data.frame("reference" = fitr$reference,

                                        "silber" = fitr$score_silber)

I <- nrow(nameb)


nameb$whitepop <- unlist(lapply(seq_len(I), function(ii) {

    gg <- fitrd$race_cd[which(fitrd$hosp_name %in% nameb$reference[ii])] # which belongs to this site

    lw <- length(gg[which(gg %!in% "african-american")]) # which for this site belong to black/white cohort

    lw

}))

nameb$blackpop <- unlist(lapply(seq_len(I), function(ii) {

    gg <- fitrd$race_cd[which(fitrd$hosp_name %in% nameb$reference[ii])] # which belongs to this site

    lw <- length(gg[which(gg %in% "african-american")]) # which for this site belong to black/white cohort

    lw

}))

nameb$whiteprop <- nameb$whitepop / totalw
nameb$blackprop <- nameb$blackpop / totalb


# Weighted mean for black patient mortality

QQ <- unlist(lapply(seq_len(I), function(ii) {

    died_i <- length(which(fitr$Data22$race_cd == "african-american" & fitr$Data22$hosp_name == nameb$reference[ii] & fitr$Data22$mbr_composite_flag
== "Event"))

    total_i <- length(which(fitr$Data22$race_cd == "african-american" & fitr$Data22$hosp_name == nameb$reference[ii]))paste0("", died_i ,"/",
    total_i, "")

}))

nameb$mbr_composite_flag_black <- unlist(lapply(seq_len(I), function(ii) {

    died_i <- length(which(fitr$Data22$race_cd == "african-american" & fitr$Data22$hosp_name == nameb$reference[ii] & fitr$Data22$mbr_composite_flag
== "Event"))

    total_i <- length(which(fitr$Data22$race_cd == "african-american" & fitr$Data22$hosp_name == nameb$reference[ii]))died_i / total_i
```

© 2021 Asch DA et al. *JAMA Network Open*

```
}))
head(nameb)
```

© 2021 Asch DA et al. *JAMA Network Open*

```
##      reference      silber whitepop blackpop    whiteprop    blackprop
## 1 Hospital_1 0.54206478       29       14 0.0010337207 0.0008459215
## 2 Hospital_2 0.19309147       59       38 0.0021030869 0.0022960725
## 3 Hospital_3 0.42851194       67       41 0.0023882512 0.0024773414
## 4 Hospital_4 0.16544057       13        6 0.0004633920 0.0003625378
## 5 Hospital_5 0.07466568        8        3 0.0002851643 0.0001812689
## 6 Hospital_6 0.03735454       72       42 0.0025664789 0.0025377644
##   mbr_composite_flag_black
## 1               0.57142857
## 2               0.34210526
## 3               0.56097561
## 4               0.33333333
## 5               0.00000000
## 6               0.04761905
```

```
ybar_black_mortality <- sum(nameb$mbr_composite_flag_black * nameb$blackprop) # black mortality weighted by black proportion

ybar_black_mortality # Observed weighted average for Black mortality
```

```
## [1] 0.3172205
```

```
mean(nameb$mbr_composite_flag_black) # Observed un-weighted average for Black mortality
```

```
## [1] 0.2961353
```

```
# Simulate Black by white proportion

NB <- 500

system.time(carg_blk <- counterfactual_RSER(A = 232323, I, nameb, totalb, totalw,
```

```
##   user  system elapsed
## 177.83  0.31  178.36
```

```
# Simulate white by Black proportion

#system.time(carg_wht <- counterfactual_RSER(A = 232323, I, nameb, totalb, totalw,
#                         NB = NB, fitr, method = "1-step", varlist_race = "caucasian"))
```

```
pihat <- mean(unlist(carg_blk$mean_counter_patient))

pihat * 100 # estimated mortality rate for Black based on simulation
```

```
## [1] 25.63879
```

```
carg_mat_blk <- do.call(cbind, lapply(seq_len(NB), function(bb)carg_blk$counter_patient[[bb]]))

carg_i_blk <- apply(carg_mat_blk, 1, mean) carg_i_blk_sim <-
apply(carg_mat_blk, 2, mean) diff_mean <- c(ybar_black_mortality
- carg_i_blk_sim)


datam <- data.frame(name = c(rep(NA, NB)), "increment" = diff_mean * 100)p4 <-
ggplot2::ggplot(data = datam, aes(x = name, y = increment)) +

  geom_boxplot(outlier.shape = NA) +

  geom_jitter(alpha = 0.2, position = position_jitter(width = 0.08, height = 0.01,

                                                       seed = 521)) +

  theme_minimal() +

  labs(title = "",

       y = "Net improvement (%)") +

  theme(axis.text.x = element_text(size = 14, colour = "white"),axis.text.y =
        element_text(size = 14),

        axis.title.y = element_text(size = 14),axis.title.x =
        element_text(size = 14),axis.title =
        element_text(size = 14), legend.text =
        element_text(size = 14)) +

  scale_y_continuous(breaks = seq(5, 7, by = 0.40),

                     limits = c(5, 7)) +

  labs(x = "") +
```

© 2021 Asch DA et al. *JAMA Network Open*

```
stat_summary(fun.y = mean, geom = "point",
             shape = 18, size = 5, alpha = 1,color =
             "firebrick2")
```

```
## Warning: `fun.y` is deprecated. Use `fun` instead.
```

© 2021 Asch DA et al. *JAMA Network Open*

```
# Simulated mortality rates for Black and net improvment

dat <- data.frame(Probability = c(carg_i_blk * 100),
                  Legends = rep(c("Blacks go to hospitals where whites typically go to"),each =
                            length(carg_i_blk)))


p5 <- ggplot2::ggplot(dat, aes(x = Probability, fill = Legends)) +
  scale_fill_manual(values = c("#999999")) +
  geom_density(alpha = 0.5, show.legend = FALSE) +
  theme_minimal() +
  theme(axis.text.x = element_text(size = 14), axis.text.y =
        element_text(size = 14), axis.title.y =
        element_text(size = 16),axis.title.x =
        element_text(size = 16),axis.title =
        element_text(size = 14), legend.text =
        element_text(size = 14)) +
  theme(legend.title = element_blank()) +
  scale_x_continuous("Probability (%) of death or referral to hospice within 30 days",limits = c(0, 100),
                     labels = paste0("",seq(0, 100, 10),""),
                     breaks = seq(0, 100, 10)) +
  annotate("text", x = (mean(carg_i_blk) * 100 - 4),
           y = 0.05, col = "black",
           label = sprintf("%0.2f", mean(carg_i_blk) * 100)) +
  annotate("text", x = (ybar_black_mortality * 100 + 4),
         y = 0.05, col = "black",
         label = sprintf("%0.2f", ybar_black_mortality * 100)) +
  annotate("text", x = 15.5,
         y = 0.025, col = "black",
         label = paste0("Observed event rate \nfor Black patients")) +annotate("text",
  x = 50,
         y = 0.035, col = "black",
         label = paste0("Estimated event rate for \nBlack patients distributed to \nhospitals as white \npatients were"))
 +
  geom_vline(xintercept = mean(carg_i_blk) * 100, linetype = 5, col =
           "black", size = 1.1) +
```

© 2021 Asch DA et al. *JAMA Network Open*

```
geom_vline(xintercept = ybar_black_mortality * 100, linetype = 1,
                      col = "black", size =   1.2) +
labs(y = "Density") +
geom_segment(

    x = ybar_black_mortality * 100, y = 0.05,
```

© 2021 Asch DA et al. *JAMA Network Open*

```
        xend = mean(carg_i_blk) * 100, yend = 0.05,lineend = "butt",

        linejoin = "round",size = 1.5,

        arrow = arrow(length = unit(0.2, "inches")),colour = "red") +

    geom_segment(x = ybar_black_mortality * 100, y = 0.01,xend =
        mean(carg_i_blk) * 100, yend = 0.01,

        lineend = "butt",linejoin =
        "round",size = 1.5,

        arrow = arrow(length = unit(0.2, "inches")),colour = "red")


lym <- matrix(c(1, 1, 1, 2), nrow = 1, ncol = 4)gridExtra::grid.arrange(p5,
p4, layout_matrix = lym)
```

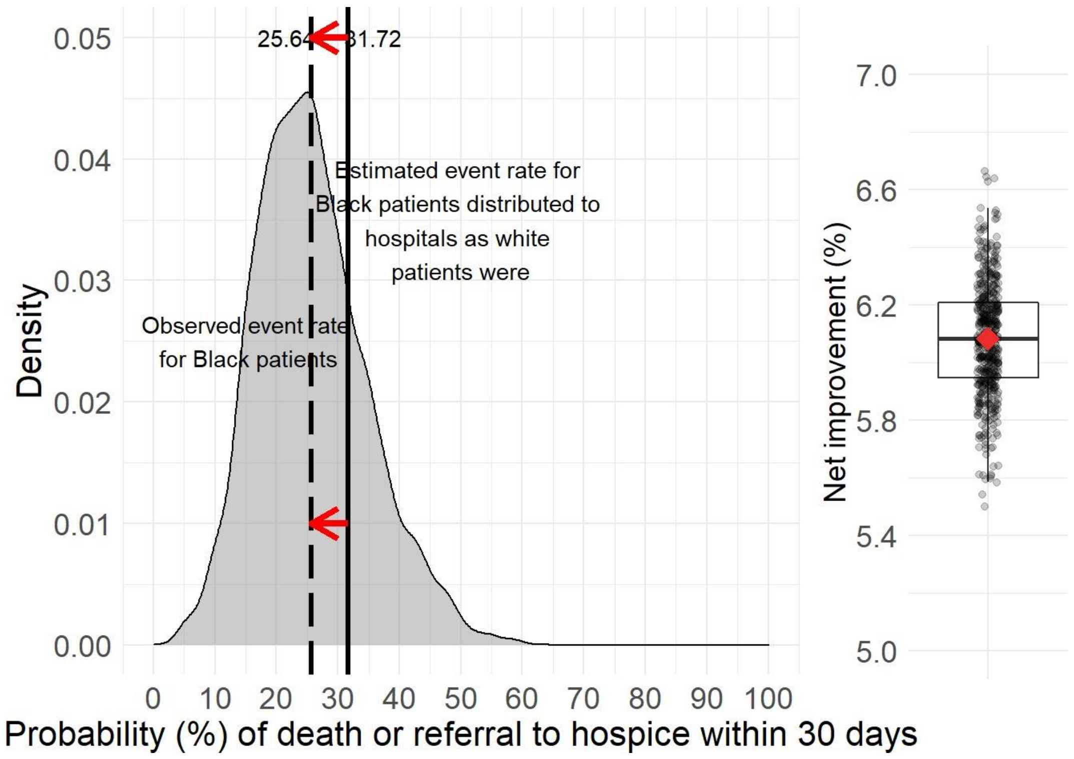


© 2021 Asch DA et al. *JAMA Network Open*

```
# Mean net improvment in mortality rate
data.frame("Net_improvement" = mean(diff_mean) * 100,
           "Net_2.5th" = quantile(diff_mean, 0.025) * 100, # 2.5th  percentile
```

```
##          Net_improvement Net_2.5th Net_97.5th
[illegible]        6.083265  5.692212   6.468339
```

© 2021 Asch DA et al. *JAMA Network Open*

**eReferences.**


1. McCullagh, Peter. Generalized linear models. Routledge, 2018.
2. Broström G, Holmberg H. Generalized linear models with clustered data: Fixed and random effects models. Computational Statistics & Data Analysis. 2011;55(12): 3123-34.
3. Pinheiro JC, Bates DM. Approximations to the log-likelihood function in the nonlinear mixed-effects model. Journal of computational and Graphical Statistics. 1995; 4(1): 12-35.
4. Silber JH, Rosenbaum PR, Brachet TJ, et al. The Hospital Compare mortality model and the volume-outcome relationship. Health Serv Res. 2010;45(5 Pt 1):1148-1167. doi:10.1111/j.1475-6773.2010.01130.x
5. Silber JH, Satopää VA, Mukherjee N, et al. Improving Medicare's Hospital Compare Mortality Model. Health Serv Res. 2016;51 Suppl 2(Suppl 2):1229-1247. doi:10.1111/1475-6773.12478
6. Silber JH, Rosenbaum PR, Niknam BA, et al. Comparing Outcomes and Costs of Surgical Patients Treated at Major Teaching and Nonteaching Hospitals: A National Matched Analysis. Ann Surg. 2020;271(3):412-421. doi:10.1097/SLA.0000000000003602
7. Rizopoulos D. "GLMMadaptive: Generalized Linear Mixed Models using Adaptive Gaussian Quadrature." R package version 0.5-1. URL: https://CRAN. R-project. org/package= GLMMadaptive (2019).


© 2021 Asch DA et al. *JAMA Network Open*
